# Synthesis and Docking Studies of Novel Spiro[5,8-methanoquinazoline-2,3′-indoline]-2′,4-dione Derivatives

**DOI:** 10.3390/molecules29215112

**Published:** 2024-10-29

**Authors:** Tünde Faragó, Rebeka Mészáros, Edit Wéber, Márta Palkó

**Affiliations:** 1 Institute of Pharmaceutical Chemistry, Interdisciplinary Excellence Center, Faculty of Pharmacy, University of Szeged, Eötvös utca 6, H-6720 Szeged, Hungary; f.tunde94@gmail.com (T.F.); meszaros.rebeka.ildiko@szte.hu (R.M.); 2 Department of Medical Chemistry, Faculty of Medicine, University of Szeged, Dóm tér 8, H-6720 Szeged, Hungary; weber.edit@med.u-szeged.hu; 3 HUN-REN-SZTE Biomimetic Systems Research Group, Dóm tér 8, H-6720 Szeged, Hungary

**Keywords:** 2-aminonorbornene carboxamide, isatin, click reaction, HSBM, MW, docking studies

## Abstract

In this study, a set of spiro[5,8-methanoquinazoline-2,3′-indoline]-2′,4-dione derivatives **3a**–**p** were synthesized starting from unsubstituted and *N*-methyl-substituted *diendo*- and *diexo*-2-aminonorbornene carboxamides, as well as various substituted isatins. The typical method involves a condensation reaction of alicyclic aminocarboxamide and isatin in the presence of a catalyst, using a solvent and an acceptable temperature. We developed a cost-effective and ecologically benign high-speed ball milling (HSBM), microwave irradiation (MW), and continuous flow (CF) technique to synthesize spiroquinazolinone molecule **3a**. The structures of the synthesized compounds **3a**–**p** were determined using 1D and 2D NMR spectroscopies. Furthermore, docking studies and absorption, distribution, metabolism, and toxicity (ADMET) predictions were used in this work. In agreement with the corresponding features found in the case of both the SARS-CoV-2 main protease (RCSB Protein Data Bank: 6LU7) and human mast cell tryptase (RCSB Protein Data Bank: 2ZA5) based on the estimated total energy and binding affinity, H bonds, and hydrophobicity in silico*,* compound **3d** among our **3a**–**g**, **3i**–**k**, and **3m** derivatives was found to be our top-rated compound.

## 1. Introduction

Because of their biological properties, oxindole and spirooxindole derivatives are highly sought after in pharmaceutical research [1,2,3,4,5]. Oxindole derivatives were generated via the aldol reaction of isatin aldehydes [6] and ketones [7,8] by reacting isatin with aromatics in triflic acid [9,10] or isatins with barbituric acid [11]. Spirooxindole compounds were synthesized in the condensation reaction of 2-aminobenzamide and isatins [12,13,14,15,16,17,18]. Another technique to synthesize spirooxindoles is to react isatoic anhydride with ammonium acetate or amines, as well as aldehydes or ketones [19,20]. In addition, other procedures for synthesizing 2,3-dihydroquinazolin-4(1*H*)-one derivatives have been reported in the literature, including the spirocyclization of aldehydes or ketones with 2-aminobenzamide [12,16]. Several catalysts, including Amberlyst 15, CuCl_2_, ZrCl_4_, NH_4_Cl, KAl(SO_4_)_2_∙12H_2_O (alum), and phosphoric acid derivatives, were evaluated to improve yield in the reaction of isatin and 2-aminobenzamide. Overall, acidic catalysts such as sulfamic acid, phosphotungstic acid, or *p*-TSA were employed with loadings ranging from 10 to 30 mol% [12,15,16,17,18,21,22,23,24,25,26,27,28]. Several substituted isatins were studied as reaction partners in this condensation reaction, yielding the corresponding racemic spiro products [12,15,16,17,18,21,22,23,24,25,26,27,28]. In general, EtOH and ACN were used as solvents. In addition, there are examples of the use of toluene and glycerol, or applying solvent-free conditions at room temperature under reflux conditions or at −45 °C [12,15,16,17,18,21,22,23,24,25,26,27]. Several approaches, including chiral catalysts (chiral phosphoric acid derivatives), green solvents (acetonitrile, acetic acid, EtOH, deep eutectic solvent mixtures), and varied temperatures, were tested to produce racemic and enantiomeric spirooxindoles [12,15,18,21,22,23,24,25,27,28,29,30,31]. Furthermore, Bergman et al. obtained a racemate of 1′*H*-spiro[indoline-3,2′-quinazoline]-2,4′(3′*H*)-dione with a yield of 88%, and the enantiomer separation was noticeable using supercritical fluid chromatography (SFC). They separated the enantiomers and determined their optical rotation, but rapid racemization of the enantiomers were recognized [32].

Some greener approaches were tested in the case of the reaction of 2-amino-benzamide and isatins, such as using gentler conditions (room temperature), ultrasonic irradiation, microwave irradiation (MW), and the use of ionic liquids and deep eutectic solvents [17,24,27]. Shabaani et al. reported a straightforward technique for synthesizing the 2,3-dihydroquinazolin-4(1*H*)-one derivative. NH_4_Cl was used as a catalyst at room temperature in EtOH in a stirred solution of 2-aminobenzamide and isatin [17]. Pal et al. used ultrasonic irradiation and Amberlyst 15 as catalyst to produce spiro-2,3-dihydroquinazolin-4(1*H*)-ones at room temperature in a few minutes [24]. Nagarajan et al. described a green approach for synthesizing quinazolinone derivatives by cyclization with aldehydes or ketones mediated by deep eutectic solvent (DES) in good to excellent yields (62–95%). They investigated various deep eutectic solvent mixtures and discovered that the molten mixture of L-(+)-tartaric acid and *N*,*N*′-dimethylurea (DMU) (3:7) at 90 °C was the most effective in producing the highest yield of spirodihydroquinazolin product [27].

Continuous flow (CF) has become a key instrument for synthetic and medicinal chemistry. CF is a rapid and easy method for chemical synthesis, and the use of reagents, solvents, and waste can be reduced with these techniques. The most critical parameters, such as flow rate, residence time, pressure, temperature, and stoichiometry, can be simply adjusted or changed [33]. In the literature, few examples can be found for the synthesis of spirooxindole-containing compounds in CF [34,35].

Energy-efficient techniques such as MW irradiation, ultrasound, and ionic liquids are well-defined. High-speed ball milling (HSBM), in turn, has received little attention in organic synthesis [36]. Purifying and disposing of dangerous toxic solvents require a significant amount of energy and consequently, they are not cost-effective. HSBM requires no or minimal solvent during the milling process. Here, mechanical energy is conveyed inside the milling jar, where solid reactants are combined [37]. There are many factors to consider, including milling time, the number and size of balls, weight, vessel radius, and the heat capacity of compounds [38].

Ten years ago, co-workers of our department used environmentally benign ways to create 2,2-disubstituted- and 2-spiroquinazolinone derivatives in either aqueous or solventless media reacting anthranilamide and a number of ketones with poor water solubility. The ring closure reaction was carried out under HSBM circumstances, with molecular iodine as catalyst [39]. However, no studies have been conducted on the analogous reaction involving alicyclic β-amino amides and ketones under HSBM conditions.

While this area has received significant attention, discovering environmentally friendly synthesis pathways to generate oxindole derivatives, such as tetrahydro-1*H*-spiro[5,8-methanoquinazoline-2,3′-indoline]-2′,4(3*H*)-dione derivatives or comparable compounds, remains a difficult task. Further research is needed to optimize the synthesis of these derivatives using spirocondensation processes, including purifying procedures as well, in order to increase yield and improve product selectivity. Furthermore, there is still a need for innovation in obtaining spiroquinazolinone derivatives using greener methodologies. Our primary goal was to synthesize novel spiroquinazolinone derivatives while simultaneously optimizing the synthesis method. We intended to investigate the reaction between racemic alicyclic *diexo*- or *diendo*-β-amino amides and isatins using more ecologically friendly approaches. Another aim was to perform an in silico study on the molecular docking of our novel compounds to two different macromolecules in order to help design presumably pharmaceutically useful molecules. Quinazolinone and spiroquinazoline derivatives possess a wide range of biological activities, such as anti-inflammatory, anticonvulsant, antiallergic, antibacterial, antihistamine, antitubercolosis, antimutagenic, and respiratory features [40,41,42]. Spiroquinazoline derivatives are ligands for several macromolecules, for example, serine/threonine kinase, tubulin, plant hormone, transferase, and hydrolase [43,44,45,46,47,48,49]. The binding of quinazolinone derivatives was also examined through docking studies to various targets [24,40,41,50,51,52]. In this study, complementing the synthesis of **3a**–**3p**, we utilized molecular docking to identify the ligands with promising binding potential to two relevant biological targets, a main protease of SARS-CoV-2 and human mast cell tryptase.

## 2. Results and Discussion

The syntheses of starting amides were performed according to the literature procedure. The *diendo*- and *diexo*-amino acids were esterified with ethanol and the ester bases, liberated from the hydrochlorides, were treated with methanolic ammonia or methyl amine to furnish amides **1a**–**d** [53,54]. We initially studied the influence of the solvent, catalyst, and temperature in the spirocondensation reaction of unsubstituted *diexo*-amide **1a** with unsubstituted isatin **2a** as a standard and model to produce 4a,5,8,8a-tetrahydro-1*H*-spiro[5,8-methanoquinazoline-2,3′-indoline]-2′,4(3*H*)-dione (**3a**). Different catalysts (30 mol% in each case), such as NH_4_Cl, KAl(SO_4_)_2_·12H_2_O (alum), LiOH, p-TsOH, Amberlyst 15, and I_2_, were tested at ambient temperature and under heating in different solvents. EtOH, glycerol, 2M2B (2-methyl-2-butanol), and water were tested as green solvents (Table 1).

Screening several catalysts, solvents, and temperatures in the reaction between **1a** and **2a** showed that the most effective ones were NH_4_Cl in 2M2B at 100 °C (after 9 h), iodine (14 h), and alum in EtOH at 78 °C (within 5 h), which afforded the product selectively (Table 1 entries 3, 11, and 16). It was necessary to heat the reaction mixture, otherwise the reaction was not completed even after a long period of time.

**Scheme 1:**
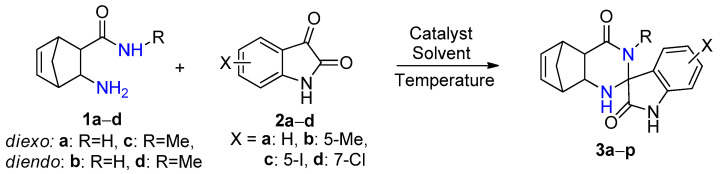
Spirocondensation reaction of *diexo*- and *diendo*-2-aminonorbornene carboxamides **1a**–**d** with unsubstituted, 5-methyl, 5-iodo- and 7-chloro-substituted isatins **2a**–**d**.

**Figure 1:**
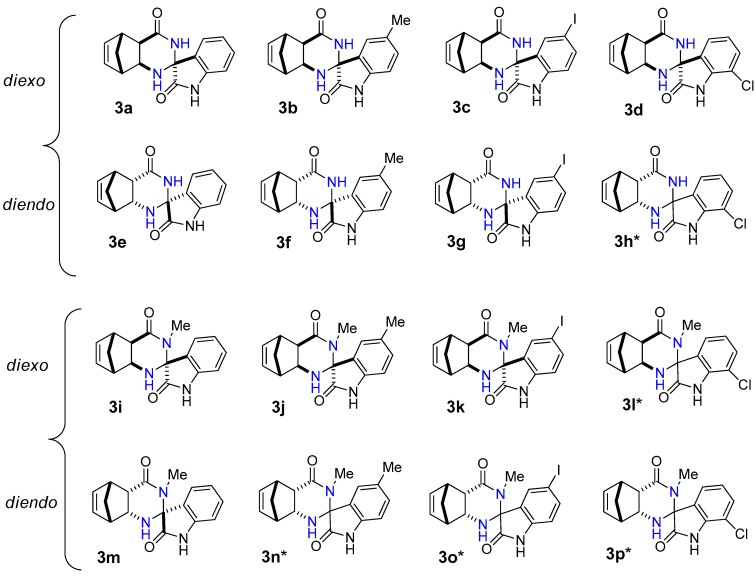
Structure of novel spiro[5,8-methanoquinazoline-2,3′-indoline]-2′,4-dione derivatives **3a**–**p**.

The reaction time was significantly shorter with alum in comparison with iodine. In addition, because alum in EtOH gave the fastest reaction affording the highest yield, subsequent syntheses were carried out in this solvent. In order to obtain novel spiro[5,8-methanoquinazoline-2,3′-indoline]-2′,4-dione derivatives **3a**–**p**, the syntheses were performed under optimized reaction conditions through a conventional method, starting from unsubstituted and methyl-substituted *diexo*- and *diendo*-2-aminonorbornene carboxamides **1a**–**d** and unsubstituted, 5-methyl, 5-iodo-, and 7-chloro-substituted isatins **2a**–**d** (Scheme 1, Figure 1, Table 2).

In most cases, the reaction of *diexo*-β-amino amide **1a** and **1c** with isatins **2a**–**d** was selective and resulted in only a single diastereomer of spiroquinazolinone derivative **3a**–**d** and **3i**–**k** in low to moderate yields. The only exception is 7-chloroisatin **2d**, where the minor diastereomer of **3l** was also formed. (Figure 1). The reaction of *diendo*-β-amino amide **1b** and **1d** with isatin derivatives **2a**–**d** was less diastereoselective, resulting in the formation of only one diastereomer of spiroquinazolinone derivative **3e**–**g**, **3m**. In contrast, a mixture of two diastereomers of spiro derivatives 3h* and 3n*–p* were isolated in varied ratios (Figure 1).

Starting from *diexo*-β-amino amide **1a**, the reaction with isatin **2a** and 5-methylisatin **2b** afforded the best yields of 42% (**3a**) and 46% (**3b**) (Table 2 entries 1, 2). Starting from methyl-substituted *diexo*-β-amino amide **1c**, we could obtain compounds **3i** and **3j** in a 42 and 45% yield under reflux (Table 2 entries 9, 10) similar to **3a** and **3b**. It was interesting to note that I_2_ was a more effective catalyst than alum in EtOH in the synthesis of *diendo*-spiro compounds **3e**, **3g**, and **3h** (Table 2 entries 5, 7, and 8). Reactions were not completed after a long period of time using alum in EtOH under reflux. While we could obtain compounds **3o** and **3p** utilizing NH_4_Cl as catalyst, the reaction in 2M2B was not completed either.

**Figure 2:**
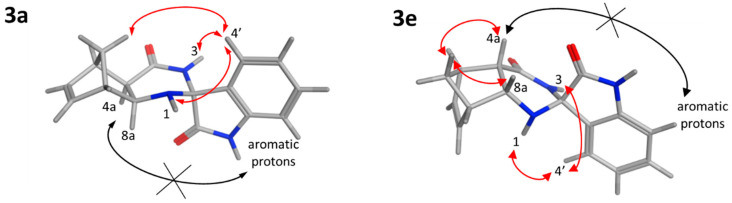
NOE interactions proving the relative configuration of **3a** and **3e**. Red arrows show the detected NOESY cross-peaks; black crossed arrows represent missing NOE contacts.

The relative configuration of the newly built stereocenter of **3a** and **3e** was determined by 2D NMR spectroscopy. For **3a**, medium-intensity NOE interactions were detected between the H4′ proton (7.29 ppm) and the H1 amine (3.54 ppm) and between H4′ and the H3 amide (8.35 ppm). In contrast, NOE signals could not be observed between the indoline protons (aromatic signals) and H4a or H8a. This indicates that the six-membered ring of indoline is located far away (>5 Å) from these hydrogens and pyrrolidone is positioned in the proximity of H4a and H8a (Figure 2). A weak NOESY cross-peak between H4′ and one of the methylene protons (1.82 ppm) provided further support that the aromatic hydrogens are closer to the methylene bridge. In line with the *diexo* configuration, NOEs were not detected between the methylene bridge and the H4a and H8a protons of norbornene. For **3e**, strong NOEs between one of the methylene bridge protons (1.41 ppm) and H4a (2.81 ppm) and H8a (4.05 ppm), respectively, supported the *diendo* arrangement. The absence of NOE cross-peaks between the aromatic protons of the indoline ring and the H4a and H8a protons indicated that they are not in close proximity. Furthermore, NOESY cross-peaks between the methylene and the aromatic hydrogens were not observable, justifying the relative configuration shown in Figure 1. Medium-intensity H4′–H1 (7.14 ppm and 2.38 ppm) and H4′–H3 (7.14 ppm and 8.18 ppm) NOE contacts were also detected.

We attempted our investigations under CF and HSBM and MW irradiation in order to make the spirocondensation reaction more sustainable and environmentally benign. First, the synthesis of **3a** was carried out (Table 3). Furthermore, we studied the synthesis of the other products **3b**–**d** and **3i**–**l** under greener conditions (Table 3). Under MW irradiation, we could obtain *diexo* spiro products **3b**–**d** and **3i**–**l** in good to excellent yields of 60–85% (Table 3). Under HSBM conditions, **3k** and **3l** did not react (Table 3 entries 12, 13). Under the CF condition, *diexo* **3a**, **3b**, and **3d** as well as *diendo* 3n* and 3p* were synthesized in good yields. The synthesis of **3c** was unsuccessful because 5-iodoisatin was insoluble in EtOH. Since the pressure increased during the synthesis, the pump had to be stopped.

**Scheme 2:**
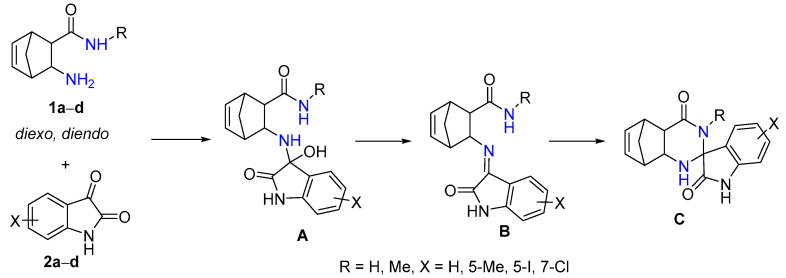
The spirocondensation reaction pathway.

In the spirocyclization reaction of **1a**–**d** with isatin derivatives **2a**–**d**, first, unstable intermediate A is produced [32]. Then, it undergoes a water elimination reaction to give imine B, which is formed by a ring-closure reaction yielding epimers of spiroquinazolinone derivative C (Scheme 2). In a few cases, intermediate B, which is the chain tautomer of spiroquinazoline C, could easily be obtained in good yield in the CF reaction.

### 2.1. Docking Studies

A challenging global health problem is the new coronavirus, which is responsible for coronavirus disease 2019 (COVID-19) [55]. Another demanding task for drug design and development is the inhibition of tryptase, which has promising therapeutic features for treating allergic or inflammatory disorders, for example, asthma and inflammatory bowel disease [56]. Hence, considering the structural similarities of their ligands with the **3a**–**g**, **3i**–**k**, and **3m** molecules, we have selected the following macromolecules for investigating the potential inhibitory activity of spiroquinazolinones:

(i) A main protease (Mpro) of SARS-CoV-2, which is a key enzyme of coronaviruses and a mediator of viral replication and transcription. Our first assumption was that the indole ring of our molecules might fit into the binding pocket of the protease where the 2-oxopyrrolidine moiety of the N3 inhibitor binds in the complex structure 6LU7 [55];

(ii) A protease tryptase, which has a significant role in mediating mast cell-dependent allergic and inflammatory responses and making it an attractive drug target [56]. Spirocyclic piperidine derivatives are potent tryptase inhibitors; a benzofurane analog was also crystallized with the protein. We assumed that spiro-quinazolinones **3a**–**p** can mimic their binding mode.

The docking studies of spiro[5,8-methanoquinazoline-2,3′-indoline]-2′,4-dione derivatives were carried out in silico using SARS-CoV-2 main protease (PDB: 6LU7) and human mast cell tryptase (PDB: 2ZA5) as targets. First, to validate our docking method, the protein’s co-crystallized ligands were redocked to the macromolecules. The docking accuracy requirement of 1.0–3.0 Å root mean square deviation (rmsd) between docked and X-ray poses is recommended for a well-docked structure [57]. We found an rmsd of less than 2 Å (Figures S1 and S2), which is regarded as satisfactory [57,58].

Then, we docked our **3a**–**g**, **3i**–**k**, and **3m** molecules to the macromolecules. Where the spirocondensation reaction was not selective and resulted in mixtures of diastereomers (3h*, 3l*, and 3n*–p*), docking studies were not performed.

In the case of the SARS-CoV-2 main protease, most of our molecules preferred the same binding pocket (Supplementary Information, Figure S1). Based on the comparison of estimated total energy and binding affinity of ligands **3a**–**3p** to the SARS-CoV-2 main protease (PDB: 6LU7), compounds **3b, 3c**, **3d**, and **3e** could be promising according to in silico results (Table 4). By visualization of the docking results, we could see the H-bond and van der Waals interactions between molecules **3a**–**g**, **3i**–**k**, and **3m** and the macromolecule (6LU7) on a 2D diagram. Molecular interactions and binding poses with the SARS-CoV-2 main protease for compounds are included in the (Supplementary Information, Figures S3–S10).

**Figure 3:**
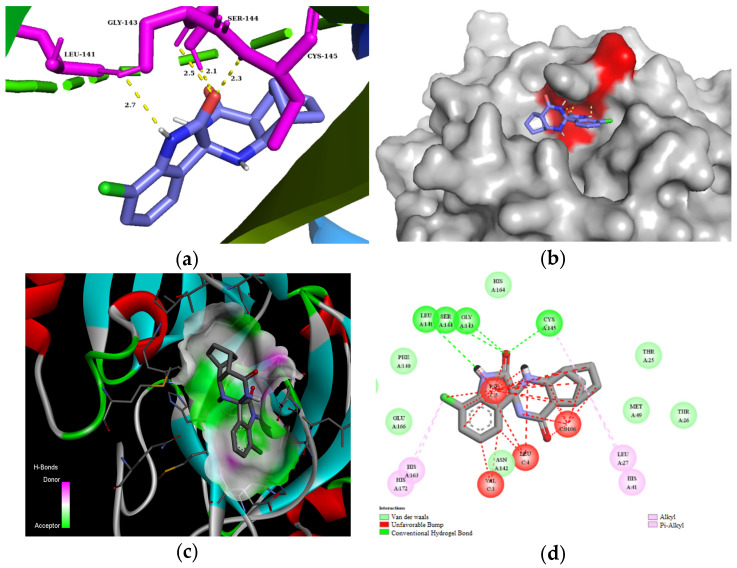
(**a**) Molecular interactions and binding pose of compound **3d** at the interface of SARS-CoV-2 main protease (PDB: 6LU7); H bonds between the macromolecule and compound **3d** are shown as yellow dashes and distances are in Å units; (**b**) Connelly surface of docking pose of 6LU7 with **3d** shown as stick model; (**c**) receptor surface: H-bond donor vs. acceptor ability of surrounding amino acids; (**d**) 2D interaction map between 6LU7 and **3d**.

**Figure 4:**
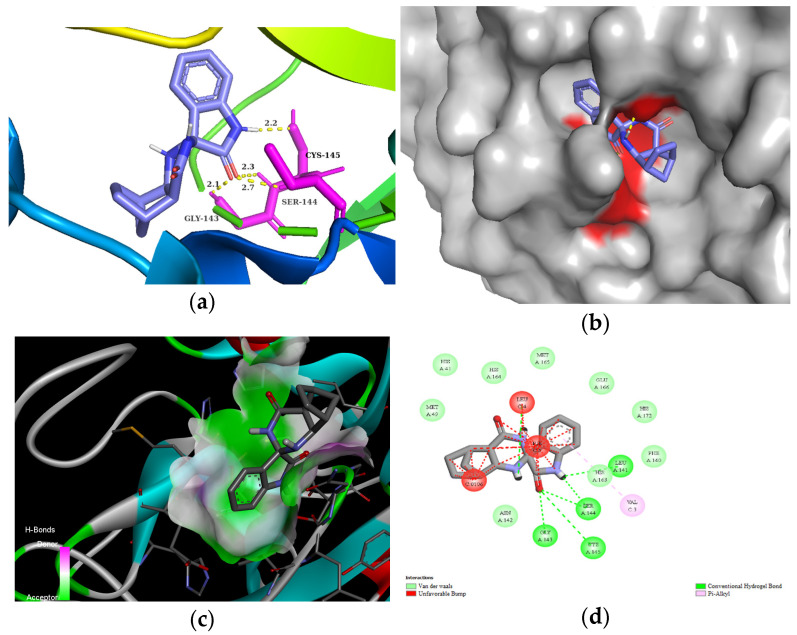
(**a**) Molecular interactions and binding pose of compound **3e** at the interface of SARS-CoV-2 main protease (PDB: 6LU7); H bonds between the macromolecule and compound **3e** are shown as yellow dashes and distances are in Å units; (**b**) Connelly surface of docking pose of 6LU7 with **3e** shown as stick model; (**c**) receptor surface: H-bond donor vs. acceptor ability of surrounding amino acids; (**d**) 2D interaction map between 6LU7 and **3e**.

The residues LEU141, GLY143, CYS145, HIS163, and HIS164 at the chain A interface of the SARS-CoV-2 main protease are known to interact with most of its ligands and are critical for binding. Molecule **3d** established H bonds with GLY143 in 2.5 Å, SER 144 in 2.1 Å, and CYS145 in 2.3 Å through its carbonyl group of the isatin ring, as well as with LEU141 in 2.7 Å with the NH group of the isatin ring (Figure 3). Moreover, compound **3d** could also create van der Waals interactions with chain A residues THR25, THR26, HIS41, PHE140, ASN142, HIS164, and GLU166, and alkyl interaction with the chloro substituent of the isatin ring with HIS163 and HIS172. On the other hand, **3d** has unfavorable interaction with the residues of chain C, e.g., PHE, LEU, and VAL. The highest binding affinity was revealed for the *diendo* compound **3e**. By visualization, it turned out that it could fit into a special binding pocket (Figure 4). Compound **3e** can form H bonds through its carbonyl group of the isatin ring with SER144 and GLY143 in 2.3 Å, 2.7 Å, and 2.1 Å. It can also establish a H bond with CYS145 through the NH group of the isatin ring.

**Figure 5:**
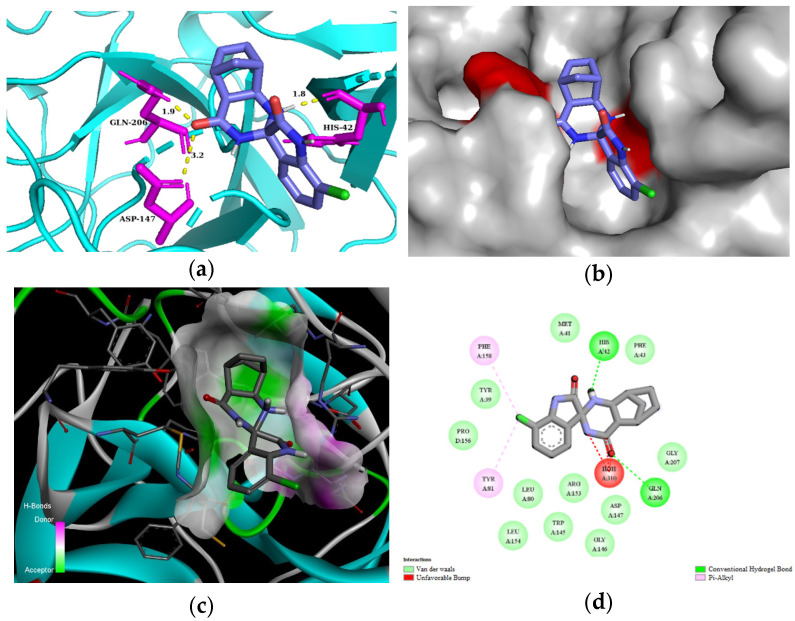
(**a**) Molecular interactions and binding pose of compound **3d** at the interface of human tryptase with potent non-peptide inhibitor (PDB: 2ZA5); H bonds between the macromolecule and compound **3d** are shown as yellow dashes and distances are in Å units; (**b**) Connelly surface of docking pose of 2ZA5 with **3d** shown as stick model; (**c**) receptor surface: H-bond donor vs. acceptor ability of surrounding amino acids; (**d**) 2D interaction map between 2ZA5 and **3d**.

For the in silico studies of human mast cell tryptase, we used the 2ZA5 PDB structure [56]. This X-ray structure of human mast cell tryptase is a tetramer containing hydrophobic binding pockets in the monomeric chains A and B. We used chain A of 2ZA5 for the studies. All of our spiroquinazolinones (**3a**–**g**, **3i**–**k**, **3m**) bound to the same binding site of human mast cell tryptase (Supplementary Information, Figure S2). Based on the comparison of the estimated total energy and binding affinity of ligands **3a**–**g**, **3i**–**k**, and **3m** to the human mast cell tryptase (PDB: 2ZA5), compounds **3a**, **3b**, **3d,** and **3f** seem promising candidates for inhibiting the enzyme (Table 5). Compound **3d** could interact with residues of the 2ZA5 macromolecule of chain A through H-bond interactions with ASP147 2.4 Å in 3 Å, excellent with HIS42 at 1.8 Å, and very well with GLN206 at a 1.9 Å distance. In addition, several van der Waals contacts were formed with the residues of 2ZA5, e.g., TRP145, GLY146, ARG153, and LEU154 (Figure 5). Molecular interactions and binding poses with mast cell tryptase for all compounds are included in the Supplementary Information (Supplementary Information, Figures S11–S18).

Summarizing the in silico results, molecules **3b**, **3d**, and **3f** proved to be promising ligands for targeting the SARS-CoV-2 main protease and human mast cell tryptase. The main driving force of the binding between the SARS-CoV-2 main protease and **3b**, **3d**, **3e**, and **3f** was hydrophobic interaction (Supplementary Information, Figure S19). On the contrary, the driving force of binding between tryptase and compounds **3a**, **3b**, **3d**, and **3f** included both hydrophobic and hydrophilic interactions (Supplementary Information, Figure S20). We speculated that the other reason can be connected to the methyl substituent of **3b** and **3f** and to the chlorine atom of **3d**. The methyl group established alkyl interactions, e.g., in the case of 6LU7, whereas the methyl group of **3b** formed an alkyl interaction with VAL104. Our best-fitting ligands were **3b**, **3d**, and **3f**, which could form interactions with residues in hydrophobic as well as hydrophilic environments. We suggested that compound **3d** is the most compelling ligand from **3a**–**g**, **3i**–**k**, and **3m** because the chlorine atom has high electronegativity and can establish H bonds and electrostatic interaction with amino acid residues. In addition, **3d** had the highest binding affinity values and high estimated total energy values concerning both macromolecules. The results of the present work could give an understanding of the inhibitory mechanism of spiroquinazolinone derivatives on tryptase and the SARS-CoV-2 main protease, and they may assist in developing novel compounds with biological activities.

### 2.2. Prediction of Pharmacokinetic Properties by SwissADME

The pharmacokinetic properties were obtained by using the SwissADME web tool in order to forecast the absorption study (water solubility), distribution study (gastrointestinal absorption and BBB permeability), and metabolism study (CYP2C9 inhibitor, CYP3A4 inhibitor, CYP2D6 substrate, CYP1A2 inhibitor, and CYP2C19 inhibitor) (Supplementary Information, Table S1) similar to that in the literature [54]. The lipophilicity of compounds **3b**, **3d**, and **3f** were in the range of 1.78 to 2.12 (recommended < 5) [61], and the logSw values were between −4.07 and −4.29, which indicated moderate water solubility and a rather lipophilic nature of the above-mentioned compounds. The data showed that the tested compounds are not able to cross the BBB. The predicted human intestinal absorption of compounds **3b**, **3d**, and **3f** was high, indicating good absorption. Moreover, compounds **3b**, **3d**, and **3f** presented good affinity toward the metabolizing enzyme CYP2D6 inhibitor. The absorption of the compounds is connected to skin permeation (logKp) and Caco-2 permeability. The skin permeation of **3b**, **3d**, and **3f** was below −7.5, which is less than −2.5, suggesting that the compounds can penetrate well into the skin. Our most promising novel compounds **3b**, **3d**, and **3f** passed the Lipinski and Veber parameters to be orally active (Supplementary Information, Table S2).

## 3. Materials and Methods

### 3.1. Experimental Procedures

General methods

All reactions were conducted using magnetic stirring in oven-dried glassware. To purify the products using column chromatography, Kieselgel 60 (Merck KGaA Darmstadt, Germany, 0.063–0.200 mm 70–230 mesh ASTM) was employed. Thin-layer chromatography was carried out on silica-coated aluminum backing plates 60A F254, using a UV lamp to see the spots (254 nm). Sulfuric acid and vanillin were utilized for detection.

Microwave-promoted reactions were carried out in sealed reaction vials (10 mL) in the cavity of a microwave equipment (CEM, Discover, SP, CEM Corporation, Matthwes, NC, USA).

All high-speed ball milling measurements were carried out with Retsch 400 Mixer Mill with 10 mL agate jars and 5 mm agate balls (Retsch Gmbh, Haan, Germany).

Ethyl acetate (EtOAc), *n*-hexane, chloroform, acetone, ethanol, and diethyl ether (Et_2_O) were among the solvents of the highest analytical grade purchased from Molar Chemicals Kft (Halásztelek, Hungary).

HPLC grade *i*-PrOH, *n*-hexane, and ethyl acetate were purchased from VWR Chemicals (Fontenary-sous-Bois, France). Isatin and alum (KAl(SO_4_)_2_·12H_2_O) were bought from Merck KGaA (Sigma Aldrich Chemie Gmbh, Steinheim, Germany). Compounds **3a**–**p** were purified with a Teledyne ISCO Combi Flash Nextgen300+ flash chromatograph (Teledyne ISCO, Lincoln, NE, USA). Purifying methods on the flash chromatograph were optimized casewise.

^1^H NMR spectra were recorded at 500.20 MHz, while ^13^C NMR spectra were measured at 125.62 MHz in CDCl_3_ or in DMSO-d_6_ at ambient temperature with a Bruker Avance NEO Ascend 500 spectrometer (Bruker Biospin, Karlsruhe, Germany) with a 5 mm Prodigy BBO probe. Chemical shifts are given, relative to tetramethylsilane (Me_4_Si) as an internal standard, in δ (ppm). For signal assignment and for determining the relative configuration, 2D COSY, HSQC, HMBC, and NOESY measurements were carried out. Some 2D spectra were recorded on a Bruker Avance 600 MHz spectrometer (Bruker Biospin, Karlsruhe, Germany) equipped with a 5 mm z-gradient CP-TCI triple-resonance cryoprobe operating at 600.2 MHz and 150.92 MHz for ^1^H and ^13^C, respectively.

The HRMS flow injection analysis was performed with a Thermo Scientific Q Exactive Plus hybrid quadrupole-Orbitrap (Thermo Fisher Scientific, Waltham, MA, USA) mass spectrometer coupled to a Waters Acquity I-Class UPLC™ (Waters, Manchester, UK). MS measurements were performed with a Thermo Fisher LTQ XL instrument using UltraZoom scan rate.

Melting points were determined with a Hinotex-X4 (Hinotek, Ningbo, China) and Kofler melting point apparatus and are uncorrected.

The synthesis of *diexo* and *diendo* 3-aminobicyclo[2.2.1]hept-5-ene-2-carboxamide **1a**,**b** were performed according to the literature [53].The syntheses of *diexo* and *diendo* 3-amino-*N*-methylbicyclo[2.2.1]hept-5-ene-2-carboxamide **1c**,**d** were performed according to the literature [54].

### 3.2. General Procedure for the Synthesis of Spiro[5,8-methanoquinazoline-2,3′-indoline]-2′,4(3H)-dione Derivatives ***3a***–***p***

Conventional method: A mixture of the corresponding β-amino amide **1a**–**d** (0.13 mmol), isatin **2a**–**d** (0.13 mmol), and the corresponding catalyst (NH_4_Cl, LiOH, *p*-TsOH, Amberlyst 15, I_2_, alum (30 mol%)) in an appropriate solvent (5 mL) was stirred under reflux or at room temperature for the specified time.

Continuous flow condition: For each reaction, the corresponding β-amino amide (c = 0.13 mmol, 1 equiv.) and isatin (0.13 mmol, 1 equiv.) were dissolved in ethanol, and the solution was pumped continuously under the following conditions: 0.1 mL min^−1^ flow rate (residence time: 5 min) and 100 °C. In each run, 4 mL of product solution was collected under steady-state conditions. Between two experiments, the system was washed for 20 min by pumping ethanol at a flow rate of 0.5 mL min^−1^. At the completion of the reaction, the product crystallized out from the solution after standing at room temperature. To carry out the synthesis of spiro[5,8-methanoquinazoline-2,3′-indoline]-2′,4-dione derivatives under continuous flow conditions, a simple flow set-up was assembled. The system contains an HPLC pump (JASCO PU-2085), a stainless-steel HPLC column with internal dimensions of 4.6 × 100 mm as a catalyst bed, which encompassed approximately 1.5 g of alum and a 100 psi backpressure regulator (BPR) from IDEX to prevent solvent boil over.

Microwave irradiation: A mixture of the corresponding isatin (0.13 mmol), β-amino amide (0.13 mmol), alum (30 mol%), and ethanol (4 mL) was stirred for the specified time at 100 °C.

High-speed ball milling (HSBM) condition: A mixture of the corresponding isatin (0.13 mmol), β-amino amide (0.13 mmol), and alum (30 mol%) was milled solvent-free for the specified time, using 3 agate balls at a 15 Hz frequency. After completion of the reaction (monitored by TLC), the crude mixture was purified by normal phase flash chromatography on a silica gel column as the stationary phase in mixtures of n-hexane and ethyl acetate with different ratios (1:1, 1:2, 2:1) as the mobile phase utilizing a 8–13 mL/min flow rate. After solvent evaporation, the pure fractions were collected and crystallized from diethyl ether.

### 3.3. NMR Spectra

#### 3.3.1. (2R*,4aR*,5R*,8S*,8aS*)-4a,5,8,8a-tetrahydro-1*H*-spiro[5,8-methanoquinazoline-2,3′-indoline]-2′,4(3*H*)-dione (**3a**)

Yield (15 mg, 42%), white solid, mp 262–268 °C (decomposition), ^1^H-NMR δH (500.20 MHz, DMSO-d_6_, 30 °C, Me_4_Si) 1.24 (1H, d, *J* = 8.3 Hz, 9-H), 1.82 (1H, d, *J* = 8.4 Hz, 9-H), 2.12 (1H, d, *J* = 6.9 Hz, 4a-H), 2.55 (1H, s, 8-H), 3.15 (1H, s, 5-H), 3.37 (1H, t, *J* = 6.2 Hz, 8a-H), 3.47 (1H, d, *J* = 5.3 Hz, 1-NH), 6.08 (1H, dd, *J* = 2.9, 5.3 Hz, 7-H), 6.29 (1H, dd, *J* = 2.9, 5.3 Hz, 6-H), 6.80 (1H, d, *J* = 7.7 Hz, Ar), 6.99 (1H, t, *J* = 7.5 Hz, Ar), 7.22–7.31 (2H, m, Ar), 8.29 (1H, s, 3-NH), 10.23 (1H, s, 1′-NH). ^13^C-NMR δC (125.62 MHz, DMSO-d_6_, 30 °C, Me_4_Si) 43.69, 44.07, 44.93, 48.31, 52.22, 71.01, 110.15, 122.24, 125.46, 129.35, 130.59, 135.39, 138.86, 142.53, 172.95, 177.05. HRMS (ESI) [M + H]^+^ *m*/*z* calcd. for C_16_H_15_N_3_O_2_: 282.11643, found: 282.12376, HPLC-MS: 282.17.

#### 3.3.2. (2R*,4aR*,5R*,8S*,8aS*)-5′-methyl-4a,5,8,8a-tetrahydro-1*H*-spiro[5,8-methanoquinazoline-2,3′-indoline]-2′,4(3*H*)-dione (**3b**)

Yield (18 mg, 46%), beige solid, mp = 318–322 °C (decomposition). ^1^H-NMR δH (500.20 MHz, DMSO-d_6_, 30 °C, Me_4_Si) 1.27 (1H, d, *J* = 8.5 Hz, 9-H), 1.81 (1H, d, *J* = 8.4 Hz, 9-H), 2.10 (1H, d, *J* = 7.8 Hz, 4a-H), 2.26 (3H, s, CH_3_), 2.54 (1H, s, 8-H), 3.15 (1H, s, 5-H), 3.37 (1H, t, *J* = 6.2 Hz, 8a-H), 3.49 (1H, d, *J* = 5.4 Hz, 1-NH), 6.03–6.13 (1H, m, 7-H), 6.26–6.32 (1H, m, 6-H), 6.69 (1H, d, *J* = 7.8 Hz, Ar), 7.07 (1H, d, *J* = 7.9 Hz, Ar), 7.12 (1H, s, Ar), 8.32 (1H, s, 3-NH), 10.22 (1H, s, 1′-NH). ^13^C-NMR δC (125.62 MHz, DMSO-d_6_, 30 °C, Me_4_Si) 21.01, 43.62, 44.05, 44.89, 48.25, 52.23, 71.04, 109.95, 126.05, 129.29, 130.77, 131.20, 135.42, 138.83, 139.05, 173.04, 177.09. HRMS (ESI) [M + H]^+^ *m*/*z* calcd. for C_17_H_17_N_3_O_2_: 296.13935, found: 296.13802, HPLC-MS: 296.22.

#### 3.3.3. (2R*,4aR*,5R*,8S*,8aS*)-5′-iodo-4a,5,8,8a-tetrahydro-1*H*-spiro[5,8-methanoquinazoline-2,3′-indoline]-2′,4(3*H*)-dione (**3c**)

Yield (21 mg, 40%), beige solid, mp 290–302 °C (decomposition). ^1^H-NMR δH (500.20 MHz, DMSO-d_6_, 30 °C, Me_4_Si) 1.28 (1H, d, *J* = 8.8 Hz, 9-H), 1.83 (1H, d, *J* = 8.6 Hz, 9-H), 2.11 (1H, d, *J* = 7.3 Hz, 4a-H), 2.57 (1H, brs, 8-H), 3.17 (1H, brs, 5-H), 3.36 (1H, t, *J* = 6.3 Hz, 8a-H), 3.60 (1H, d, *J* = 5.9 Hz, 1-NH), 6.10 (1H, dd, *J* = 2.9, 5.3 Hz, 7-H), 6.31 (1H, dd, *J* = 2.9, 5.3 Hz, 6-H), 6.68 (1H, d, *J* = 8.2 Hz, Ar), 7.58 (1H, s, Ar), 7.63 (1H, d, *J* = 8.3 Hz, Ar), 8.39 (1H, s, 3-NH), 10.43 (1H, s, 1′-NH). ^13^C-NMR δC (125.62 MHz, DMSO-d_6_, 30 °C, Me_4_Si) 43.60, 44.12, 44.91, 48.22, 52.29, 70.87, 84.81, 112.71, 131.72, 133.83, 135.45, 138.86, 139.0, 142.29, 172.79, 176.36. HRMS (ESI) [M + H]^+^ *m*/*z* calcd. for C_16_H_14_IN_3_O_2_: 408.02035, found: 408.01833, HPLC-MS: 408.13.

#### 3.3.4. (2R*,4aR*,5R*,8S*,8aS*)-7′-chloro-4a,5,8,8a-tetrahydro-1*H*-spiro[5,8-methanoquinazoline-2,3′-indoline]-2′,4(3*H*)-dione (**3d**)

Yield (14 mg, 30%), beige solid, mp 159–163 °C. ^1^H-NMR δH (500.20 MHz, DMSO-d_6_, 30 °C, Me_4_Si) 1.27 (1H, d, *J* = 7.8 Hz, 9-H), 1.81 (1H, d, *J* = 8.4 Hz, 9-H), 2.12 (1H, d, *J* = 7.0 Hz, 4a-H), 2.54 (1H, s, 8-H), 3.15 (1H, s, 5-H), 3.30-3.36 (1H, dd, *J* = 4.2, 6.4 Hz, 8a-H), 3.75 (1H, d, *J* = 5.1 Hz, 1-H), 6.09 (1H, dd, *J* = 3.0, 5.6 Hz, 7-H), 6.30 (1H, dd, *J* = 3.0, 5.6 Hz, 6-H), 7.03 (1H, t, *J* = 7.9 Hz, Ar), 7.25 (1H, d, *J* = 7.0 Hz, Ar), 7.35 (1H, d, *J* = 8.3 Hz, Ar), 8.40 (1H, s, 3-NH), 10.75 (1H, s, 1′-NH). ^13^C-NMR δC (125.62 MHz, DMSO-d_6_, 30 °C, Me_4_Si) 43.69, 44.05, 44.89, 48.37, 52.02, 71.50, 114.33, 123.68, 124.24, 130.50, 131.17, 135.31, 138.91, 140.24, 172.78, 177.08. HRMS (ESI) [M + H]^+^ *m*/*z* calcd. for C_16_H_14_ClN_3_O_2_: 316.08528, found: 316.0976, HPLC-MS: 316.11.

#### 3.3.5. (2S*,4aS*,5R*,8S*,8aR*)-4a,5,8,8a-tetrahydro-1*H*-spiro[5,8-methanoquinazoline-2,3′-indoline]-2′,4(3*H*)-dione (**3e**)

Yield (13 mg, 35%), white solid, mp 274–278 °C. ^1^H-NMR δH (500.20 MHz, DMSO-d_6_, 30 °C, Me_4_Si) 1.36–1.46 (2H, m, 9-H, 9-H), 2.39 (1H, d, *J* = 7.9 Hz, 1-NH), 2.81 (1H, dd, *J* = 8.6, 3.8 Hz, 4a-H), 2.85 (1H, s, 8-H), 3.19 (1H, s, 5-H), 4.04 (1H, ddd, *J* = 8.3, 8.3, 3.6 Hz, 8a-H), 6.22 (2H, s, 6-H, 7-H), 6.79 (1H, d, *J* = 7.7 Hz, Ar), 6.97 (1H, t, *J* = 7.5 Hz, Ar), 7.13 (1H, d, *J* = 7.3 Hz, Ar), 7.25 (1H, t, *J* = 7.7 Hz, Ar), 8.18 (1H, s, 3-NH), 10.34 (1H, s, 1′-NH).^13^C-NMR δC (125.62 MHz, DMSO-d_6_, 30 °C, Me_4_Si) 43.58, 46.07, 46.73, 46.89, 53.94, 71.36, 110.15, 122.27, 124.69, 129.40, 130.55, 134.79, 137.05, 142.14, 173.03, 176.73. HRMS (ESI) [M + H]^+^ *m*/*z* calcd. for C_16_H_15_N_3_O_2_: 282.12370, found: 282.12319, HPLC-MS: 282.15.

#### 3.3.6. (2S*,4aS*,5R*,8S*,8aR*)-5′-methyl-4a,5,8,8a-tetrahydro-1*H*-spiro[5,8-methanoquinazoline-2,3′-indoline]-2′,4(3*H*)-dione (**3f**)

Yield (14 mg, 37%), beige solid, mp 311–315 °C (decomposition). ^1^H-NMR δH (500.20 MHz, DMSO-d_6_, 30 °C, Me_4_Si) 1.38–1.45 (2H, m, 9-H, 9-H), 2.24 (3H, s, CH_3_), 2.30 (1H, d, *J* = 8 Hz, 1-NH), 2.80 (1H, dd, *J* = 8.6, 3.9 Hz, 4a-H), 2.86 (1H, s, 8-H), 3.20 (1H, s, 5-H), 4.04 (1H, ddd, J = 8.3, 8.3, 3.6 Hz, 8a-H), 6.19–6.26 (2H, m, 6-H, 7-H), 6.69 (1H, d, *J* = 7.8 Hz, Ar), 6.97 (1H, s, Ar), 7.06 (1H, d, *J* = 7.8 Hz, Ar), 8.17 (1H, s, 3-NH), 10.26 (1H, s, 1′-NH). ^13^C-NMR δC (125.62 MHz, DMSO-d_6_, 30 °C, Me_4_Si) 21.0, 43.53, 46.05, 46.77, 46.84, 53.99, 71.46, 109.96, 125.21, 129.33, 130.71, 131.26, 134.77, 137.14, 139.57, 173.15, 176.67. HRMS (ESI) [M + H]^+^ *m*/*z* calcd. for C_17_H_17_N_3_O_2_: 296.13990, found: 296.1643, HPLC-MS: 296.25.

#### 3.3.7. (2S*,4aS*,5R*,8S*,8aR*)-5′-iodo-4a,5,8,8a-tetrahydro-1*H*-spiro[5,8-methanoquinazoline-2,3′-indoline]-2′,4(3*H*)-dione (**3g**)

Yield (16 mg, 30%), beige solid, mp 275–278 °C. ^1^H-NMR δH (500.20 MHz, DMSO-d_6_, 30 °C, Me_4_Si) 1.36–1.43 (2H, m, 9-H, 9-H), 2.64 (1H, d, *J* = 7.7 Hz, 1-NH), 2.80 (1H, dd, *J* = 8.6, 3.9 Hz, 4a-H), 2.84 (1H, s, 8-H), 3.18 (1H, s, 5-H), 4.02 (1H, ddd, *J* = 8.2, 8.2, 3.5 Hz, 8a-H), 6.17–6.20 (1H, m, 6-H), 6.22–6.26 (1H, m, 7-H), 6.64 (1H, d, *J* = 8.1 Hz, Ar), 7.39 (1H, d, *J* = 1.4 Hz, Ar), 7.60 (1H, dd, *J* = 8.2, 1.4 Hz, Ar), 8.20 (1H, s, 3-NH), 10.47 (1H, s, 1′-NH). ^13^C-NMR δC (125.62 MHz, DMSO-d_6_, 30 °C, Me_4_Si) 43.62, 45.96, 46.65, 46.91, 53.89, 71.20, 84.86, 112.67, 131.68, 133.22, 135.02, 136.70, 138.98, 142.00, 172.94, 176.21. HRMS (ESI) [M + H]^+^ *m*/*z* calcd. for C_16_H_14_IN_3_O_2_: 408.02035, found: 408.01997, HPLC-MS: 408.14.

#### 3.3.8. (4aS*,5R*,8S*,8aR*)-7′-chloro-4a,5,8,8a-tetrahydro-1*H*-spiro[5,8-methanoquinazoline-2,3′-indoline]-2′,4(3*H*)-dione (3h*)

Mixtures of diastereomers of 2R* and 2S*, yield (12 mg, 28%), yellow/cream solid, mp 212-217 (decomposition) °C. ^1^H-NMR δH (500.20 MHz, DMSO-d_6_, 30 °C, Me_4_Si) 1.38–1.45 (2H, m, 9-H, 9-H), 2.64 (1H, d, *J* = 7.2 Hz, 1-NH), 2.82–2.86 (2H, m, 4a-H, 8-H), 3.19 (1H, brs, 5-H), 4.01-4.06 (1H, m, 8a-H), 6.18-6.21 (2H, m, 6-H, 7-H), 7.00 (1H, t, *J* = 7.6 Hz, Ar), 7.10 (1H, d, *J* = 7.1 Hz, Ar), 7.32 (1H, d, *J* = 8.2 Hz, Ar), 8.16 (1H, s, 3-NH), 10.74 (1H, s, 1′-NH). ^13^C-NMR δC (125.62 MHz, DMSO-d_6_, 30 °C, Me_4_Si) 43.51, 45.97, 46.76, 46.84, 53.78, 71.88, 114.47, 123.34, 124.00, 130.60, 136.89, 138.07, 139.66, 173.84, 176.61. HRMS (ESI) [M + H^+^]^+^ *m*/*z* calcd. for C_16_H_14_ClN_3_O_2_: 316.08528, found: 316.0717.

#### 3.3.9. (2R*,4aR*,5R*,8S*,8aS*)-3-methyl-4a,5,8,8a-tetrahydro-1*H*-spiro[5,8-methanoquinazoline-2,3′-indoline]-2′,4(3*H*)-dione (**3i**)

Yield (15 mg, 46%), white solid, mp 271–274 °C. ^1^H-NMR δH (500.20 MHz, DMSO-d_6_, 30 °C, Me_4_Si) 1.26 (1H, d, *J* = 8.5 Hz, 9-H), 1.87 (1H, d, *J* = 8.5 Hz, 9-H), 2.17 (1H, d, *J* = 7.0 Hz, 4a-H), 2.42 (3H, s, CH_3_), 2.57 (1H, s, 5-H), 3.18 (1H, s, 1-H), 3.30–3.35 (1H, m, 8a-H), 3.81 (1H, d, *J* = 4.8 Hz, 1-NH), 6.06–6.09 (1H, m, 7-H), 6.31–6.34 (1H, m, 6-H), 6.88 (1H, d, *J* = 7.7 Hz, Ar), 7.05 (1H, t, *J* = 7.5 Hz, Ar), 7.28–7.34 (2H, m, Ar), 10.54 (1H, s, 1′-NH). ^13^C-NMR δC (125.62 MHz, DMSO-d_6_, 30 °C, Me_4_Si) 29.9, 43.99, 44.57, 45.44, 48.46, 51.43, 75.84, 110.83, 122.60, 125.61, 127.78, 130.95, 135.20, 139.16, 142.41, 172.19, 176.11. HRMS (ESI) [M + H]^+^ *m*/*z* calcd. for C_17_H_17_N_3_O_2_: 296.13935, found: 296.13891, HPLC-MS: 296.20.

#### 3.3.10. (2R*,4aR*,5R*,8S*,8aS*)-3,5′-dimethyl-4a,5,8,8a-tetrahydro-1*H*-spiro[5,8-methanoquinazoline-2,3′-indoline]-2′,4(3*H*)-dione (**3j**)

Yield (15 mg, 42%), beige solid, mp 221–226 °C (decomposition). ^1^H-NMR δH (500.20 MHz, DMSO-d_6_, 30 °C, Me_4_Si) 1.27 (1H, d, *J* = 8.4 Hz, 9-H), 1.87 (1H, d, *J* = 8.5 Hz, 9-H), 2.16 (1H, d, *J* = 7.0 Hz, 4a-H), 2.28 (3H, s, CH_3_), 2.42 (3H, s, HN-CH_3_), 2.57 (1H, s, 5-H), 3.18 (1H, s, 8-H), 3.30–3.38 (1H, m, 8a-H), 3.77 (1H, d, *J* = 4.8 Hz, 1-NH), 6.05–6.09 (1H, m, 6-H), 6.30–6.34 (1H, m, 7-H), 6.77 (1H, d, *J* = 8.1 Hz, Ar), 7.10–7.15 (2H, m, Ar), 10.44 (1H, s, 1′-NH). ^13^C-NMR δC (125.62 MHz, DMSO-d_6_, 30 °C, Me_4_Si) 21.03, 29.93, 43.99, 44.56, 45.43, 48.46, 51.48, 75.92, 110.57, 126.07, 127.80, 131.13, 131.66, 135.20, 139.14, 139.88, 172.21, 176.12. HRMS (ESI) [M + H]^+^ *m*/*z* calcd. for C_18_H_19_N_3_O_2_: 310.15500, found: 310.15458, HPLC-MS: 310.20.

#### 3.3.11. (2R*,4aR*,5R*,8S*,8aS*)-5′-iodo-3-methyl-4a,5,8,8a-tetrahydro-1*H*-spiro[5,8-methanoquinazoline-2,3′-indoline]-2′,4(3*H*)-dione (**3k**)

Yield (13 mg, 24%), ecru solid, mp 237–241 °C. ^1^H-NMR δH (500.20 MHz, DMSO-d_6_, 30 °C, Me_4_Si) 1.27 (1H, d, *J* = 8.0 Hz, 9-H), 1.88 (1H, d, *J* = 8.6 Hz, 9-H), 2.15 (1H, d, *J* = 6.9 Hz, 4a-H), 2.43 (3H, s, CH_3_), 2.58 (1H, s, 5-H), 3.18 (1H, s, 8-H), 3.30–3.32 (1H, m, 8a-H), 3.86 (1H, s, 1-NH), 6.07–6.10 (1H, m, 7-H), 6.31–6.34 (1H, m, 6-H), 6.74 (1H, d, *J* = 8.2 Hz, Ar), 7.60–7.62 (1H, m, Ar), 7.66 (1H, dd, *J* = 8.2, 1.8 Hz, Ar), 10.68 (1H, s, NH). ^13^C-NMR δC (125.62 MHz, DMSO-d_6_, 30 °C, Me_4_Si) 30.0, 44.05, 44.53, 45.44, 48.41, 51.52, 75.77, 85.41, 113.33, 130.12, 133.79, 135.23, 139.18, 139.44, 142.15, 172.07, 175.41. HRMS (ESI) [M + H]^+^ *m*/*z* calcd. for C_17_H_16_IN_3_O_2_:422.02872, found: 422.0232, HPLC-MS: 422.22.

#### 3.3.12. (4aR*,5R*,8S*,8aS*)-7′-chloro-3-methyl-4a,5,8,8a-tetrahydro-1*H*-spiro[5,8-methanoquinazoline-2,3′-indoline]-2′,4(3*H*)-dione (3l*)

Mixture of diastereomers of 2R* and 2S*, where the major compound (2R*) can be identified at NMR spectra, yield (16 mg, 38%), ecru solid, mp 346–350 °C (decomposition). ^1^H-NMR δH (500.20 MHz, CDCl_3_, 30 °C, Me_4_Si) 1.48 (1H, d, *J* = 8.9 Hz, 9-H), 1.90 (1H, d, *J* = 9.0 Hz, 9-H), 2.53 (1H, d, *J* = 7.1 Hz), 2.64 (1H, s, 5-H), 2.66 (3H, s, CH_3_), 3.46 (1H, s, 8-H), 3.56 (1H, d, *J* = 7.2 Hz), 6.04–6.07 (1H, m, 7-H), 6.34–6.38 (1H, m, 6-H), 7.09 (1H, d, *J* = 7.9 Hz, Ar), 7.22 (1H, d, *J* = 7.4 Hz, Ar), 7.31–7.36 (1H, m, Ar), 8.45 (1H, s, NH), 8.77 (1H, s, NH) for the major compound. ^13^C-NMR δC (125.62 MHz, CDCl_3_, 30 °C, Me_4_Si) 30.46, 43.84, 44.81, 45.71, 48.70, 51.76, 76.57, 116.20, 122.99, 123.86, 124.60, 128.56, 130.86, 134.23, 138.26, 139.37, 139.53, 172.98, 174.67 for the major compound. HRMS (ESI) [M + H]^+^ *m*/*z* calcd. for C_17_H_16_ClN_3_O_2_: 330.10038, found: 330.09827, HPLC-MS: 341.38.

#### 3.3.13. (2S*,4aS*,5R*,8S*,8aR*)-3-methyl-4a,5,8,8a-tetrahydro-1*H*-spiro[5,8-methanoquinazoline-2,3′-indoline]-2′,4(3*H*)-dione (**3m**)

Yield (12 mg, 31%), white solid, mp 255–257 °C. ^1^H-NMR δH (500.20 MHz, DMSO-d_6_, 30 °C, Me_4_Si) 1.37–1.44 (2H, m, 9-H, 9-H), 2.34 (3H, s, CH_3_), 2.78 (1H, d, *J* = 7.2 Hz, 1-NH), 2.86 (1H, s, 8-H), 2.88 (1H, dd, *J* = 8.6, 3.9 Hz, 4a-H), 3.22 (1H, s, 8-H), 4.00 (1H, ddd, *J* = 8.0, 8.0, 3.6 Hz, 8a-H), 6.22 (2H, brs, 6-H, 7-H), 6.87 (1H, d, *J* = 7.7 Hz, Ar), 7.02 (1H, t, *J* = 7.5 Hz, Ar), 7.14 (1H, d, *J* = 7.3 Hz, Ar), 7.30 (1H, t, *J* = 7.7 Hz, Ar), 10.58 (1H, s, 1′-NH). ^13^C-NMR δC (125.62 MHz, DMSO-d_6_, 30 °C, Me_4_Si) 29.74, 44.23, 46.49, 46.83, 47.17, 53.23, 76.21, 110.83, 122.61, 124.79, 127.83, 130.82, 135.14, 136.61, 142.04, 172.45, 175.85. HRMS (ESI) [M + H]^+^ *m*/*z* calcd. for C_17_H_17_N_3_O_2_: 296.13990, found: 296.1610, HPLC-MS: 296.21.

#### 3.3.14. (4aS*,5R*,8S*,8aR*)-3,5′-dimethyl-4a,5,8,8a-tetrahydro-1*H*-spiro[5,8-methanoquinazoline-2,3′-indoline]-2′,4(3*H*)-dione (3n*)

Mixture of diastereomers of 2R* and 2S*, where the major compound can be identified with NMR spectra, yield (10 mg, 24%), yellow solid, mp 251–254 °C. ^1^H-NMR δH (500.20 MHz, DMSO-d_6_, 30 °C, Me_4_Si) 1.37–1.41 (2H, m, 9-H, 9-H), 2.26 (3H, s, CH_3_), 2.34 (3H, s, CH_3_), 2.72 (1H, d, *J* = 7.2 Hz, 1-NH), 2.85–2.89 (2H, m, 4a-H, 8-H), 3.23 (1H, s, 5-H), 4.01 (1H, ddd, *J* = 8.0, 8.0, 3.6 Hz, 8a-H), 6.19–6.25 (2H, m, 6-H, 7-H), 6.75 (1H, d, *J* = 7.8 Hz, Ar), 6.97 (1H, s, Ar), 7.09 (1H, d, *J* = 8.0 Hz, Ar), 10.47 (1H, s, NH) for the major compound. ^13^C-NMR δC (125.62 MHz, DMSO-d_6_, 30 °C, Me_4_Si) 21.01, 29.76, 44.21, 46.48, 46.69, 47.16, 53.27, 76.30, 110.57, 125.27, 127.85, 131.00, 131.66, 135.16, 136.66, 139.54, 172.41, 175.83 for the major compound. HRMS (ESI) [M + H]^+^ *m*/*z* calcd. for C_18_H_19_N_3_O_2_: 310.15555, found: 310.1568, HPLC-MS: 310.19.

#### 3.3.15. (4aS*,5R*,8S*,8aR*)-5′-iodo-3-methyl-4a,5,8,8a-tetrahydro-1*H*-spiro[5,8-methanoquinazoline-2,3′-indoline]-2′,4(3*H*)-dione (3o*)

Mixture of diastereomers of 2R* and 2S*, where the major compound can be identified with NMR spectra, yield (11 mg, 25%), white solid, mp 179–183 °C. ^1^H-NMR δH (500.20 MHz, DMSO-d_6_, 30 °C, Me_4_Si) 1.35–1.45 (2H, m, 9-H, 9-H), 2.34 (3H, s, CH_3_), 2.84 (1H, brs, 8H), 2.88 (1H, dd, *J* = 8.7, 4.0 Hz, 4a-H), 3.07 (1H, d, *J* = 7.2 Hz, 1-NH), 3.20 (1H, s, 5-H), 3.98 (1H, ddd, *J* = 8.0, 8.0, 3.6 Hz, 8a-H), 6.16–6.20 (1H, m, 7H), 6.23–6.27 (1H, m, 6H), 6.72 (1H, d, *J* = 8.0 Hz, Ar), 7.41 (1H, s, Ar), 7.64 (1H, d, *J* = 8.2 Hz, Ar), 10.71 (1H, s, NH) for the major compound. ^13^C-NMR δC (125.62 MHz, DMSO-d_6_, 30 °C, Me_4_Si) 29.78, 44.27, 46.41, 46.76, 47.19, 53.21, 76.08, 85.35, 113.30, 130.11, 133.15, 135.35, 136.33, 139.37, 141.89, 172.35, 175.32 for the major compound. HRMS (ESI) [M + H]^+^ *m*/*z* calcd. for C_17_H_16_IN_3_O_2_: 422.02872, found: 422.0321, HPLC-MS: 422.16.

#### 3.3.16. (4aS*,5R*,8S*,8aR*)-7′-chloro-3-methyl-4a,5,8,8a-tetrahydro-1*H*-spiro[5,8-methanoquinazoline-2,3′-indoline]-2′,4(3*H*)-dione (3p*)

Mixture of diastereomers of 2R* and 2S*, yield (12 mg, 34%), white solid, mp 178–182 °C. ^1^H-NMR δH (500.20 MHz, DMSO-d_6_, 30 °C, Me_4_Si) 1.36–1.54 (2H, m, 9-H, 9-H), 2.36 (3H, s, CH_3_), 2.39 (3H, ms, CH_3_), 2.84 (1H, s, 8-H), 2.88–2.93 (1H, m, 4a-H), 2.99 (1H, s, 8-H), 3.08 (1H, d, *J* = 6.7 Hz, 1-NH), 3.12–3.15 (1H, m, 4a-H), 3.21 (1H, m, 5-H), 3.32 (1H, m, 5-H), 6.19 (2H, brs, 6-H, 7-H), 6.27–6.30 (1H, m, 7-H), 6.39–6.42 (1H, m, 6-H), 7.02–7.07 (1H, m, Ar), 7.08–7.13 (3H, m, Ar), 7.37–7.40 (1H, m, Ar), 7.42–7.46 (1H, m, Ar), 7.47–7.52 (1H, m, Ar), 11.05 (1H, s, NH), 11.36 (1H, s, NH) for the major and minor compounds. ^13^C-NMR δC (125.62 MHz, DMSO-d_6_, 30 °C, Me_4_Si) 28.92, 29.73, 43.94, 44.29, 46.42, 46.68, 46.72, 46.87, 47.26, 47.50, 53.15, 54.29, 76.72, 77.59, 115.05, 115.46, 123.57, 123.71, 123.97, 124.28, 129.55, 129.66, 130.53, 130.78, 133.82, 135.32, 136.24, 136.26, 137.87, 139.55, 139.66, 139.88, 170.56, 172.33, 173.03, 176.01 for the major and minor compounds. HRMS (ESI) [M + H]^+^ *m*/*z* calcd. for C_17_H_16_ClN_3_O_2_: 330.10093, found: 330.1410, HPLC-MS: 330.23.

#### 3.3.17. (1R*,2R*,3S*,4S*)-3-((*Z*)-(5-methyl-2-oxoindolin-3-ylidene)amino)bicyclo[2.2.1]hept-5-ene-2-carboxamide (**Bb**)

Yield (22 mg, 58%), white solid, mp 245–248 °C ^1^H-NMR δH (600.20 MHz, DMSO-d_6_, 30 °C, Me_4_Si) 1.41 (1H, d, *J* = 8.2 Hz, 7-H), 2.26 (3H, s, CH_3_), 2.42 (1H, dd, *J* = 1.8, 7.9 Hz, 2-H), 2.44 (1H, d, *J* = 8.1 Hz, 7-H), 2.60 (1H, s, 4-H), 2.94 (1H, s, 1-H), 5.35 (1H, d, *J* = 8.0 Hz, 3-H), 6.23 (1H, dd, *J* = 3.0, 5.6 Hz, 5-H), 6.35 (1H, dd, *J* = 3.0, 5.6 Hz, 6-H), 6.57 (1H, s, NH), 6.63 (1H, s, NH), 6.73 (1H, d, *J* = 7.9 Hz, Ar), 7.16–7.19 (2H, m, Ar), 10.75 (1H, s, NH). ^13^C-NMR δC (150.92 MHz, DMSO-d_6_, 30 °C, Me_4_Si) 20.87, 44.92, 45.46, 50.53, 51.02, 61.54, 110.62, 122.36, 122.44, 131.48, 133.48, 136.76, 140.56, 142.35, 153.56, 160.43, 173.72. HRMS (ESI) [M + H]^+^ *m*/*z* calcd. for C_17_H_17_N_3_O_2_: 296.13935, found: 296.1607.

#### 3.3.18. (1R*,2R*,3S*,4S*)-3-((*Z*)-(7-chloro-2-oxoindolin-3-ylidene)amino)bicyclo[2.2.1]hept-5-ene-2-carboxamide (**Bd**)

Yield (21 mg, 47%), white solid, mp 242–245 °C ^1^H-NMR δH (600.20 MHz, DMSO-d_6_, 30 °C, Me_4_Si) 1.42 (1H, d, *J* = 8.2 Hz, 7-H), 2.42–2.45 (2H, m, 2-H, 7-H), 2.65 (1H, s, 4-H), 2.95 (1H, s, 1-H), 5.35 (1H, d, *J* = 8.0 Hz, 3-H), 6.24 (1H, dd, *J* = 3.0, 5.6 Hz, 5-H), 6.37 (1H, dd, *J* = 3.0, 5.6 Hz, 6-H), 6.56 (1H, s, NH), 6.67 (1H, s, NH), 7.03 (1H, t, *J* = 7.7 Hz, Ar), 7.34 (1H, d, *J* = 7.6 Hz, Ar), 7.46 (1H, d, *J* = 8.1 Hz, Ar), 11.32 (1H, s, NH). ^13^C-NMR δC (150.92 MHz, DMSO-d_6_, 30 °C, Me_4_Si) 44.93, 45.47, 50.74, 50.92, 61.88, 115.10, 120.65, 123.78, 124.11, 132.79, 136.68, 140.69, 142.06, 152.87, 160.15, 173.58. HRMS (ESI) [M + H]^+^ *m*/*z* calcd. for C_16_H_14_ClN_3_O_2_: 316.08528, found: 316.0976.

### 3.4. Docking Studies

For docking studies, the macromolecules SARS-CoV-2 main protease (PDB: 6LU7) and human mast cell tryptase (PDB: 2ZA5) were downloaded in pdb format from RCSB Protein Data Bank (https://www.rcsb.org) (accessed on 1 August 2024). Preparing the macromolecules for docking was performed with the AutoDock4 software. Ligand structures were optimized and minimized through Avogadro software (version 1.95.0). Docking protocols were accomplished by the PyRx-Virtual [60] screening tool and iGEMDOCK version 2.1 software [59]. Blind docking was accomplished. The docking calculation was partially flexible (rigid protein, flexible ligand). Validation of the molecular docking method was accomplished by redocking the crystallographic ligand of the target enzymes. The ligand interactions were visualized and analyzed through PyMOL 2.5 (The PyMOL Molecular Graphics System, Version 2.5 Schrödinger, LLC) and Discovery Studio Visualizer (BIOVIA, Dassault Systèmes, BIOVIA Discovery Studio, 4.5, San Diego: Dassault Systèmes, 2021).

All figures representing the binding ligand position of binding pockets in the case of both macromolecules were visualized in PyMOL 2.5 software. H-bond interactions were signed with distances in Å. The 2D interaction diagrams of the ligand interaction with the residues of the macromolecule were created utilizing BIOVIA Discovery Studiodraw software.

### 3.5. SwissADME Predictions

Information with respect to pharmacokinetic properties was collected by using the SwissADME web tool in order to forecast the absorption study (water solubility), distribution study (gastrointestinal absorption and blood–brain barrier (BBB) permeability), metabolism study, etc. [62]. The SwissADME is a free and user-friendly web application, allowing a simple assessment of pharmacokinetics [62]. BBB permeability and HIA (human intestinal absorption) represent the absorption and the distribution of compounds. The drug likeness property envisions the molecules, which could improve an orally bioavailable drugs by contrasting their physicochemical or structural characteristics [63]. The five features of the Lipinski rule state that oral drugs should not have more than one violation, which include the following: molecular weight ≤500 g/mol; partition coefficient ≤ 5; number of H-bond accepters ≤ 10 and H-bond donors ≤ 5; and the number of rotatable bonds ≤ 10 [63]. The Veber rule requires compounds to have ≤10 rotatable bonds and a polar surface area of less than 140 Å [64].

## 4. Conclusions

With the aim of helping drug design and improvement, we have synthesized novel tetrahydro-1*H*-spiro[5,8-methanoquinazoline-2,3′-indoline]-2′,4-dione derivatives. Furthermore, the synthesis of these spiro compounds was optimized by developing new methods and testing both the catalysts and solvents. The best methods were using alum as a catalyst in EtOH, I_2_ in EtOH at 78 °C, and NH_4_Cl in 2M2B at 100 °C in all cases. In order to make the synthesis route greener, different kinds of environmentally benign methods such as MW irradiation, HSBM, and CF were utilized. Of these, MW irradiation was found to be the most effective to achieve excellent yields up to 85%. Reaction times in each case were significantly reduced with the usage of a microwave reactor. In the case of HSBM conditions, we could develop a solvent-free method with moderate yields. Docking studies were performed in silico comparing two different macromolecules, namely the SARS-CoV-2 main protease (PDB: 6LU7) and human mast cell tryptase (PDB: 2ZA5). According to the estimated total energy and binding affinity, compounds **3b**, **3d**, and **3f** of our novel derivatives were found to be good fitting ligands to both macromolecules (6LU7 and 2ZA5). We also suggest **3e** as an outstanding ligand to the SARS-CoV-2 main protease (PDB: 6LU7). Thus, the present results are essential for the design, synthesis, and improvement in novel spiroquinazolinone derivatives with promising biological effects and additional modification of the structure in the future. We suggested that compound **3d** is an outstanding ligand because of the high electronegativity of the chlorine atom. It can form H bonds and electrostatic interactions with amino acid residues in hydrophobic environments. The ADMET data predictions by the SwissADME web tool theoretically predicted that the present novel tetrahydro-1*H*-spiro[5,8-methanoquinazoline-2,3′-indoline]-2′,4-dione derivatives could be promising biologically active molecules. Moreover, they could be orally active, and they could penetrate the skin according to theoretical predictions by SwissADME.

## Figures and Tables

**Table 1:** Synthesis of 4a,5,8,8a-tetrahydro-1*H*-spiro[5,8-methanoquinazoline-2,3′-indoline]-2′,4(3*H*)-dione (**3a**).

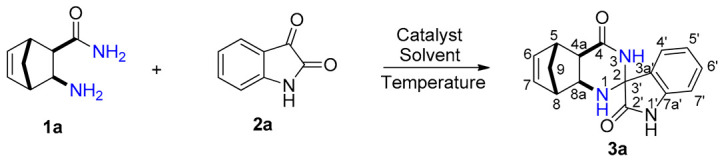					
Entry	Catalyst	Solvent	Temp. (°C)	Time (h)	Yield ^a^ (%)
1	NH_4_Cl	EtOH	Rt ^b^	24	29 ^c^
2	NH_4_Cl	EtOH	78	12	37 ^c^
3	NH_4_Cl	2M2B ^d^	100	9	35
4	LiOH	EtOH	Rt ^b^	72	10
5	LiOH	EtOH	78	72	21
6	*p*-TsOH	EtOH	Rt ^b^	168	–
7	*p*-TsOH	EtOH	78	168	–
8	Amberlyst 15	EtOH	Rt ^b^	120	20 ^c^
9	Amberlyst 15	EtOH	78	10	25 ^c^
10	I_2_	EtOH	Rt ^b^	215	–
11	I_2_	EtOH	78	14	35
12	Alum	Glycerol	Rt ^b^	168	–
13	Alum	Glycerol	100	5	–
14	Alum	2M2B ^d^	Rt ^b^	72	–
15	Alum	2M2B ^d^	100	168	–
16	Alum	EtOH	78	5	42
17	Alum	Water	rt	168	– ^e^
18	Alum	Water	100	8	– ^e^

^a^ Isolated yield after purification by flash chromatography. ^b^ Room temperature. ^c^ The reaction was not selective, since the product contained the mixture of two diastereomers according to the ^1^H-NMR spectrum. ^d^ 2M2B: 2-methyl-2-butanol. ^e^ The starting materials did not dissolve in water and there was no transformation.

**Table 2:** Synthesis of spiro[5,8-methanoquinazoline-2,3′-indoline]-2′,4-dione derivatives **3a**–**p** ^a^.

Entry	Amide	Ketone	Time (h)	Product	Yield ^b^ (%)	de ^c^ (Major/Minor)
1	**1a**	**2a**	6	**3a**	42	1:0
2	**1a**	**2b**	5	**3b**	46	1:0
3	**1a**	**2c**	6	**3c**	29	1:0
4	**1a**	**2d**	6	**3d**	30	1:0
5	**1b**	**2a**	12	**3e** ^d^	35	1:0
6	**1b**	**2b**	10	**3f**	37	1:0
7	**1b**	**2c**	24	**3g** ^d^	30	1:0
8	**1b**	**2d**	24	**3h** ^d^	28	1:0.3
9	**1c**	**2a**	5	**3i**	45	1:0
10	**1c**	**2b**	5	**3j**	42	1:0
11	**1c**	**2c**	6	**3k**	24	1:0
12	**1c**	**2d**	6	**3l**	38	1:0.4
13	**1d**	**2a**	14	**3m**	31	1:0
14	**1d**	**2b**	20	**3n**	24	1:0.4
15	**1d**	**2c**	24	**3o** ^e^	25	1:1
16	**1d**	**2d**	24	**3p** ^e^	34	1.3:1

^a^ A mixture of the corresponding isatin (0.13 mmol), β-amino amide (0.13 mmol), alum (20 mg, 30 mol%), and EtOH (5 mL) was stirred under reflux for the specified time. ^b^ Isolated yield after purification by flash chromatography. ^c^ The diastereoselectivity major/minor ratio was calculated by NMR measurements. ^d^ A mixture of the corresponding isatin (0.13 mmol), β-amino amide (0.13 mmol), I_2_ (19 mg, 30 mol%), and EtOH (5 mL) was stirred under reflux for the specified time. ^e^ A mixture of corresponding isatin (0.13 mmol), β-amino amide (0.13 mmol), NH_4_Cl (5 mg, 30 mol%), and 2M2B (5 mL) was stirred under reflux for the specified time.

**Table 3:** Synthesis of spiro[5,8-methanoquinazoline-2,3′-indoline]-2′,4-dione derivatives 3 under microwave irradiation, HSBM, and CF conditions.

Entry	Method	Product	Time (h)	Yield ^a^ (%)
1	MW ^b^	**3a**	0.5	85
2	MW ^b^	**3b**	1.5	83
3	MW ^b^	**3c**	1	85
4	MW ^b^	**3d**	2	71
5	MW ^b^	**3i**	1	70
6	MW ^b^	**3j**	1.5	65
7	MW ^b^	**3k**	2.5	59
8	MW ^b^	**3l**	2.5	60
9	HSBM ^c^	**3a**	6	45
10	HSBM ^c^	**3i**	6	45
11	HSBM ^c^	**3j**	6	37
12	HSBM ^c^	**3k**	4	-
13	HSBM ^c^	3l*	4	-
14	CF ^d^	**3a**	0.08 ^d^	70
15	CF ^d^	**3b**	0.08 ^d^	62
16	CF ^d^	**3d**	0.08 ^d^	70
17	CF ^d^	3n*	0.08 ^d^	52
18	CF ^d^	3p*	0.08 ^d^	44

^a^ Isolated yield after purification by flash chromatography. ^b^ Reaction conditions for microwave irradiation: a mixture of the corresponding isatin (0.13 mmol), β-amino amide (0.13 mmol), alum (20 mg, 30 mol%), and ethanol (4 mL) was stirred for the specified time at 100 °C. ^c^ Reaction conditions for high-speed ball milling: a mixture of the corresponding isatin (0.13 mmol), β-amino amide (0.13 mmol), and alum (20 mg, 30 mol%) was milled solvent-free for the specified time; use of 3 agate balls utilizing 15 Hz frequency. * mixtures of diastereomers. ^d^ Reaction conditions for CF reactions: 0.1 mL min^−1^ flow rate (residence time: 5 min), 100 °C, β-amino amide (c = 0.1 M, 1 equiv), and isatin derivative (1 equiv) dissolved in ethanol (4 mL).

**Table 4:** Docking results of compounds **3a–g**, **3i–k**, and **3m** with SARS-CoV-2 main protease (PDB: 6LU7) in silico.

Molecules	Estimated Total Energy (kcal/mol) ^a^	Binding Affinity (kcal/mol) ^b^	Active Site Residue Chain A ^c^
**3a**	−62.559	−7.8	LEU141, SER144, CYS145
**3b**	−63.773	−8.1	LEU141, SER144, CYS145
**3c**	−59.252	−8.2	SER144, CYS145
**3d**	−64.985	−8.1	LEU141, GLY143, SER144, CYS145
**3e**	−55.856	−8.6	GLY143, SER144, CYS145
**3f**	−58.697	−7.7	GLY143, SER144, CYS145
**3g**	−57.707	−7.7	LEU141, GLY143, SER144, CYS145
**3i**	−64.442	−7.3	SER158
**3j**	−65.518	−7.4	GLY143, HIS164
**3k**	−57.736	−7.1	GLY143, HIS164
**3m**	−56.195	−7.8	HIS144

^a^ Estimated total energy values were calculated by iGEMDOCK [59]. ^b^ Binding affinity of ligands to the macromolecule was calculated with the use of PyRx [60]. ^c^ The residues which took part in H bonding are represented from the residues of the active site: residues 141–143, 145, 164, long strand on one side of residues 155–168 and on the other side of residues 189–191 [55].

**Table 5:** Docking results of compounds **3a–g**, **3i–k**, and **3m** with human mast cell tryptase (PDB: 2ZA5) in silico.

Molecules	Estimated Total Energy (kcal/mol) ^a^	Binding Affinity (kcal/mol) ^b^	Residue Chain A ^c^
**3a**	−100.957	−8.7	HIS42, ARG153
**3b**	−100.858	−9.0	ASP147, GLN206
**3c**	−97.333	−8.6	HIS42, ASP147 ARG153, GLN206
**3d**	−100.224	−9.1	HIS42, ASP146, GLN206
**3e**	−87.828	−8.6	TRP145, GLN206
**3f**	−98.296	−8.7	HIS42, TYR81, TRP145, ASP147, GLN206
**3g**	−95.208	−8.7	HIS42, TYR81, TRP145, ASP147, GLN206
**3i**	−100.404	−8.0	HIS42, ASP147
**3j**	−100.032	−8.3	HIS42, ARG153
**3k**	−97.9363	−7.5	TYR39, TYR81, ARG153, LEU154
**3m**	−86.146	−8.6	HIS42, TYR81, TRP145, ASP147

^a^ Estimated total energy values were calculated by iGEMDOCK [59]. ^b^ Binding affinity of ligands to the macromolecule was calculated with the use of PyRx [60]. ^c^ The residues of monomer A which took part in H bonding are represented.

## Data Availability

Data are contained within the article or Supplementary Material.

## Supplementary Materials

The following supporting information can be downloaded at: https://www.mdpi.com/article/10.3390/molecules29215112/s1, Figure S1: Docking accuracy validation and binding poses of co-ligand of 6LU7 and the redocked one of SARS-CoV-2 main protease (PDB: 6LU7) and binding poses of molecules **3a**–**g**, **3i**–**k** and **3m** with 6LU7; Figure S2: Docking accuracy validation and binding poses of co-ligand of 2ZA5 and the redocked one human tryptase with potent non-peptide inhibitor (PDB: 2ZA5) and binding poses of molecules **3a**–**g**, **3i**–**k** and **3m** with 2ZA5; Figures S3–S10: Molecular interactions and binding poses and H bonds and connelly surface of docked pose of compound **3a**, **3c**, **3e**, **3g**, **3i**, **3j**, **3k** and **3m** at the interface of SARS-CoV-2 main protease (PDB: 6LU7); Figures S11–S18: Molecular interactions and binding poses and H bonds and connelly surface of docked pose of compound **3a**, **3c**, **3e**, **3g**, **3i**, **3j**, **3k** and **3m** at the interface of SARS-CoV-2 main protease (PDB: 6LU7); Figure S19: Receptor surface: Hydrophobicity of the surrounding amino acids around the binding pose of compound **3b**, **3d**, **3e** and **3f** at the upper binding pocket of SARS-CoV-2 main protease (PDB: 6LU7); Figure S20: Receptor surface: Hydrophobicity of the surrounding amino acids around the binding pose of compound **3a**, **3b**, **3d** and **3f** at the binding pocket of human mast cell tryptase (PDB: 2ZA5). Table S1: Prediction of in-silico lipophilicity, water solubility and pharmacokinetic parameters of novel spiro[5,8-methanoquinazoline-2,3’-indoline]-2’,4-dione derivatives **3a**–**g**, **3i**–**k**, **3m** by SwissADME webtool; Table S2: Prediction of *in silico* druglikeness properties of novel spiro[5,8-methanoquinazoline-2,3’-indoline]-2’,4-dione derivatives **3a**–**g**, **3i**–**k**, **3m** by SwissADME web tool. References [59,60,65,66,67] are cited in the Supplementary Materials.
