# Supplementary material for: Synthesis and Docking Studies of Novel Spiro[5,8-methanoquinazoline-2,3′-indoline]-2′,4-dione Derivatives
Source: Molecules. 2024 Oct 29;29(21):5112. doi: 10.3390/molecules29215112 (PMC11547464; doi:10.3390/molecules29215112)
Supplement: Supplementary file 1 [file molecules-29-05112-s001.zip › molecules-3274507-supplementary.pdf]

Supplementary Information

Synthesis and docking studies of novel spiro[5,8-methanoquinazoline-2,3'-indoline]-2',4-dione derivatives

Tünde Faragó[a], Rebeka Mészáros[a], Edit Wéber[b], Márta Palkó[a*]

[a.] *Institute of Pharmaceutical Chemistry, Interdisciplinary Excellence Center, University of Szeged, Eötvös utca 6, Szeged H-6720, Hungary.*
[b.] *Department of Medical Chemistry and HUN-REN-SZTE Biomimetic Systems Research Group, University of Szeged, H-6720, Szeged, Hungary.*
* *Corresponding author, email: palko.marta@szte.hu*

Contents

1. ^{1}H and ^{13}C NMR spectra of the compounds 3a–p 2
2. Copies of HRMS-ESI Spectra of 3a–3p 20
3. Docking studies 46
4. SwissADME pharmacokinetic properties prediction 68

1. ^{1}H and ^{13}C NMR spectra of the compounds 3a–p

(2*R,4a*R**,5*R**,8*S**,8a*S**)-4a,5,8,8a-tetrahydro-1*H*-spiro[5,8-methanoquinazoline-2,3'-indoline]-2',4(3*H*)-dione (3a)**

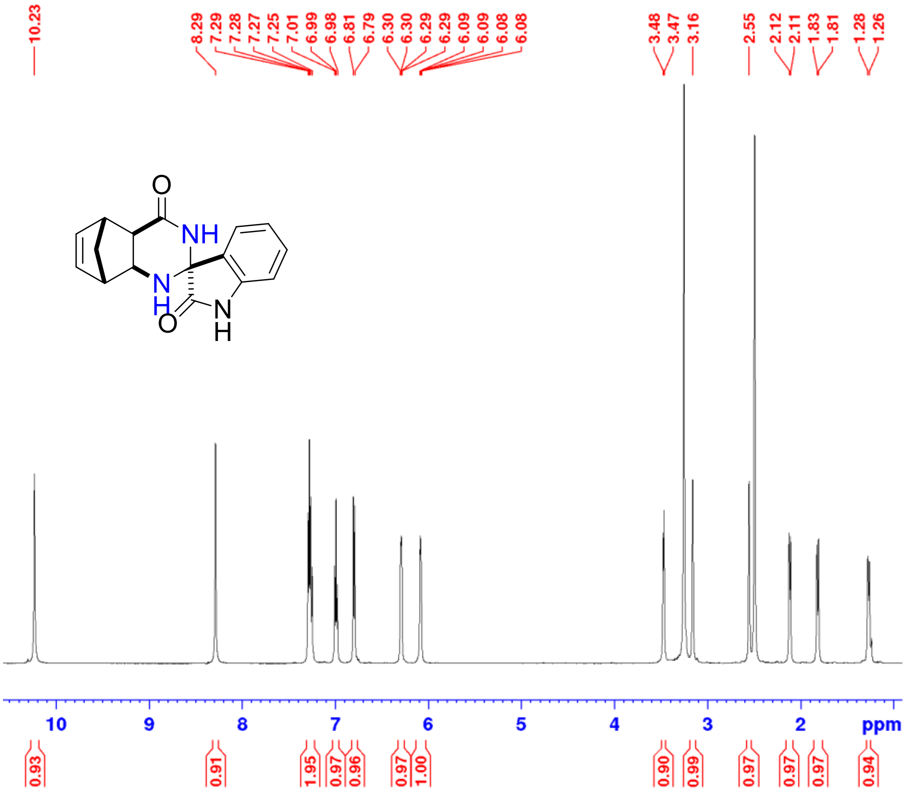

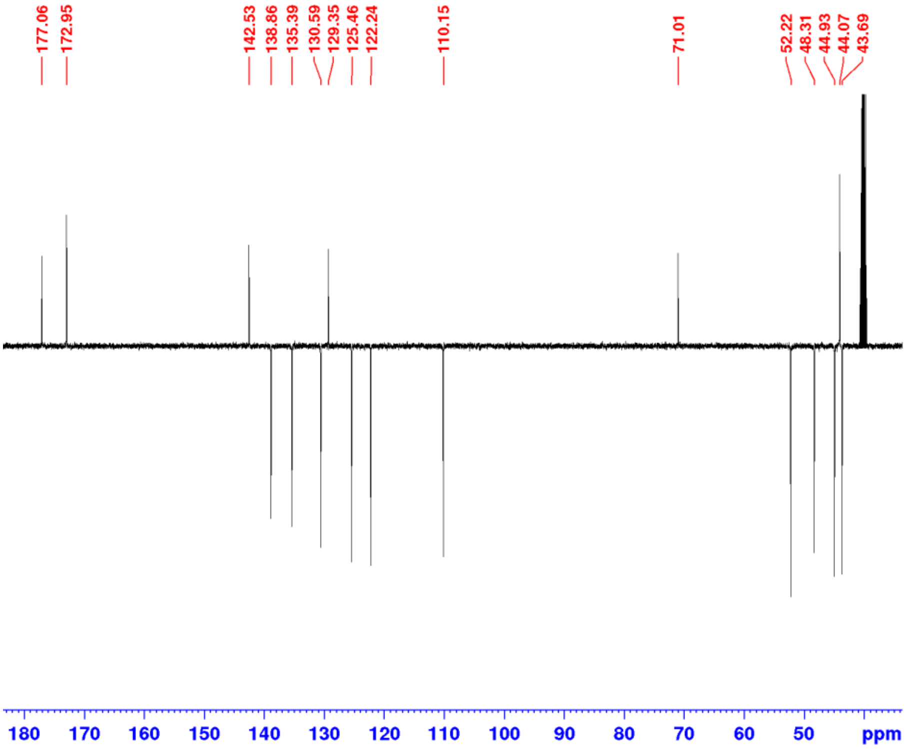

(2*R,4a*R**,5*R**,8*S**,8a*S**)-5'-methyl-4a,5,8,8a-tetrahydro-1*H*-spiro[5,8-methanoquinazoline-2,3'-indoline]-2',4(3*H*)-dione (3b)**

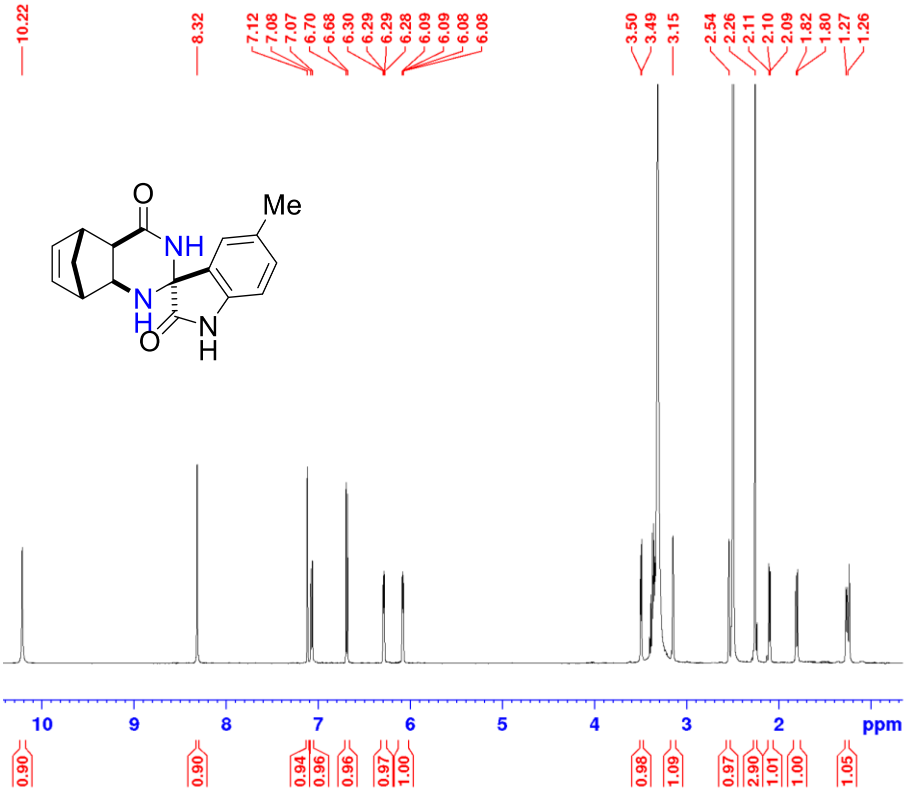

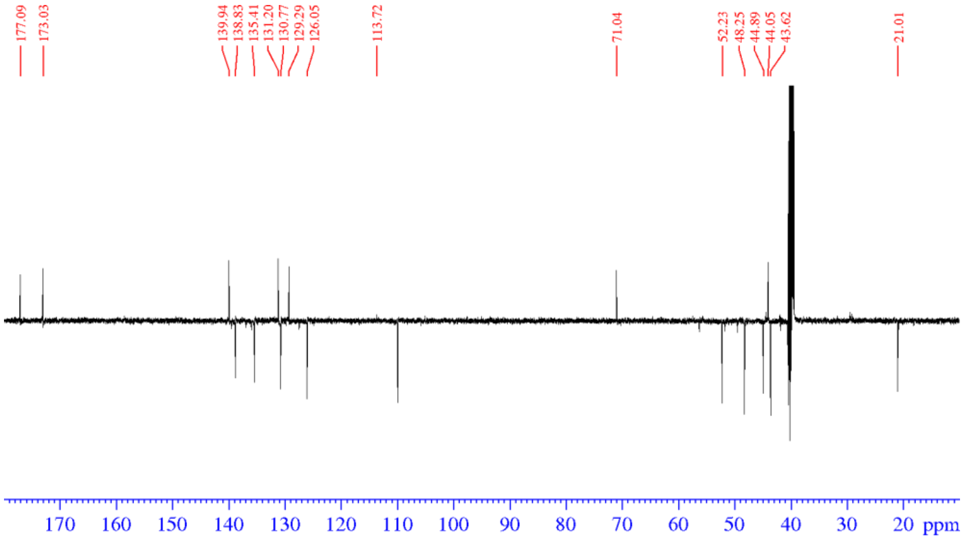

(2*R,4a*R**,5*R**,8*S**,8a*S**)-5'-iodo-4a,5,8,8a-tetrahydro-1*H*-spiro[5,8-methanoquinazoline-2,3'-indoline]-2',4(3*H*)-dione (3c)**

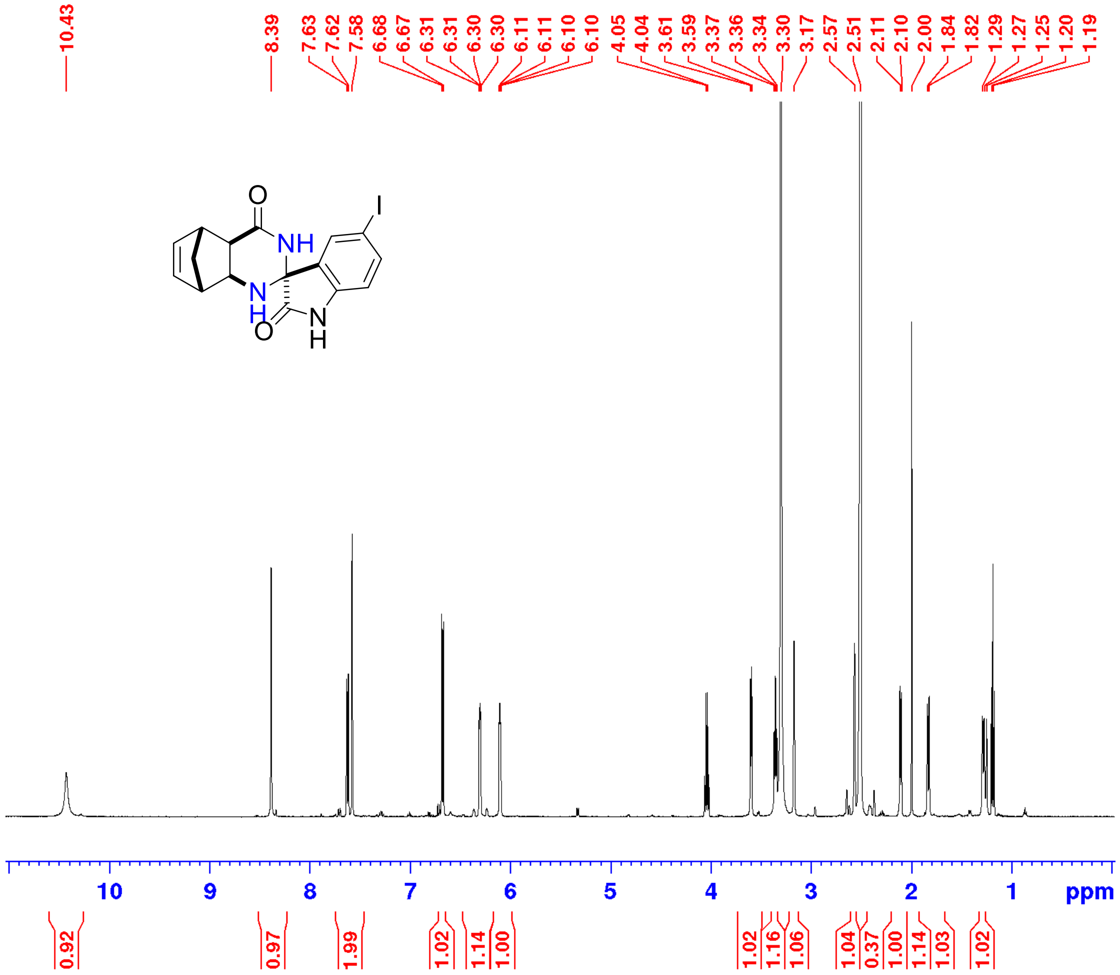

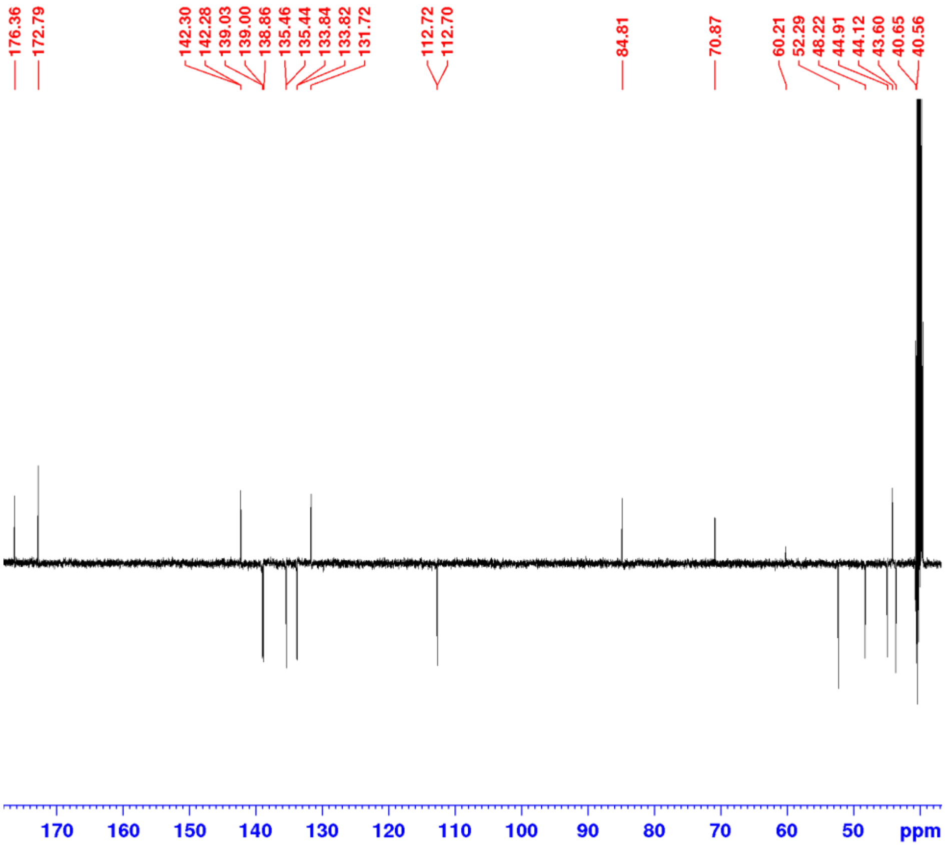

(2*R,4a*R**,5*R**,8*S**,8a*S**)-7'-chloro-4a,5,8,8a-tetrahydro-1*H*-spiro[5,8-methanoquinazoline-2,3'-indoline]-2',4(3*H*)-dione (3d)**

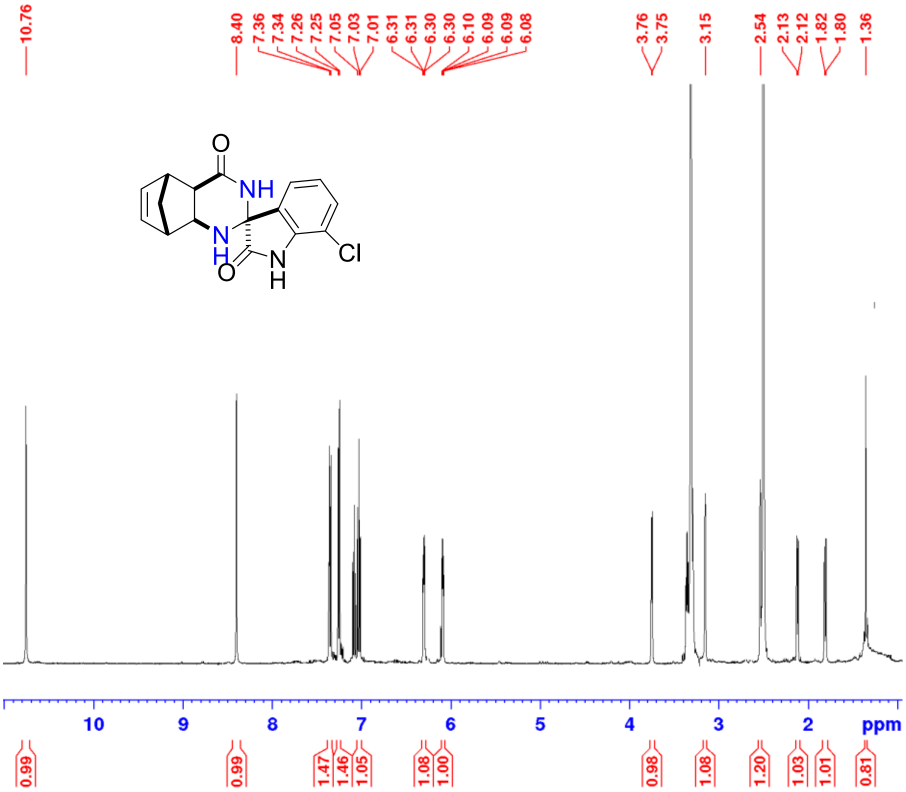

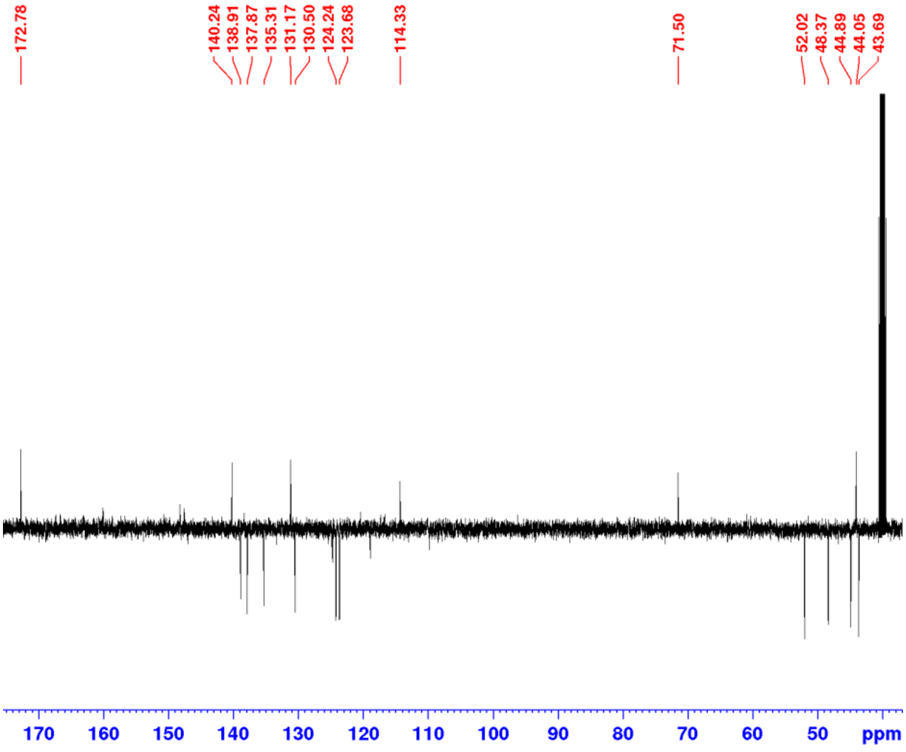

(2*S,4a*S**,5*R**,8*S**,8a*R**)-4a,5,8,8a-tetrahydro-1*H*-spiro[5,8-methanoquinazoline-2,3'-indoline]-2',4(3*H*)-dione (3e)**

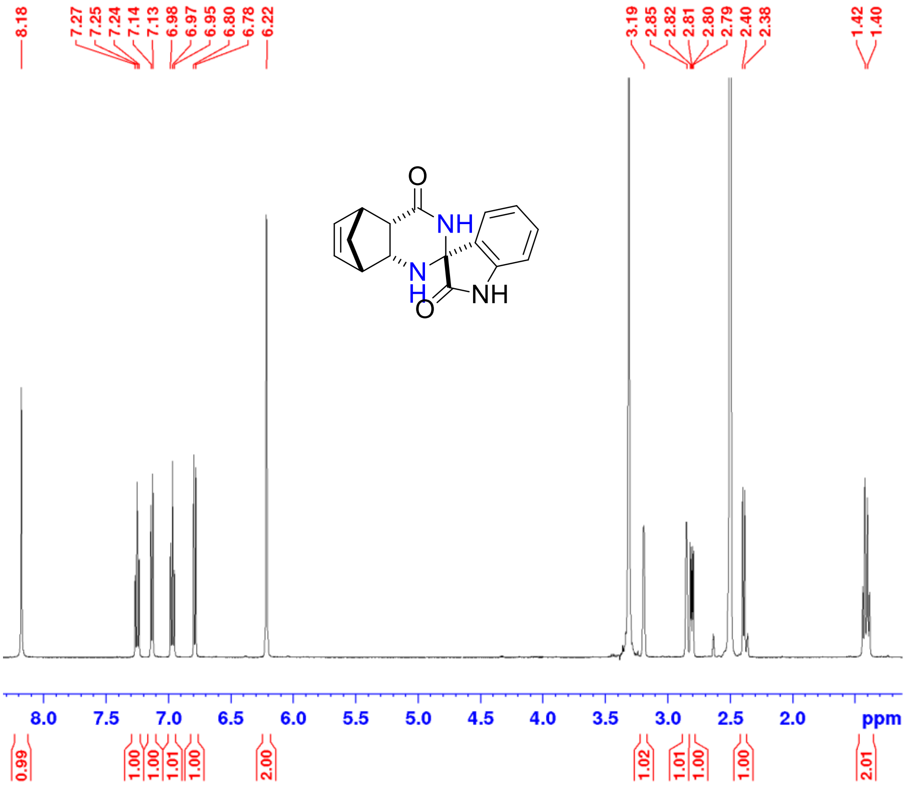

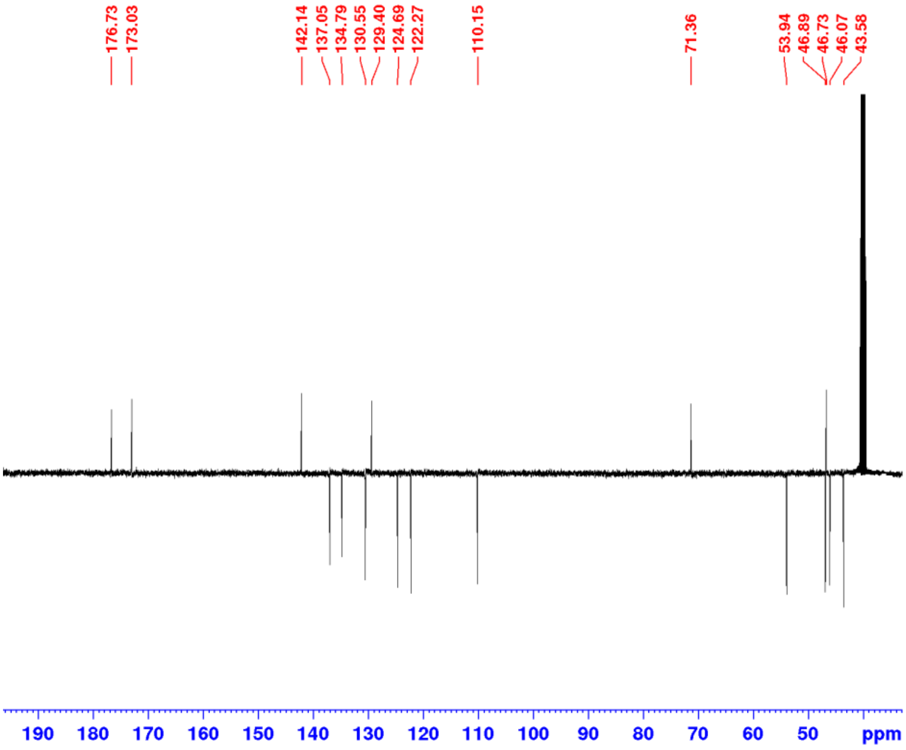

(2*S,4a*S**,5*R**,8*S**,8a*R**)-5'-methyl-4a,5,8,8a-tetrahydro-1*H*-spiro[5,8-methanoquinazoline-2,3'-indoline]-2',4(3*H*)-dione (3f)**

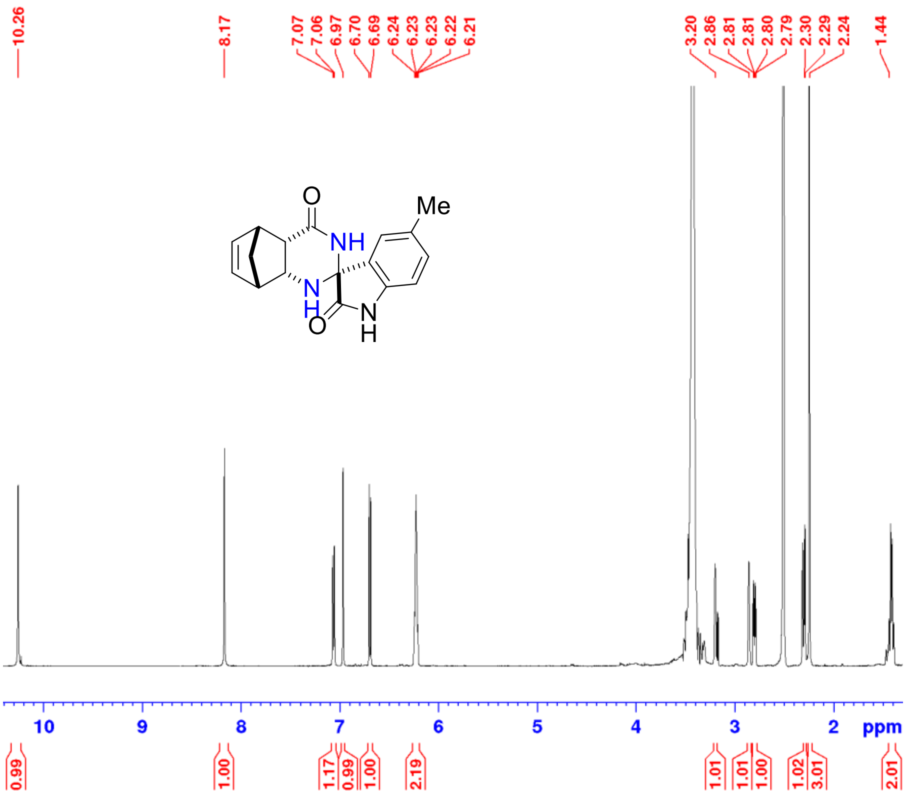

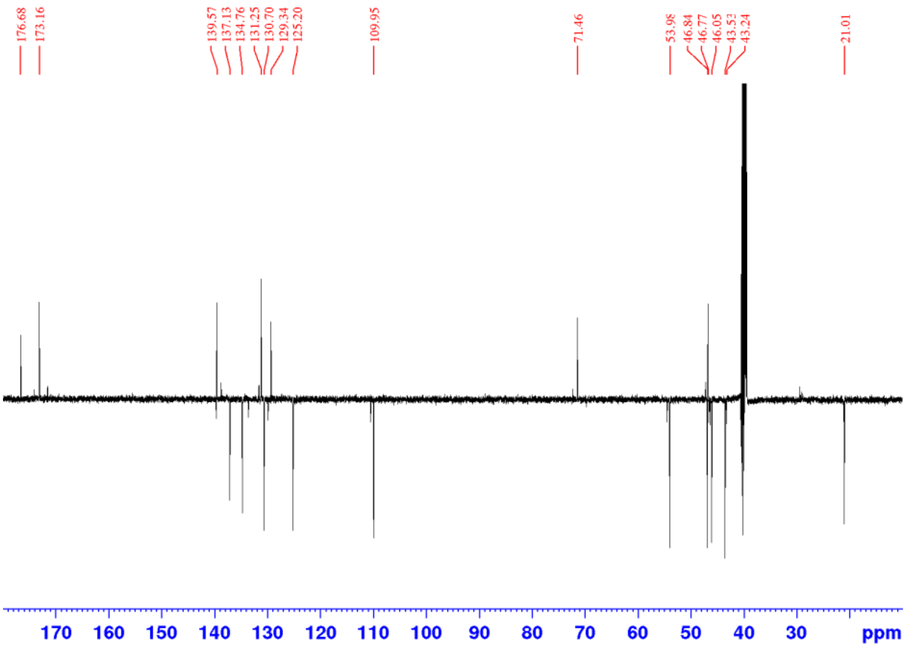

(2*S,4a*S**,5*R**,8*S**,8a*R**)-5'-iodo-4a,5,8,8a-tetrahydro-1*H*-spiro[5,8-methanoquinazoline-2,3'-indoline]-2',4(3*H*)-dione (3g)**

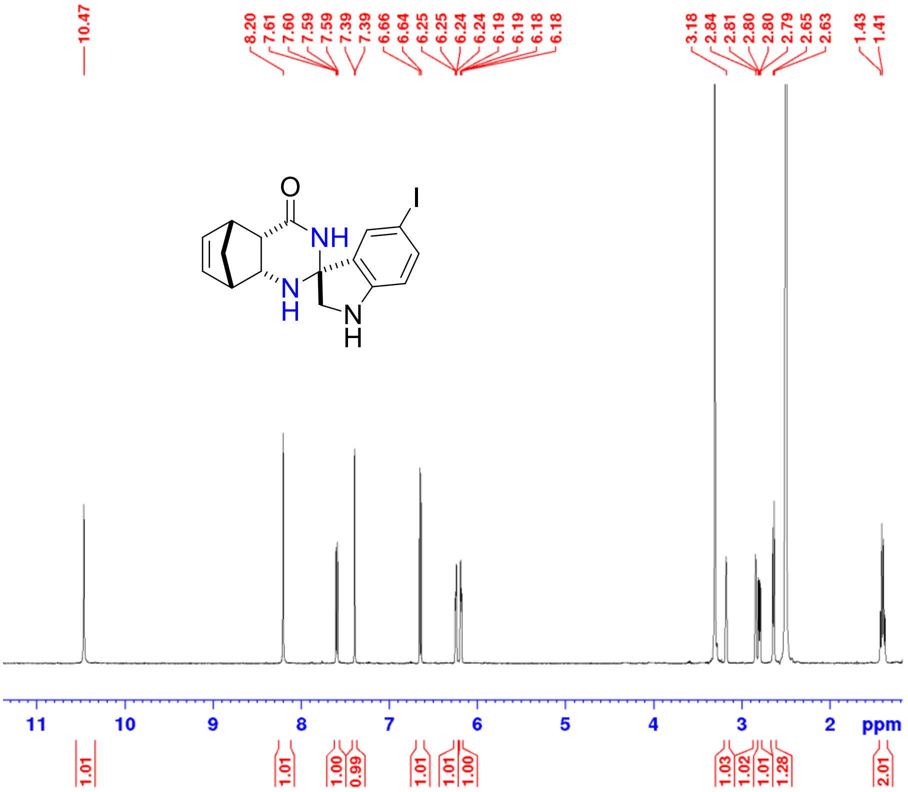

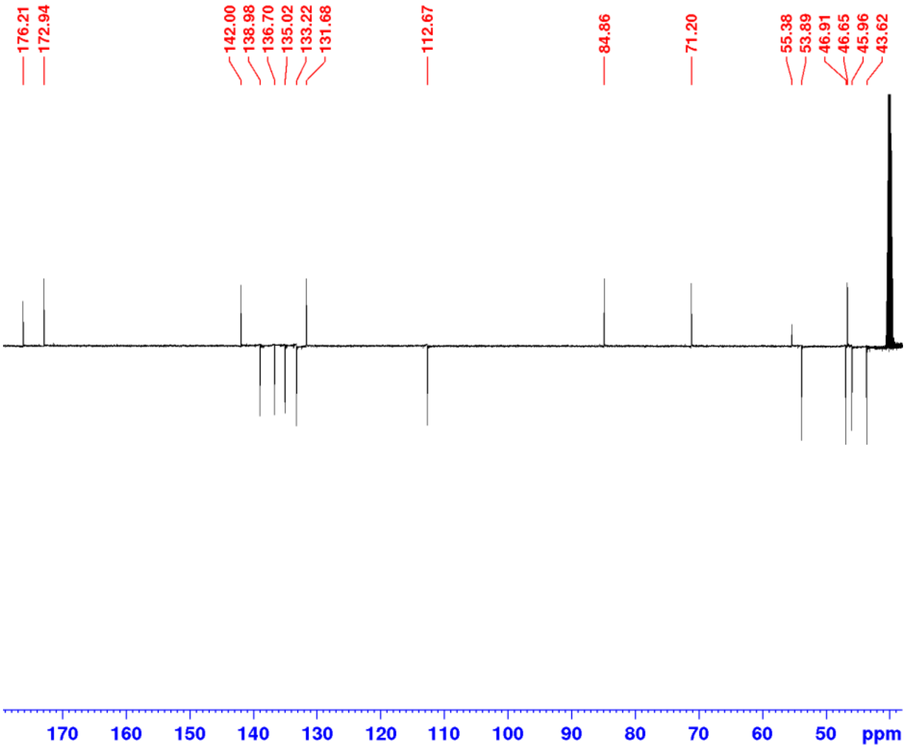

(4a*S,5*R**,8*S**,8a*R**)-7'-chloro-4a,5,8,8a-tetrahydro-1*H*-spiro[5,8-methanoquinazoline-2,3'-indoline]-2',4(3*H*)-dione (3h*)** (mixtures of diastereomers of 2*R** and 2*S**)

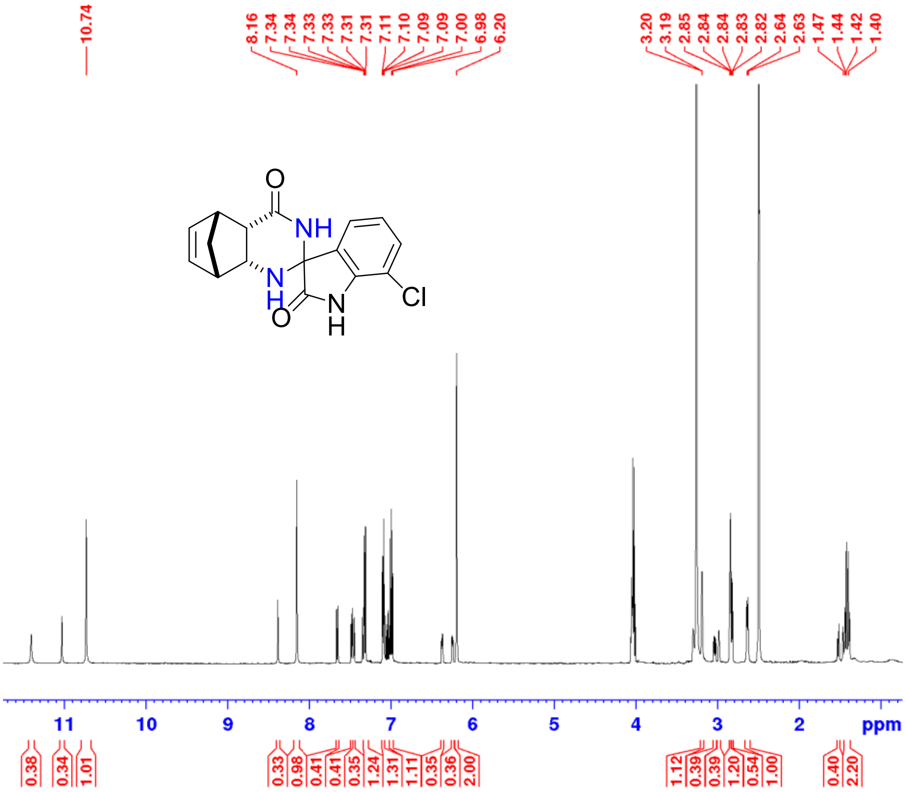

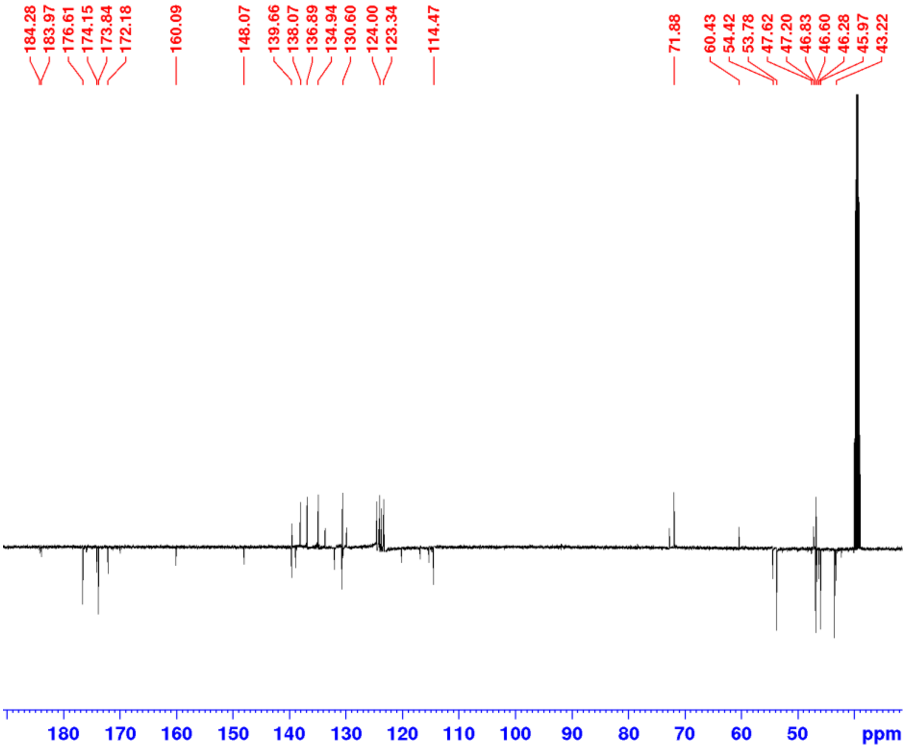

(2*R,4a*R**,5*R**,8*S**,8a*S**)-3-methyl-4a,5,8,8a-tetrahydro-1*H*-spiro[5,8-methanoquinazoline-2,3'-indoline]-2',4(3*H*)-dione (3i)**

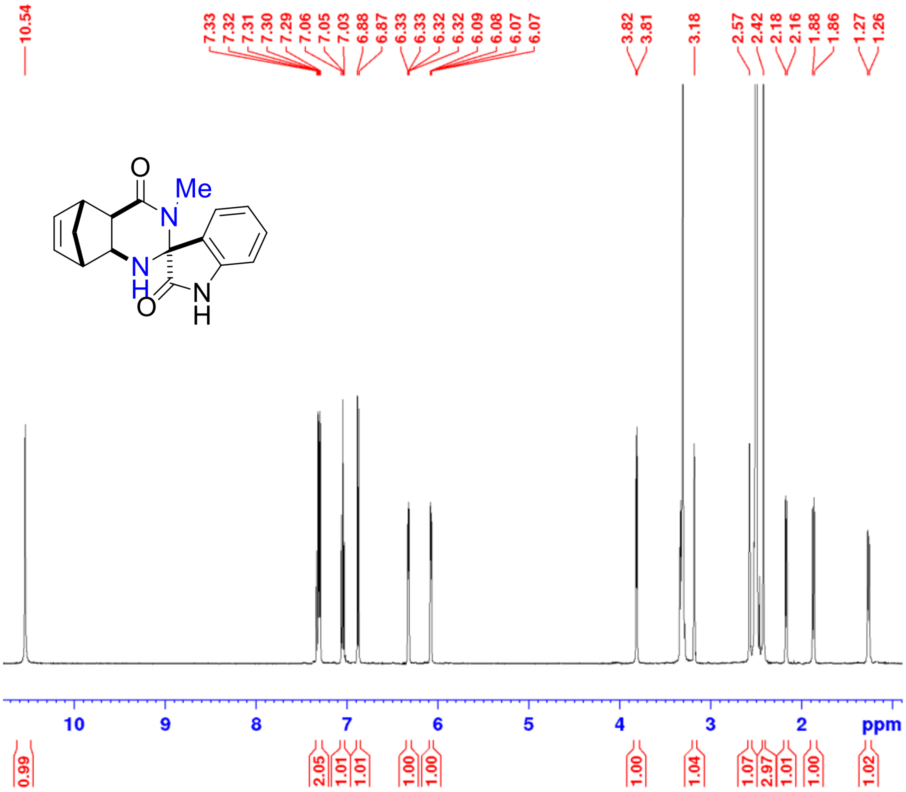

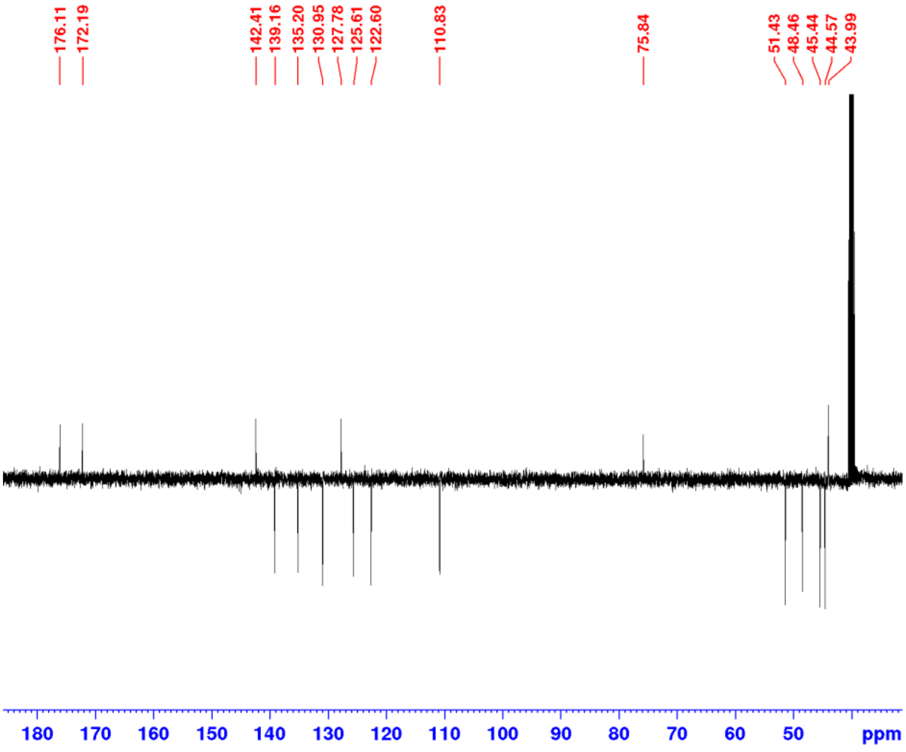

(2*R,4a*R**,5*R**,8*S**,8a*S**)-3,5'-dimethyl-4a,5,8,8a-tetrahydro-1*H*-spiro[5,8-methanoquinazoline-2,3'-indoline]-2',4(3*H*)-dione (3j)**

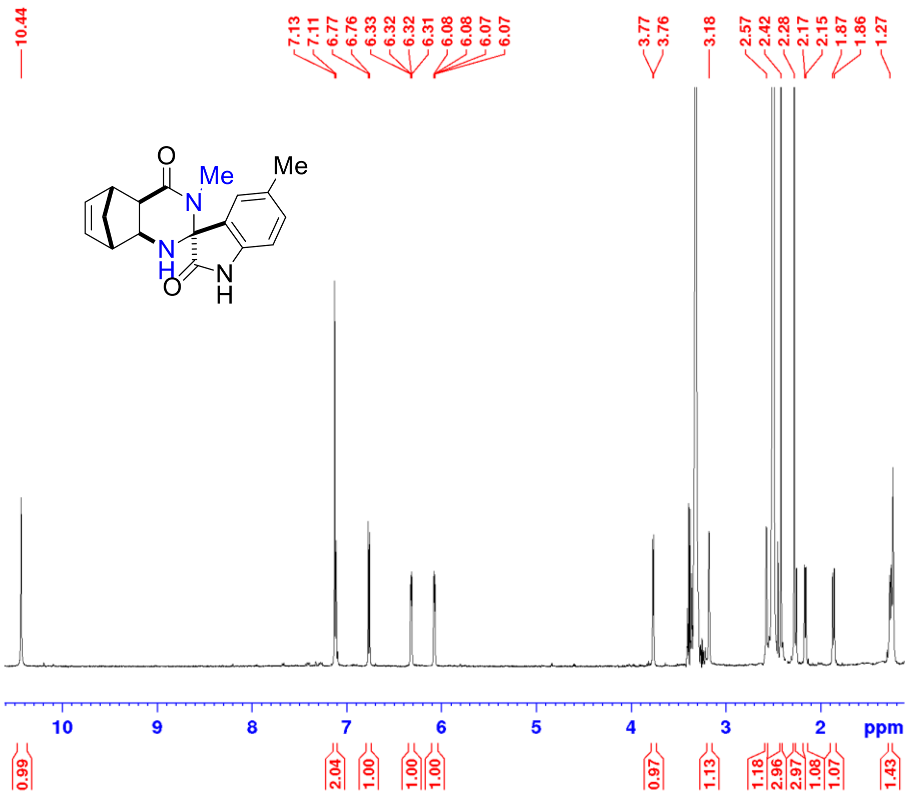

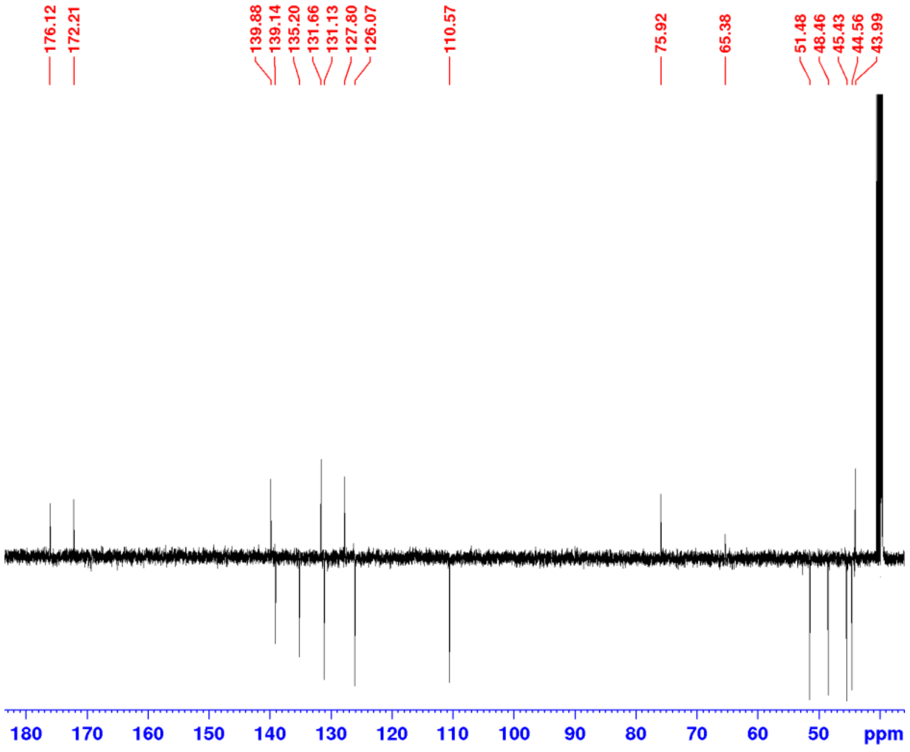

(2*R,4a*R**,5*R**,8*S**,8a*S**)-5'-iodo-3-methyl-4a,5,8,8a-tetrahydro-1*H*-spiro[5,8-methanoquinazoline-2,3'-indoline]-2',4(3*H*)-dione (3k)**

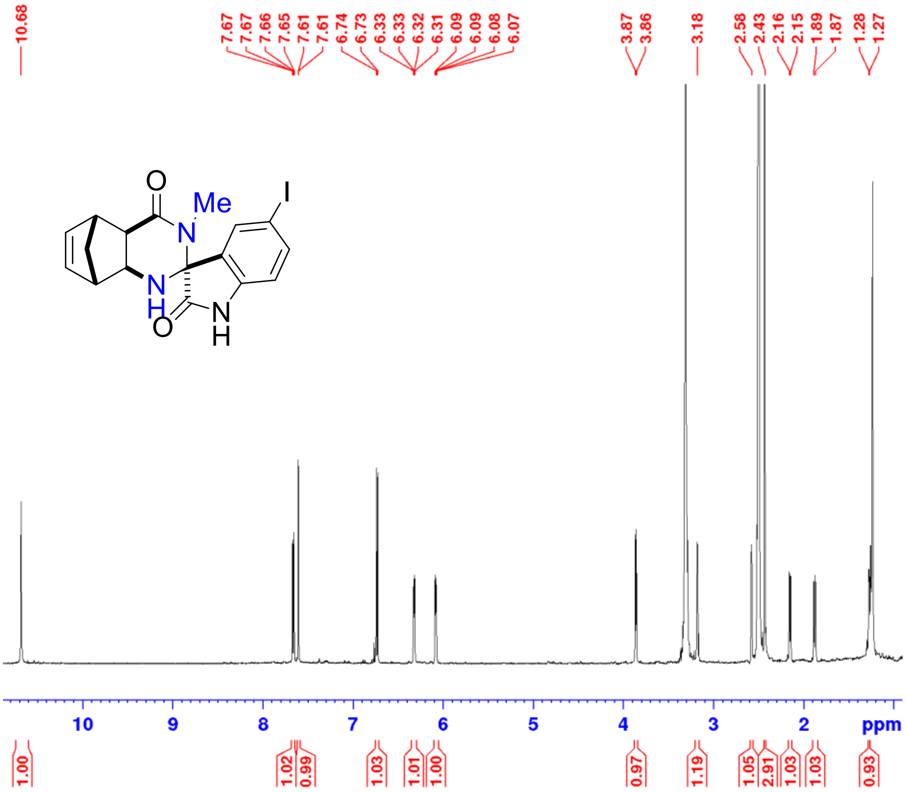

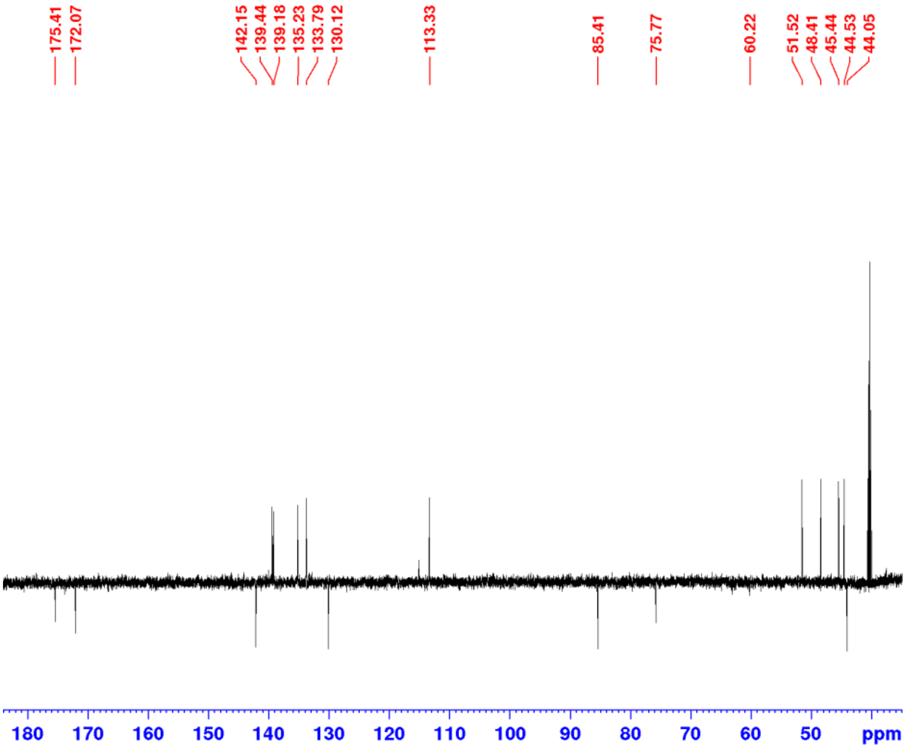

(4a*R,5*R**,8*S**,8a*S**)-7'-chloro-3-methyl-4a,5,8,8a-tetrahydro-1*H*-spiro[5,8-methanoquinazoline-2,3'-indoline]-2',4(3*H*)-dione (3l*)** (mixture of diastereomers, where the relative configuration of the major compound is 2*R**)

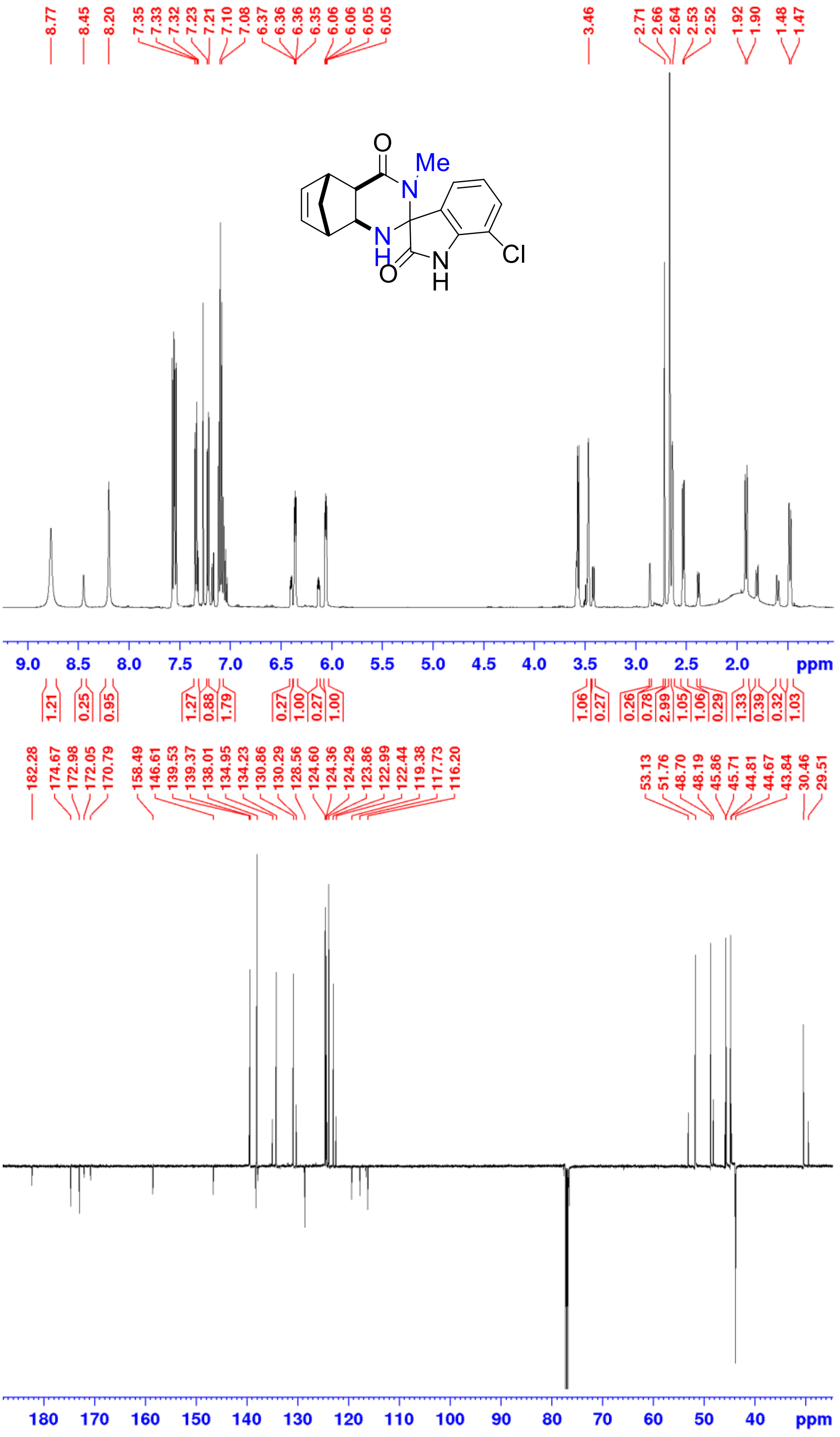

(2*S,4a*S**,5*R**,8*S**,8a*R**)-3-methyl-4a,5,8,8a-tetrahydro-1*H*-spiro[5,8-methanoquinazoline-2,3'-indoline]-2',4(3*H*)-dione (3m)**

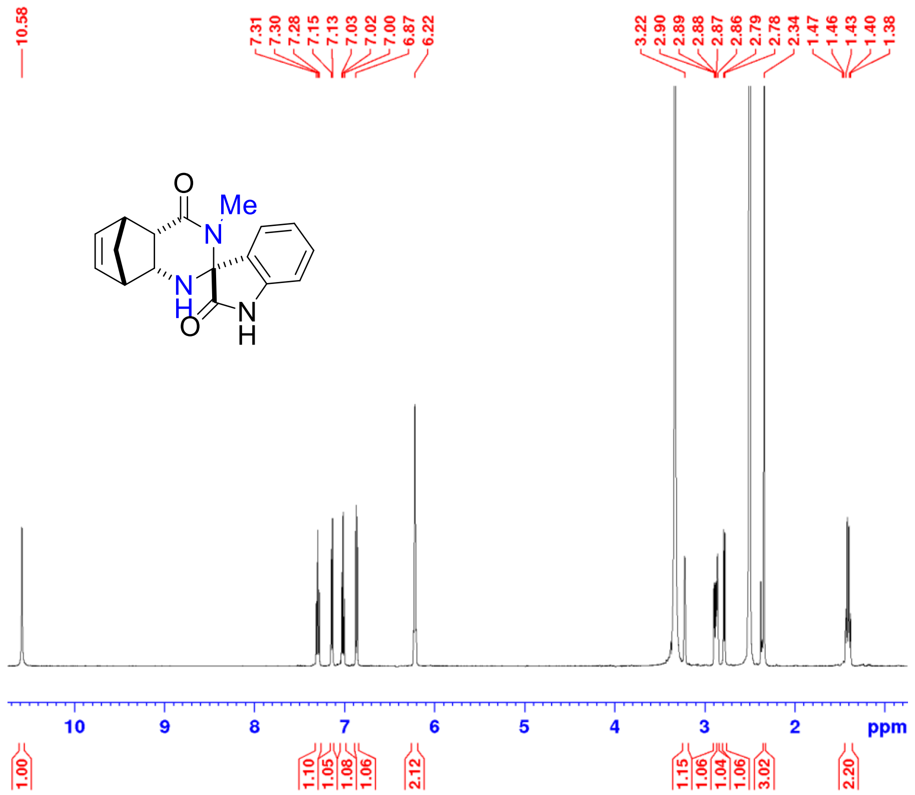

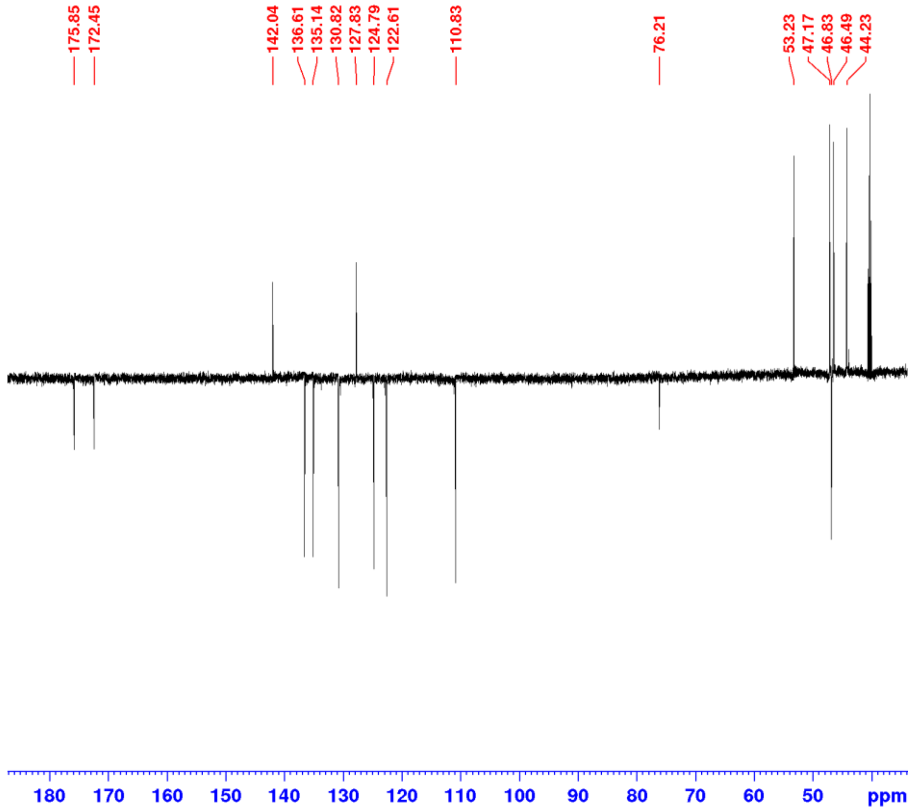

(4a*S,5*R**,8*S**,8a*R**)-3,5'-dimethyl-4a,5,8,8a-tetrahydro-1*H*-spiro[5,8-methanoquinazoline-2,3'-indoline]-2',4(3*H*)-dione (3n*)** (mixture of diastereomers of 2*R** and 2*S**)

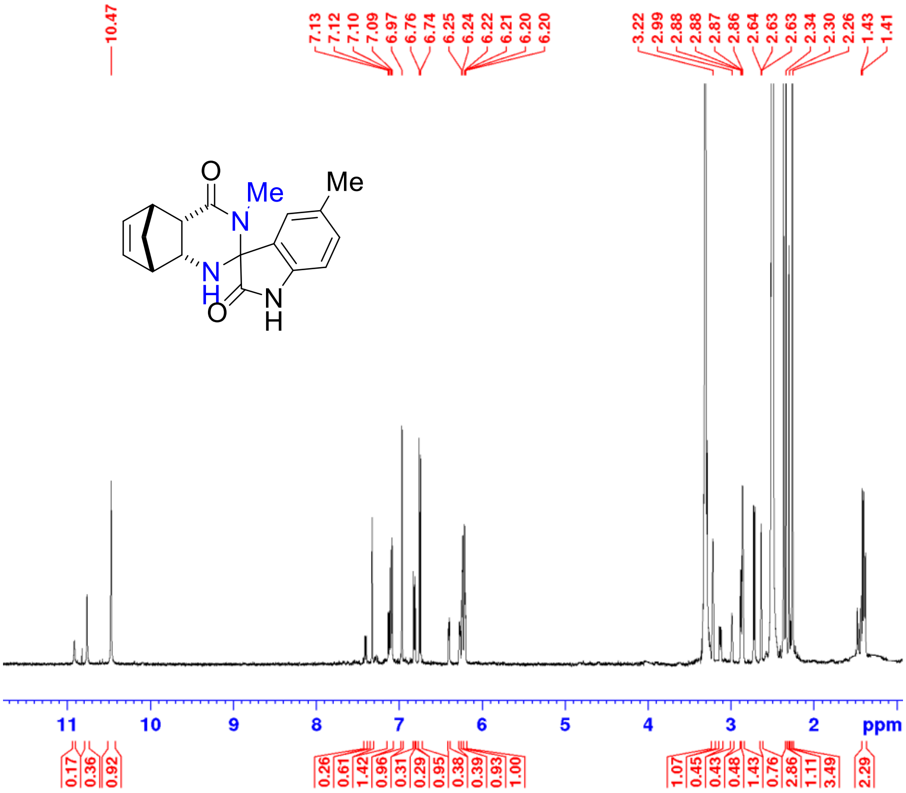

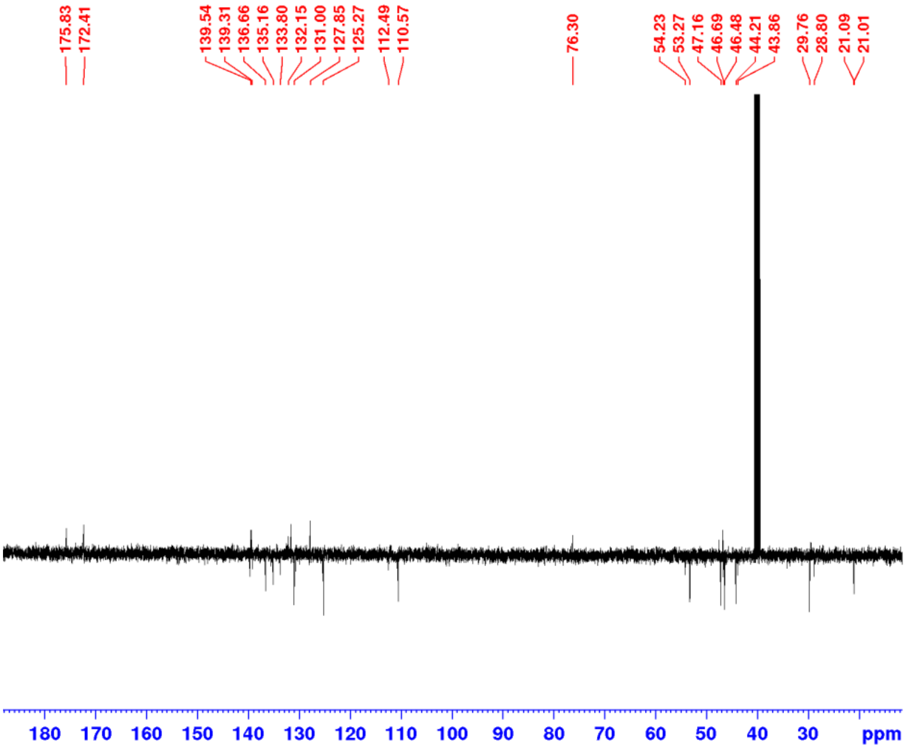

(4a*S,5*R**,8*S**,8a*R**)-5'-iodo-3-methyl-4a,5,8,8a-tetrahydro-1*H*-spiro[5,8-methanoquinazoline-2,3'-indoline]-2',4(3*H*)-dione (3o*)** (mixture of diastereomers of 2*R** and 2*S**)

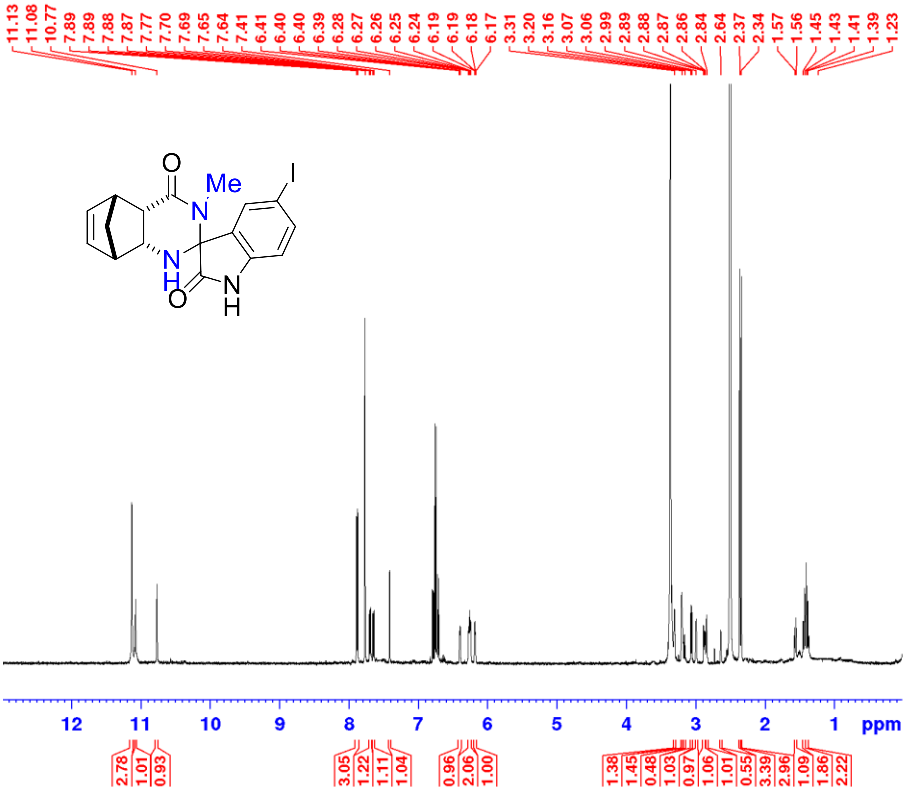

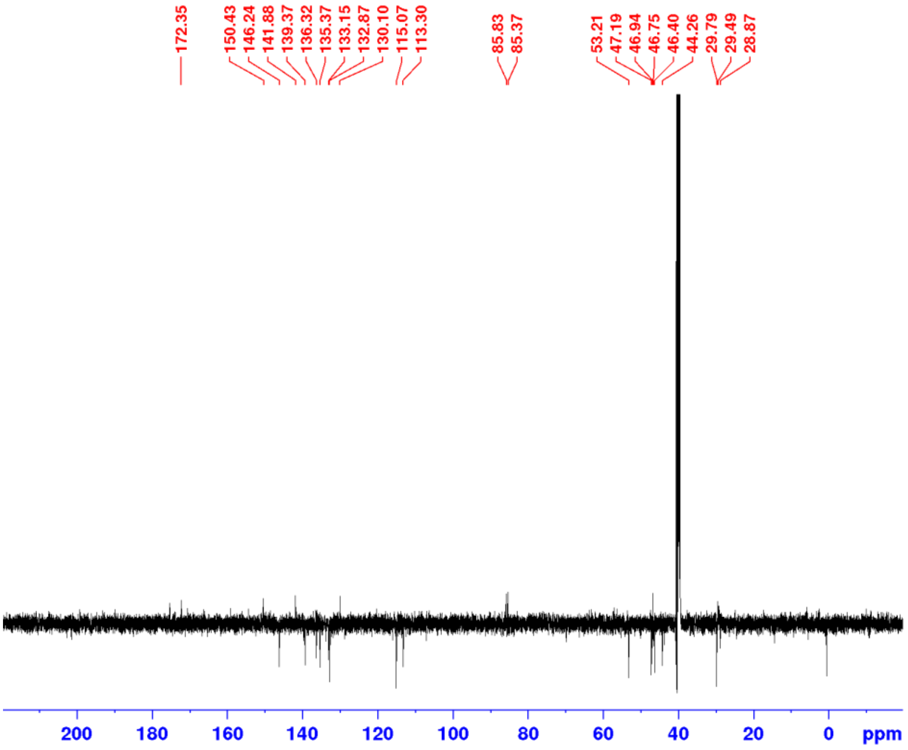

(4aS*,5R*,8S*,8aR*)-7'-chloro-3-methyl-4a,5,8,8a-tetrahydro-1*H*-spiro[5,8-methanoquinazoline-2,3'-indoline]-2',4(3*H*)-dione (3p*) (mixture of diastereomers of 2*R** and 2*S**)

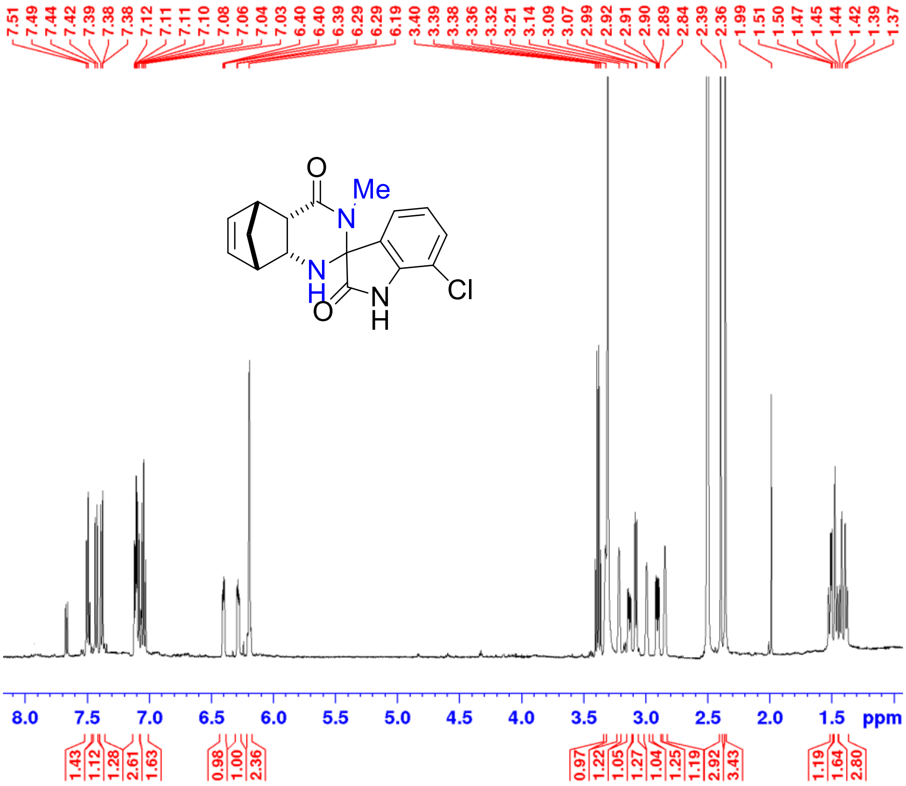

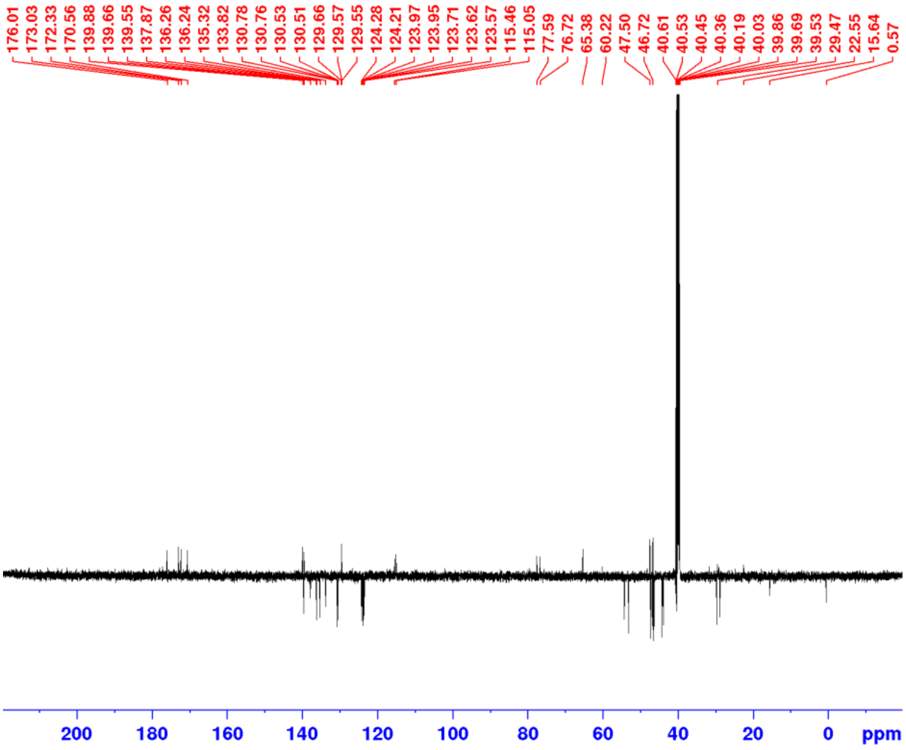

(1*R,2*R**,3*S**,4*S**)-3-((Z)-(5-methyl-2-oxoindolin-3-ylidene)amino)bicyclo[2.2.1]hept-5-ene-2-carboxamide (Bb)**

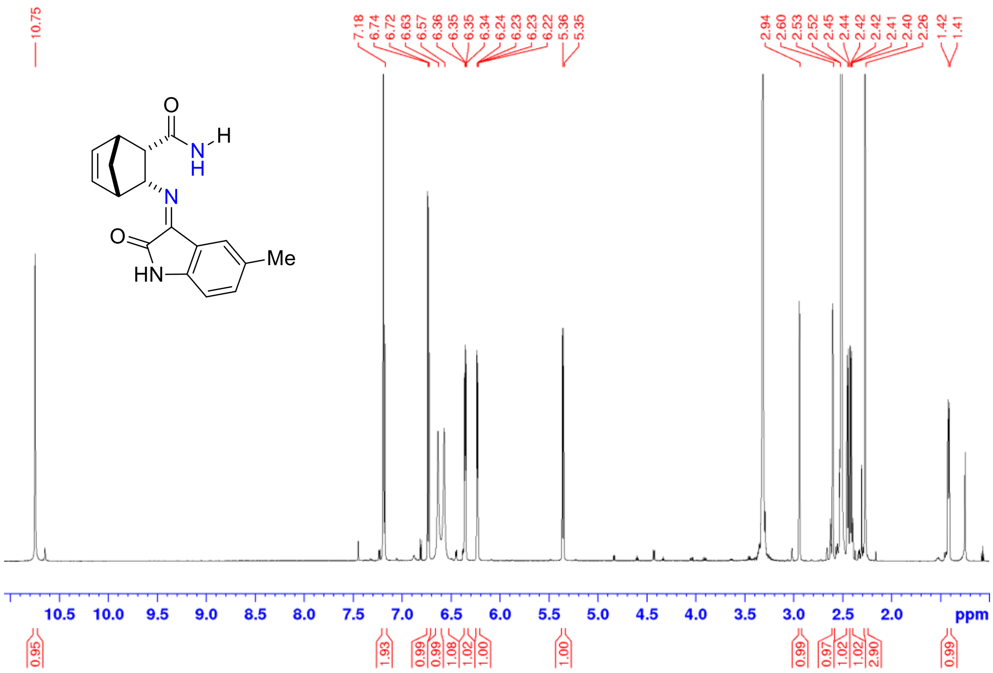

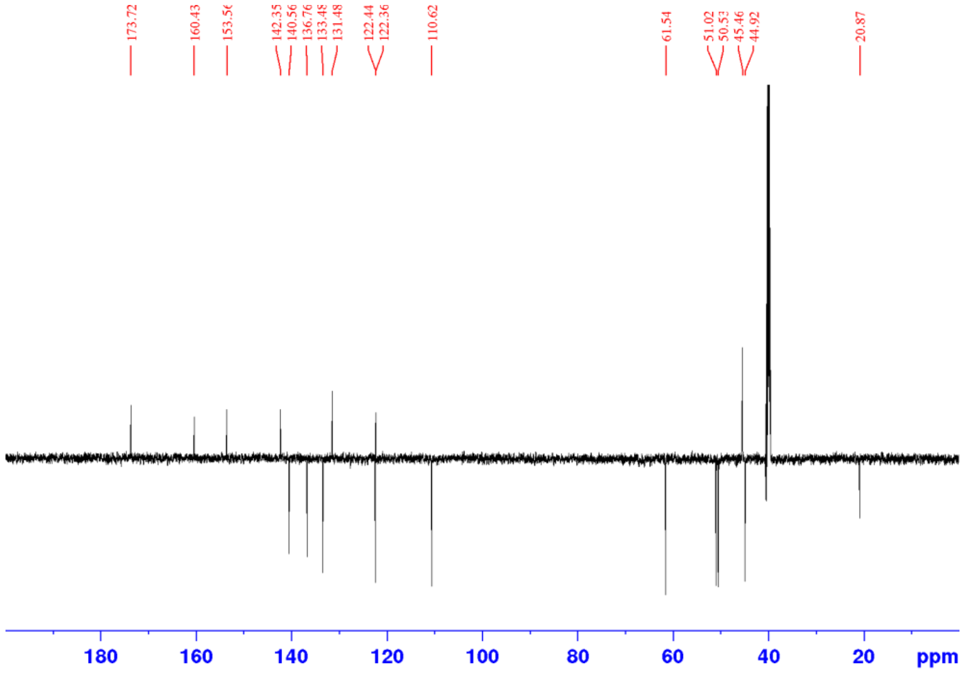

(1*R,2*R**,3*S**,4*S**)-3-((Z)-(7-chloro-2-oxoindolin-3-ylidene)amino)bicyclo[2.2.1]hept-5-ene-2-carboxamide (Bd)**

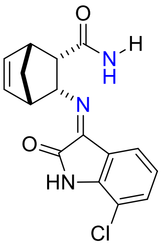

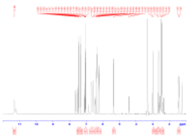

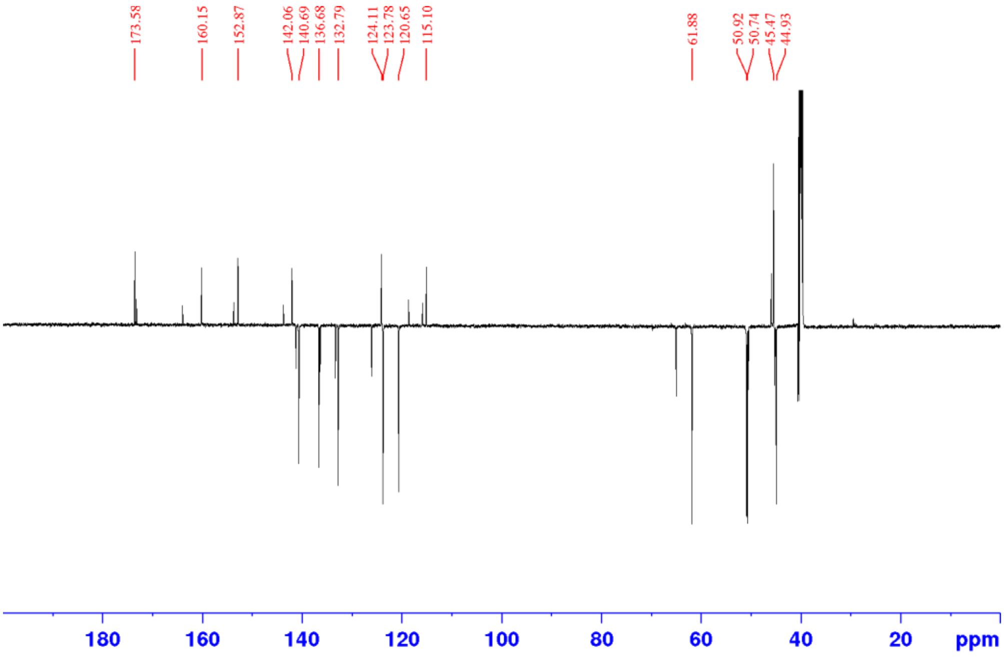

2. Copies of HRMS-ESI Spectra of 3a–3p

(2*R,4a*R**,5*R**,8*S**,8a*S**)-4a,5,8,8a-tetrahydro-*1H*-spiro[5,8-methanoquinazoline-2,3'-indoline]-2',4(3*H*)-dione (3a)**

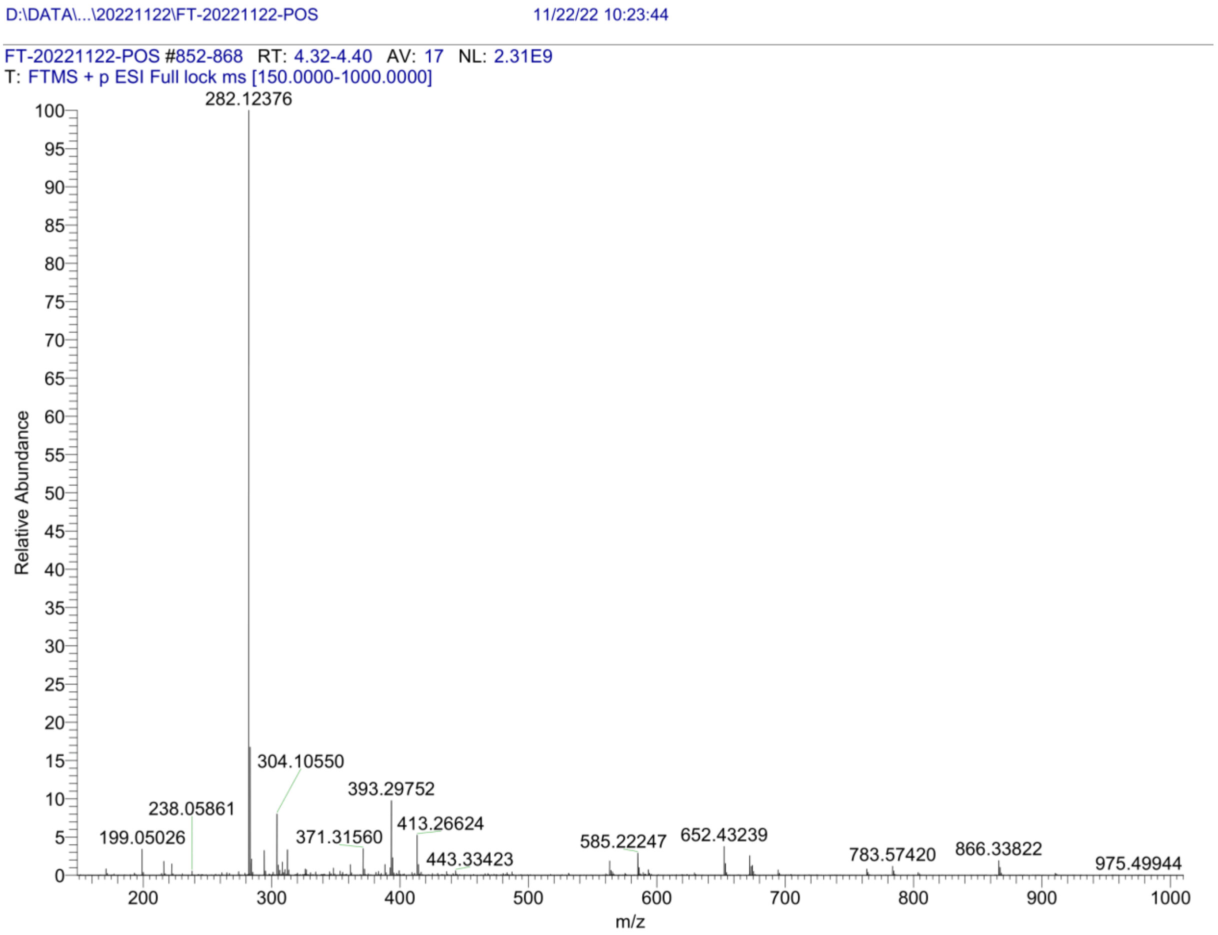

E:\MS-meresek (ujak)\4-es labor\FT144 10/19/2023 11:23:14 AM

RT: 0.00 - 1.00

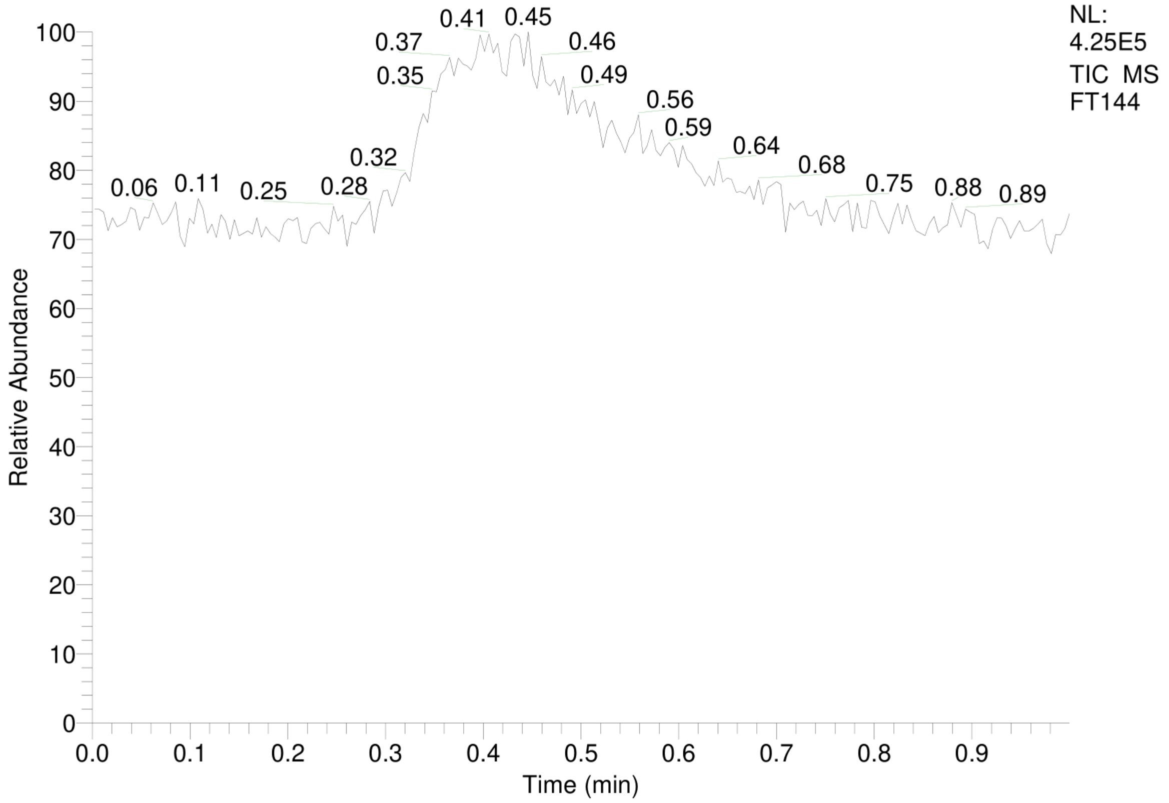

FT144 #70-112 RT: 0.32-0.51 AV: 43 NL: 2.15E4

T: ITMS + c ESI Full ms [50.00-2000.00]

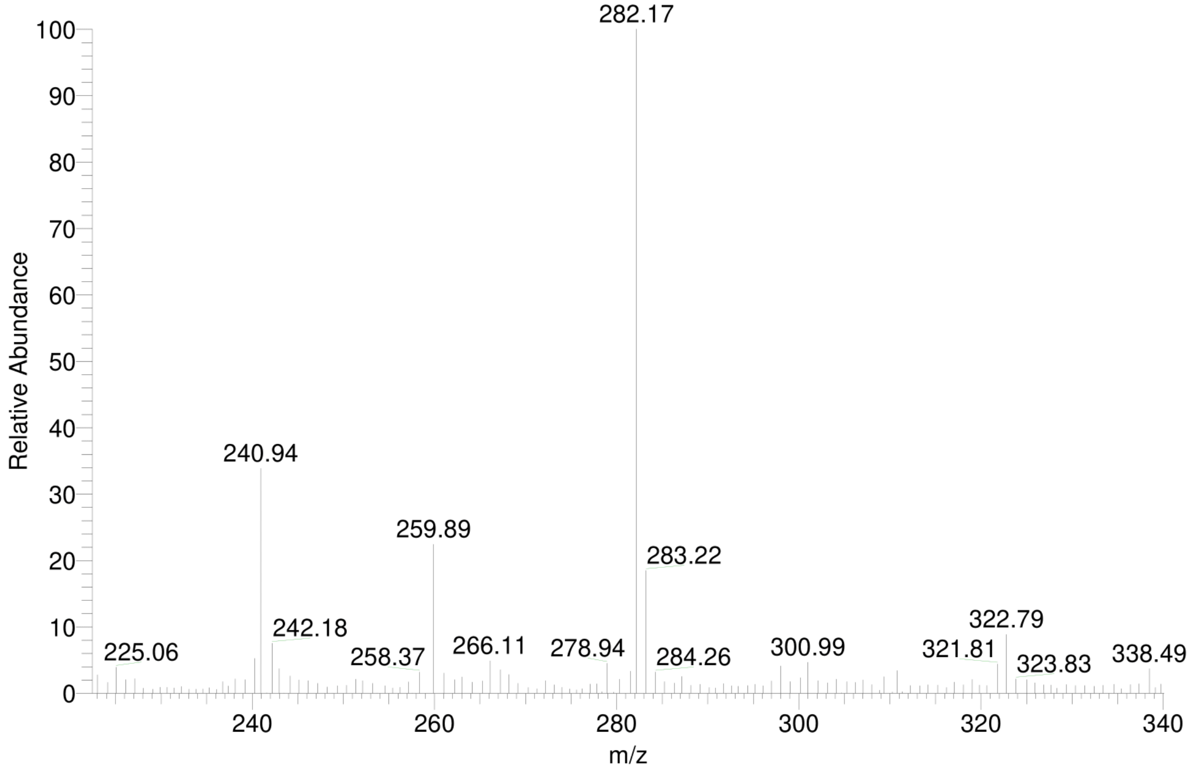

(2*R,4a*R**,5*R**,8*S**,8a*S**)-5'-methyl-4a,5,8,8a-tetrahydro-1*H*-spiro[5,8-methanoquinazoline-2,3'-indoline]-2',4(3*H*)-dione (3b)**

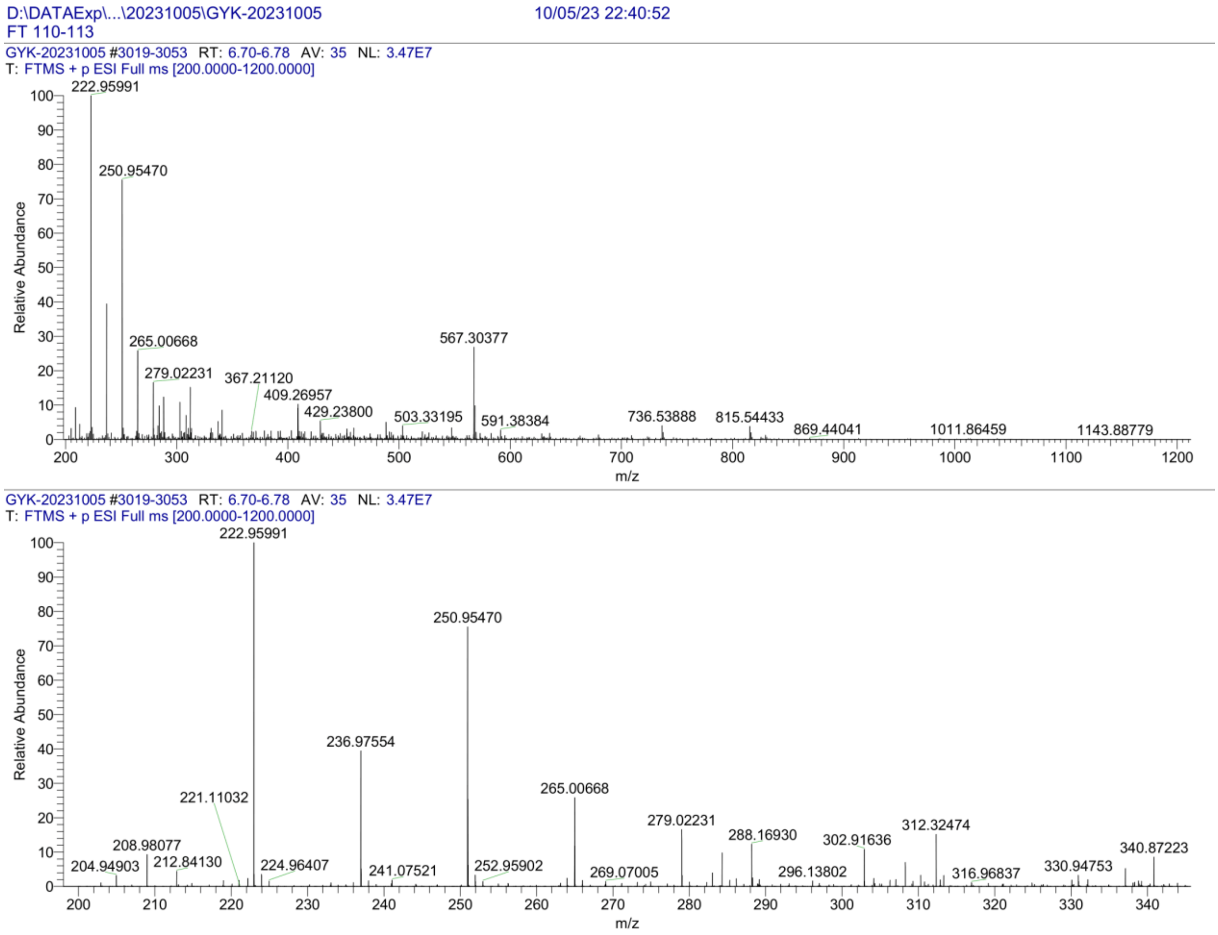

RT: 0.00 - 1.00

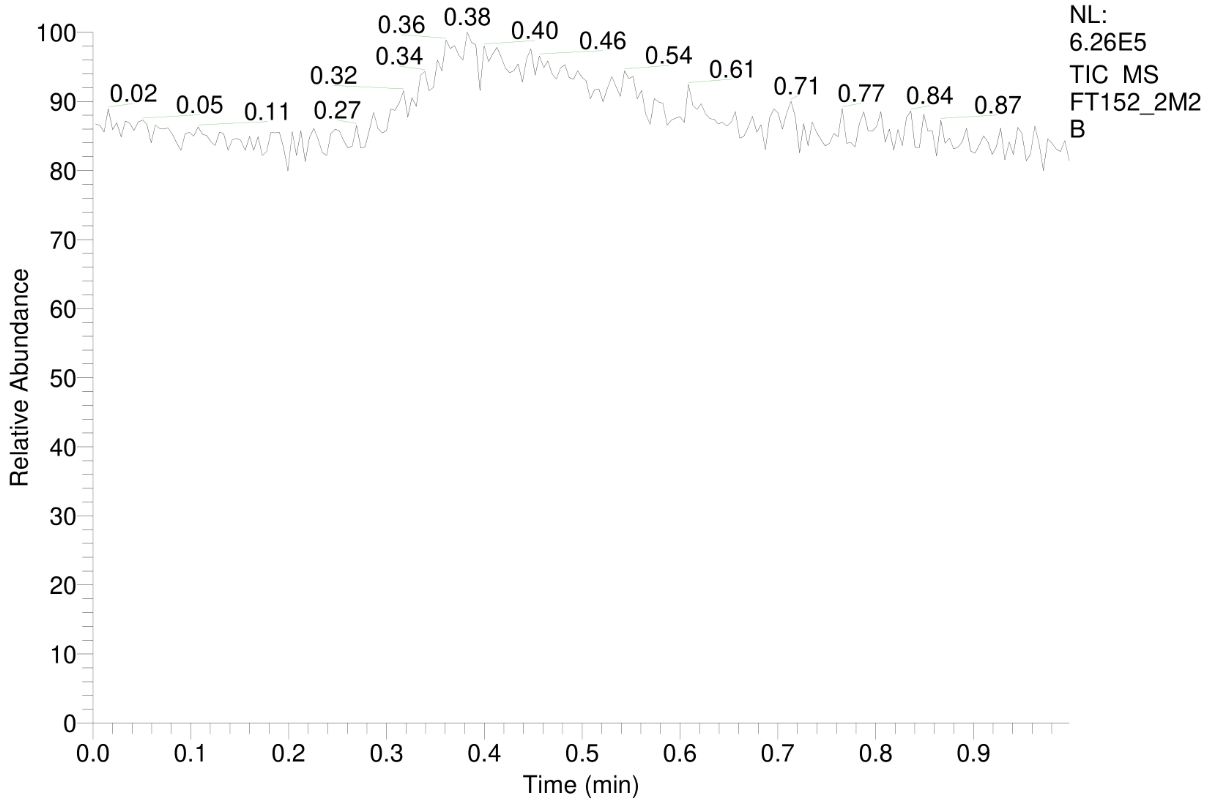

FT152_2M2B #73-117 RT: 0.32-0.51 AV: 45 NL: 1.65E4
T: ITMS + c ESI Full ms [50.00-2000.00]

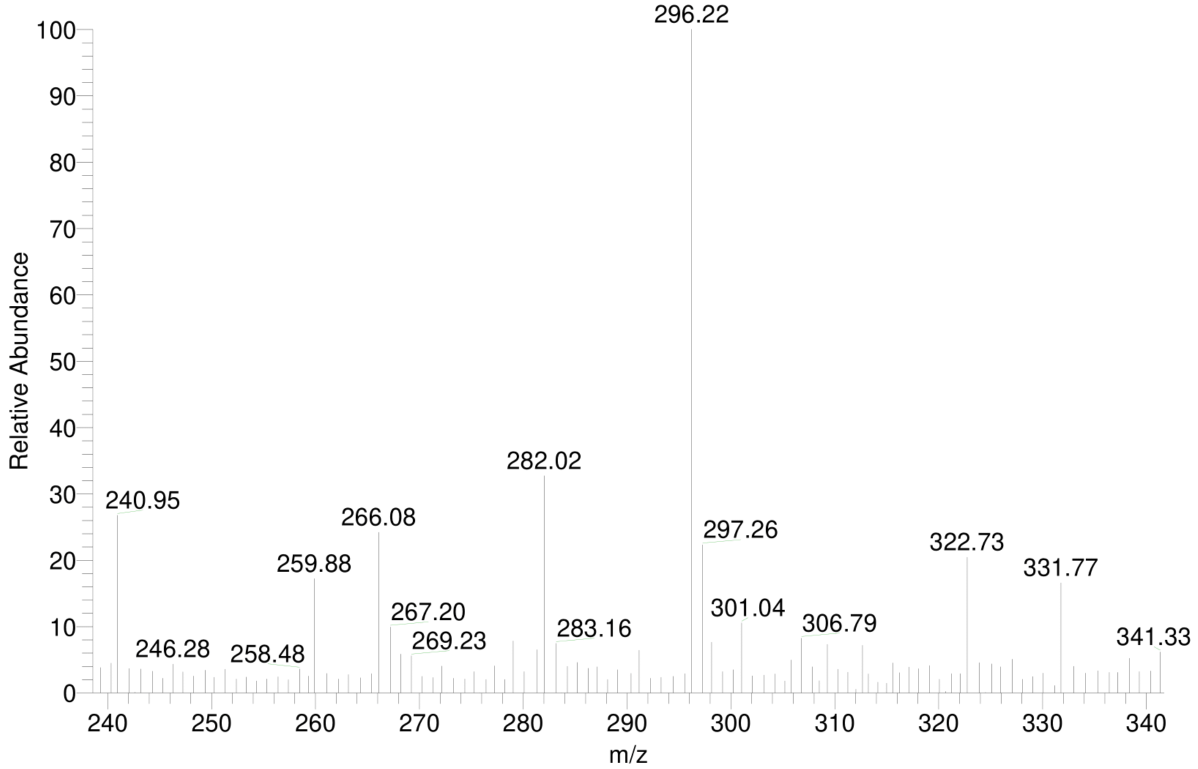

(2*R,4a*R**,5*R**,8*S**,8a*S**)-5'-iodo-4a,5,8,8a-tetrahydro-1*H*-spiro[5,8-methanoquinazoline-2,3'-indoline]-2',4(3*H*)-dione (3c)**

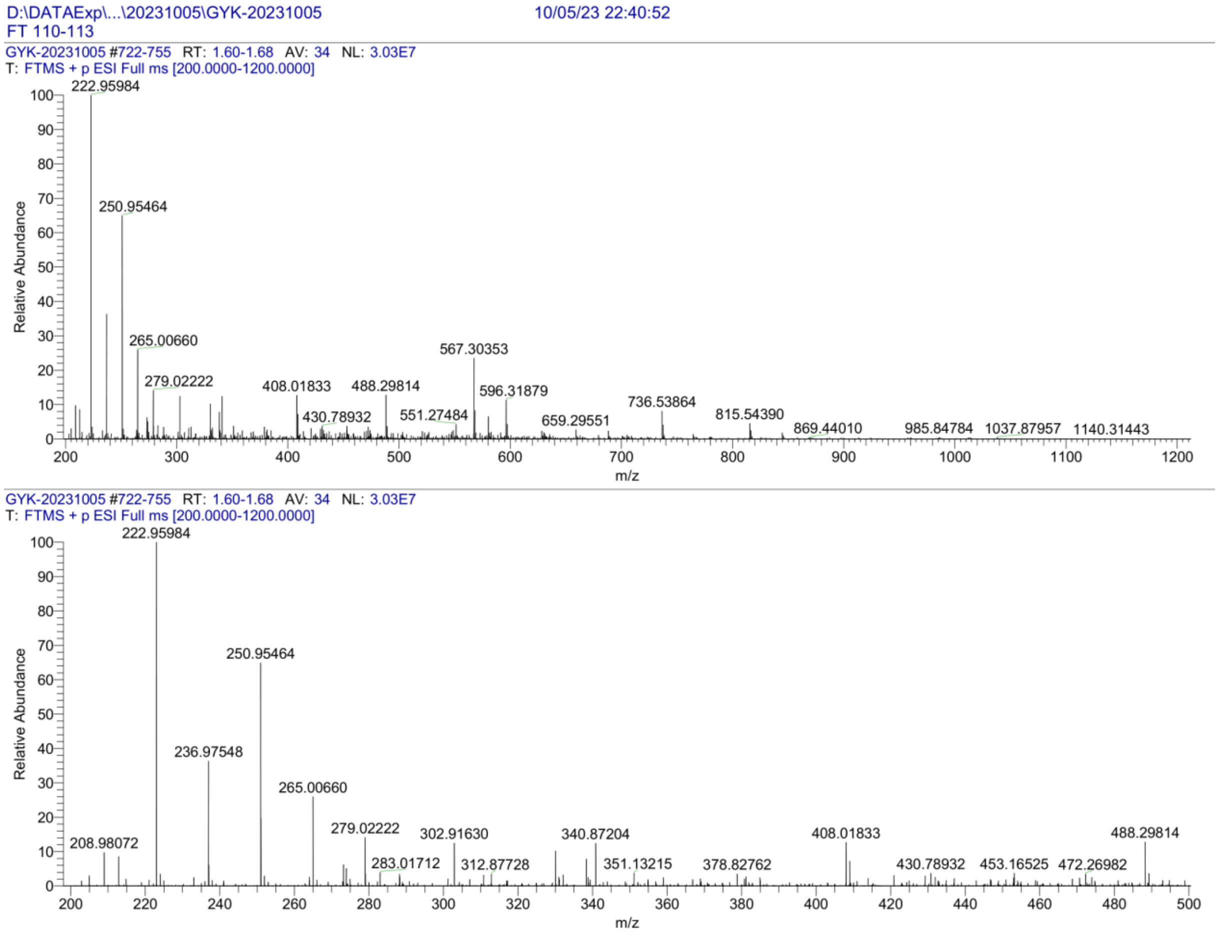

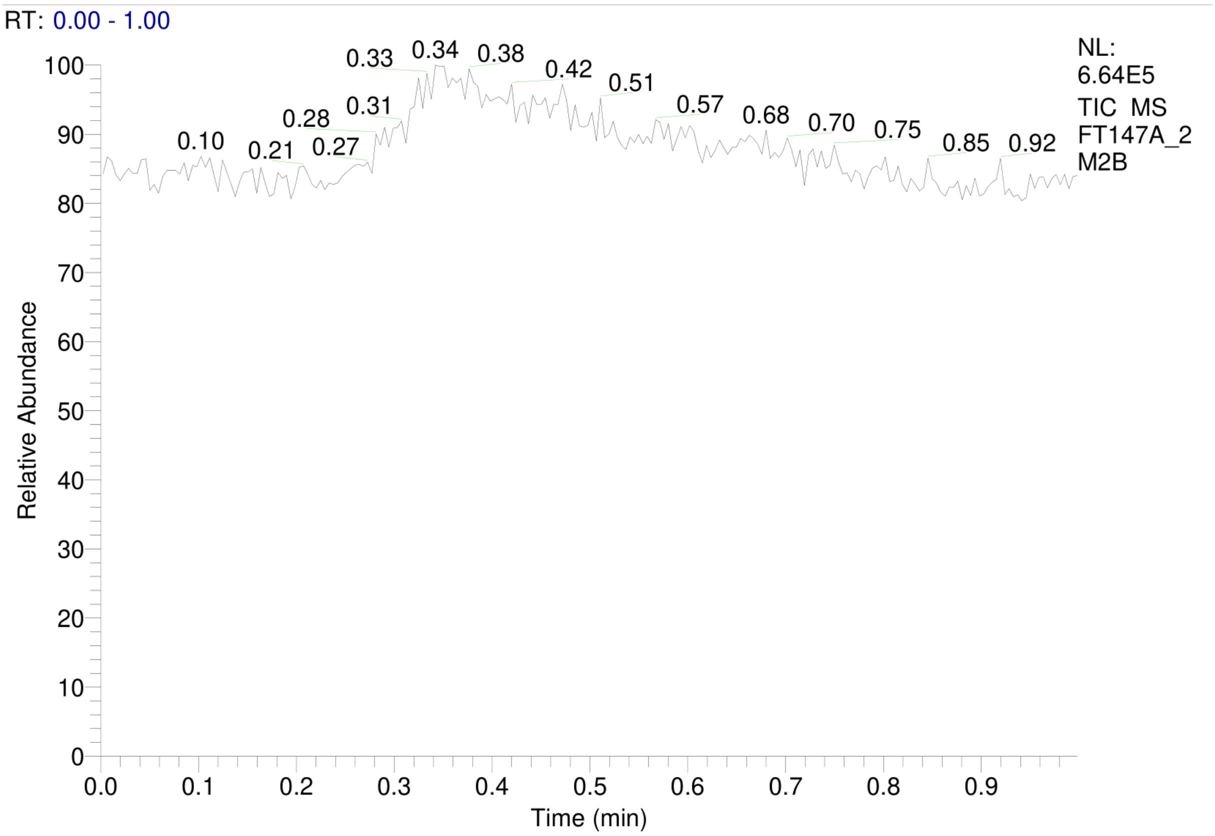
RT: 0.00 - 1.00
NL: 6.64E5
TIC MS FT147A_2M2B
0.33
0.34
0.38
0.42
0.51
0.57
0.68
0.70
0.75
0.85
0.92
0.10
0.21
0.28
0.27
0.31
Relative Abundance
Time (min)

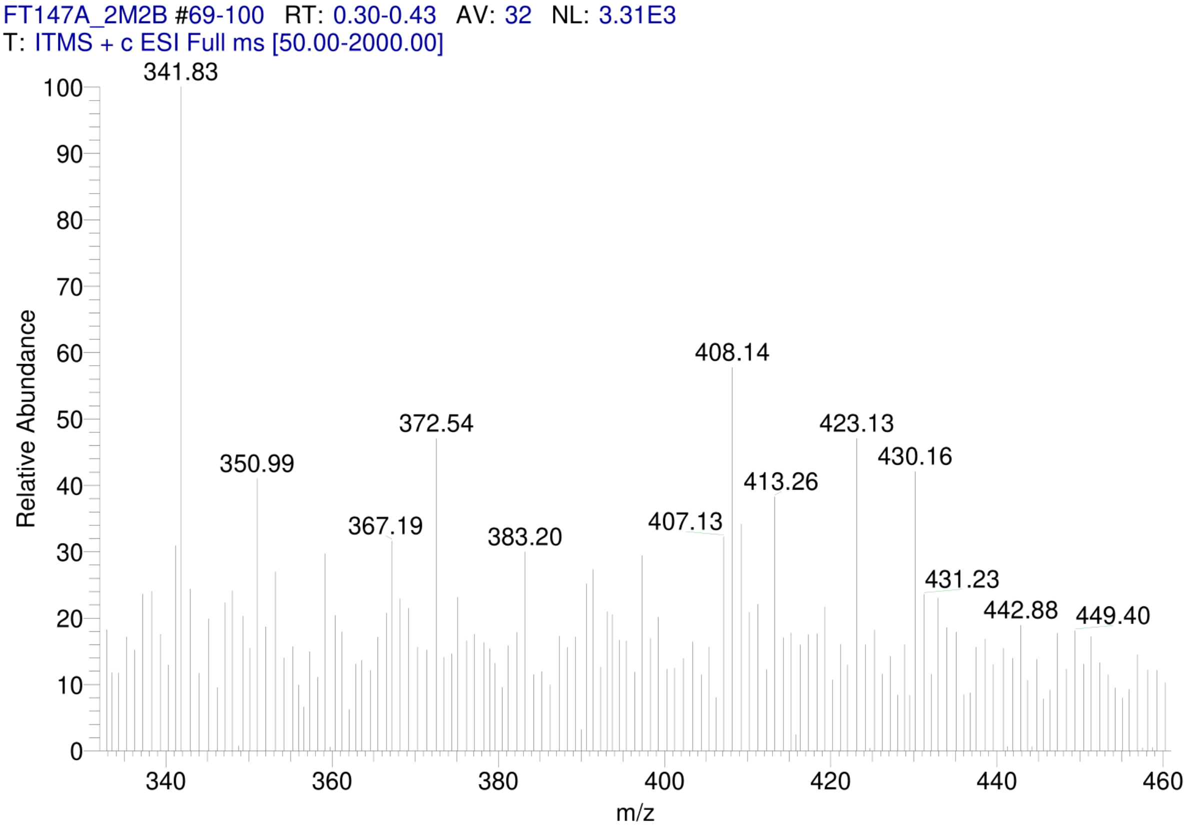
FT147A_2M2B #69-100 RT: 0.30-0.43 AV: 32 NL: 3.31E3
T: ITMS + c ESI Full ms [50.00-2000.00]
341.83
350.99
367.19
372.54
383.20
407.13
408.14
413.26
423.13
430.16
431.23
442.88
449.40
Relative Abundance
m/z

(2*R,4a*R**,5*R**,8*S**,8a*S**)-7'-chloro-4a,5,8,8a-tetrahydro-1*H*-spiro[5,8-methanoquinazoline-2,3'-indoline]-2',4(3*H*)-dione (3d)**

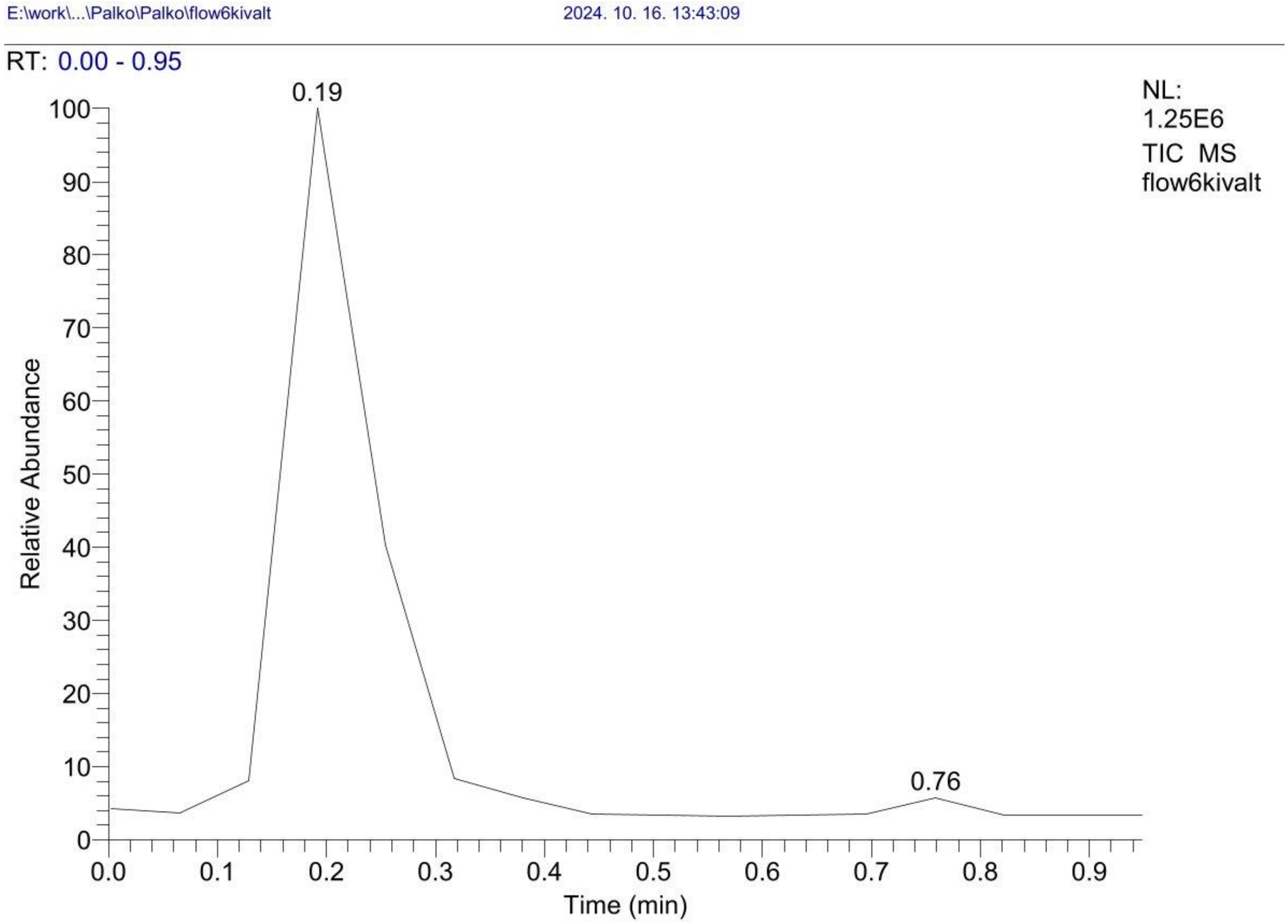

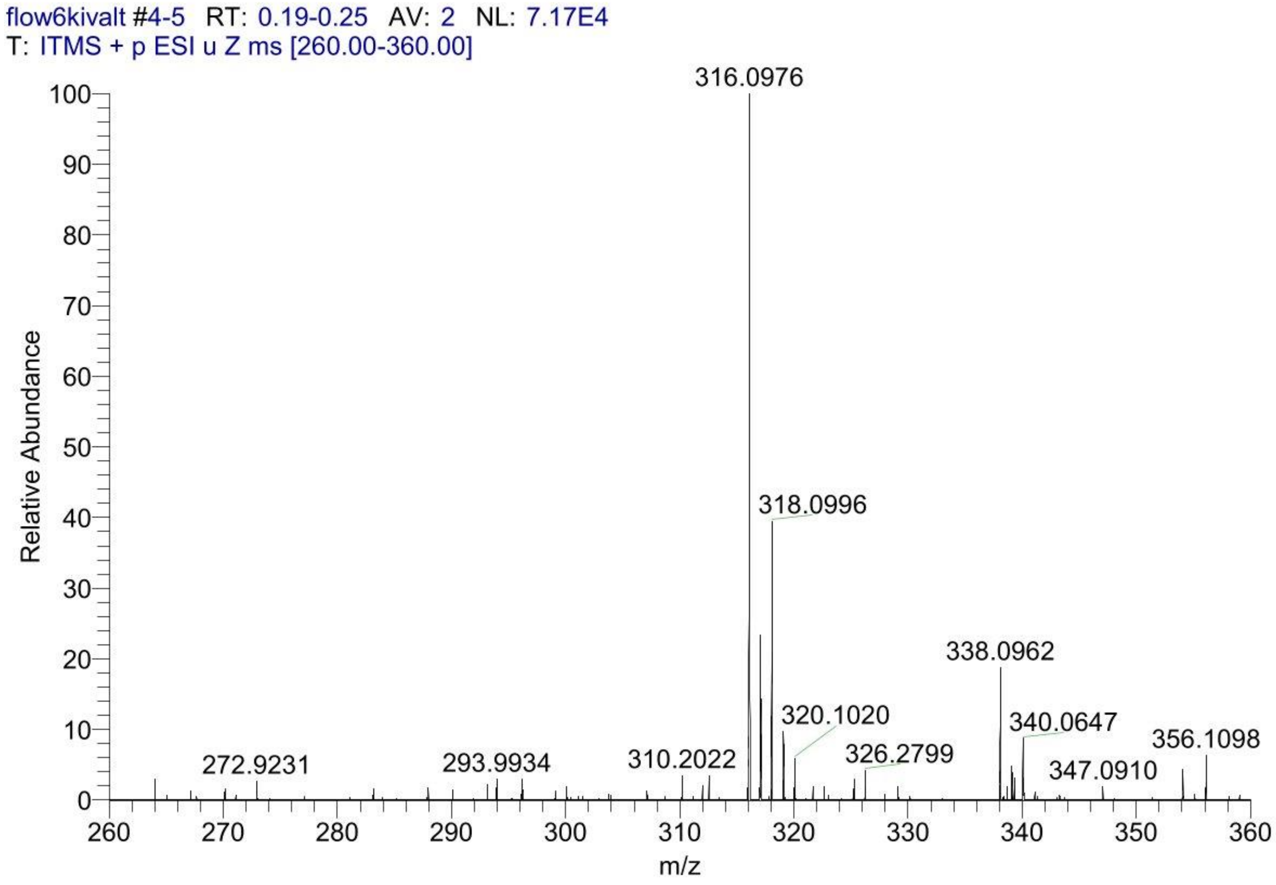

(2*S,4a*S**,5*R**,8*S**,8a*R**)-4a,5,8,8a-tetrahydro-1*H*-spiro[5,8-methanoquinazoline-2,3'-indoline]-2',4(3*H*)-dione (3e)**

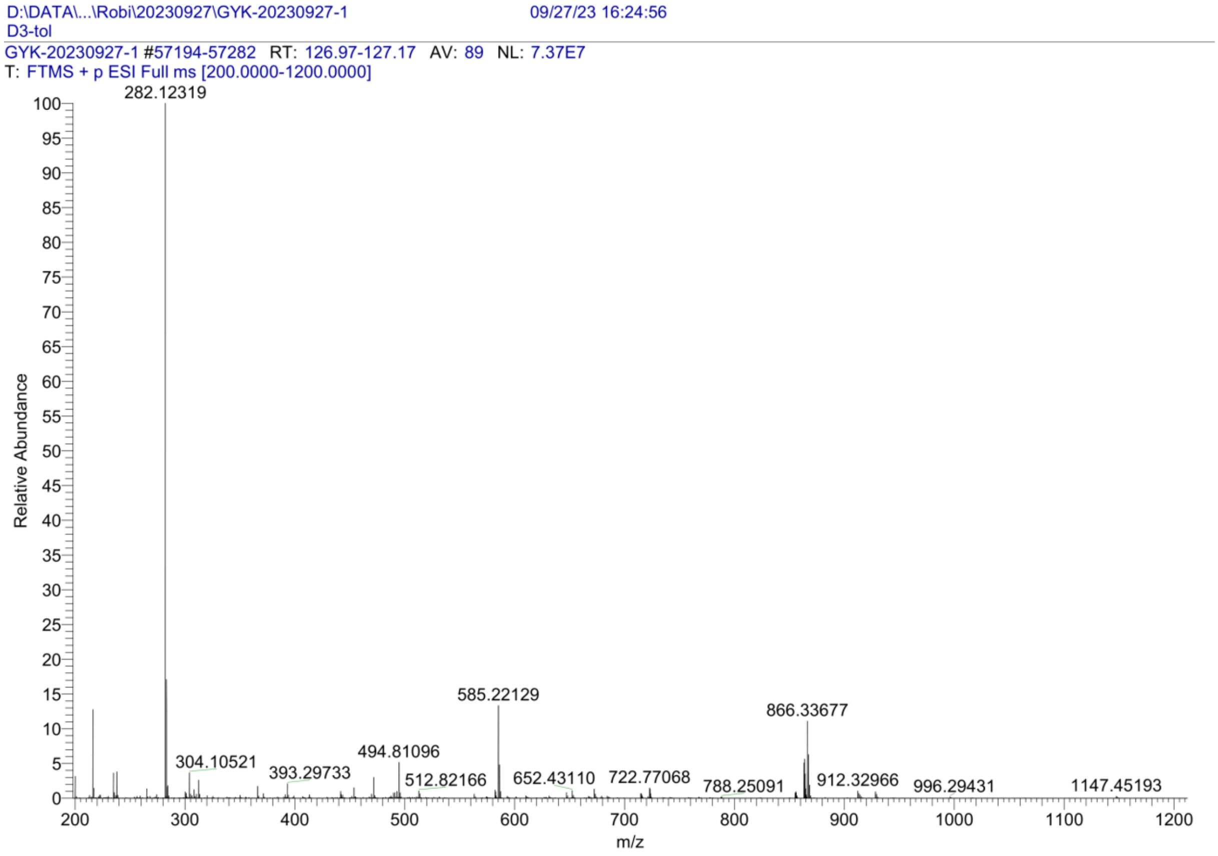

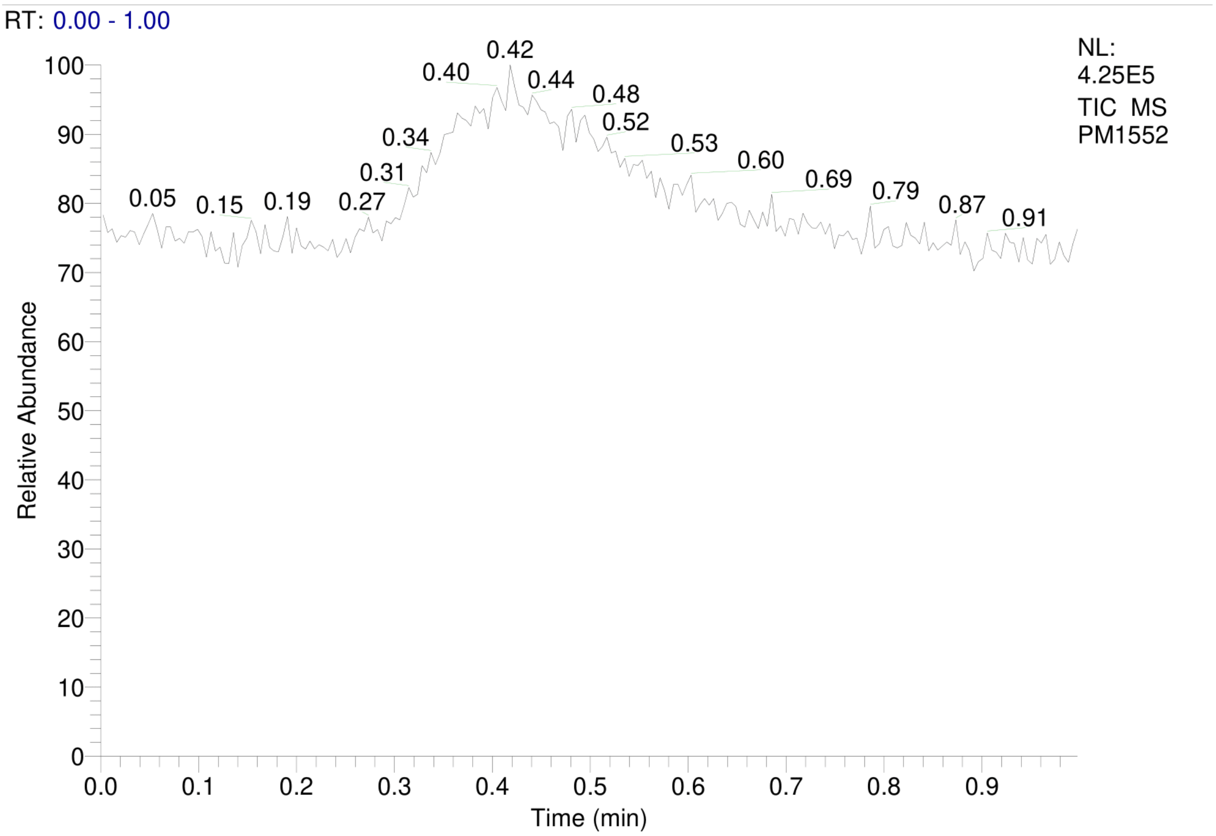
RT: 0.00 - 1.00
NL: 4.25E5
TIC MS PM1552
Relative Abundance
Time (min)
0.42
0.40
0.44
0.48
0.52
0.34
0.53
0.60
0.31
0.69
0.79
0.87
0.91
0.05
0.15
0.19
0.27

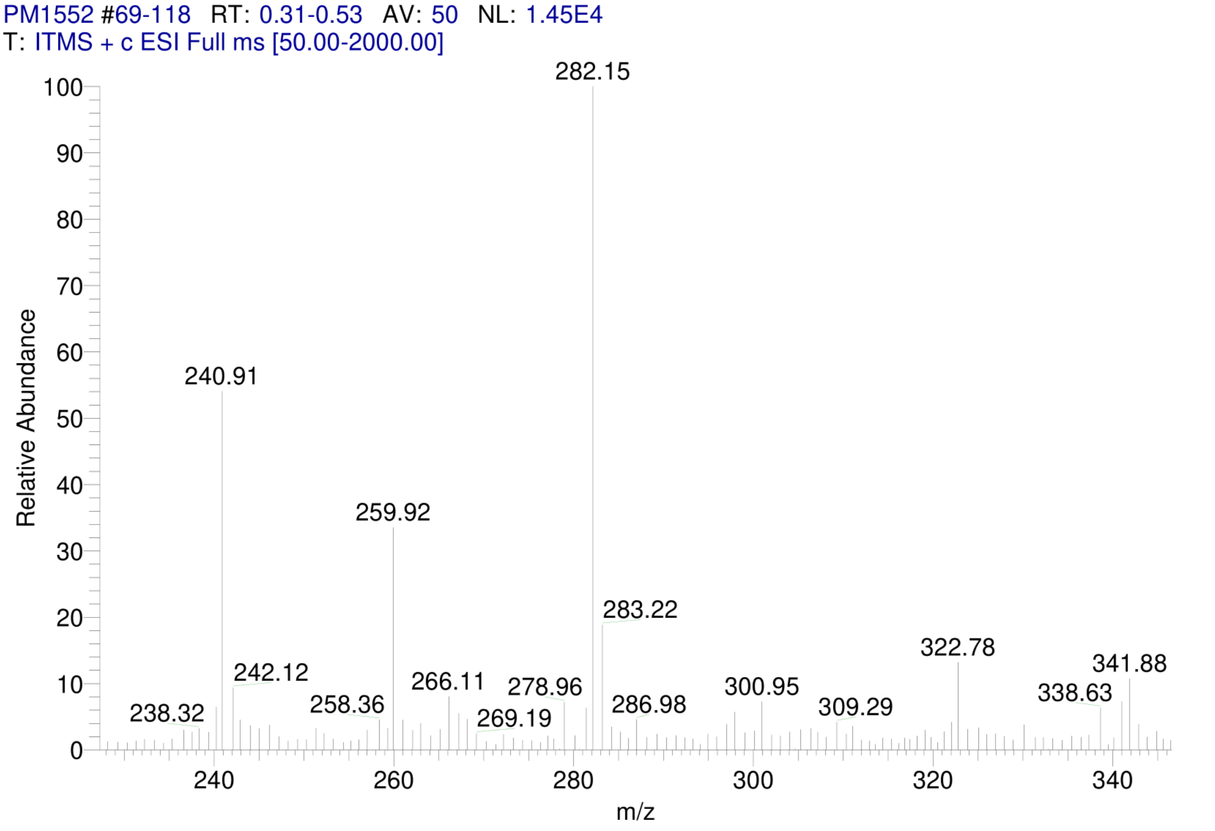
PM1552 #69-118 RT: 0.31-0.53 AV: 50 NL: 1.45E4
T: ITMS + c ESI Full ms [50.00-2000.00]
Relative Abundance
m/z
282.15
240.91
259.92
283.22
322.78
341.88
338.63
242.12
266.11
278.96
300.95
309.29
238.32
258.36
269.19
286.98

(2*S,4a*S**,5*R**,8*S**,8a*R**)-5'-methyl-4a,5,8,8a-tetrahydro-1*H*-spiro[5,8-methanoquinazoline-2,3'-indoline]-2',4(3*H*)-dione (3f)**

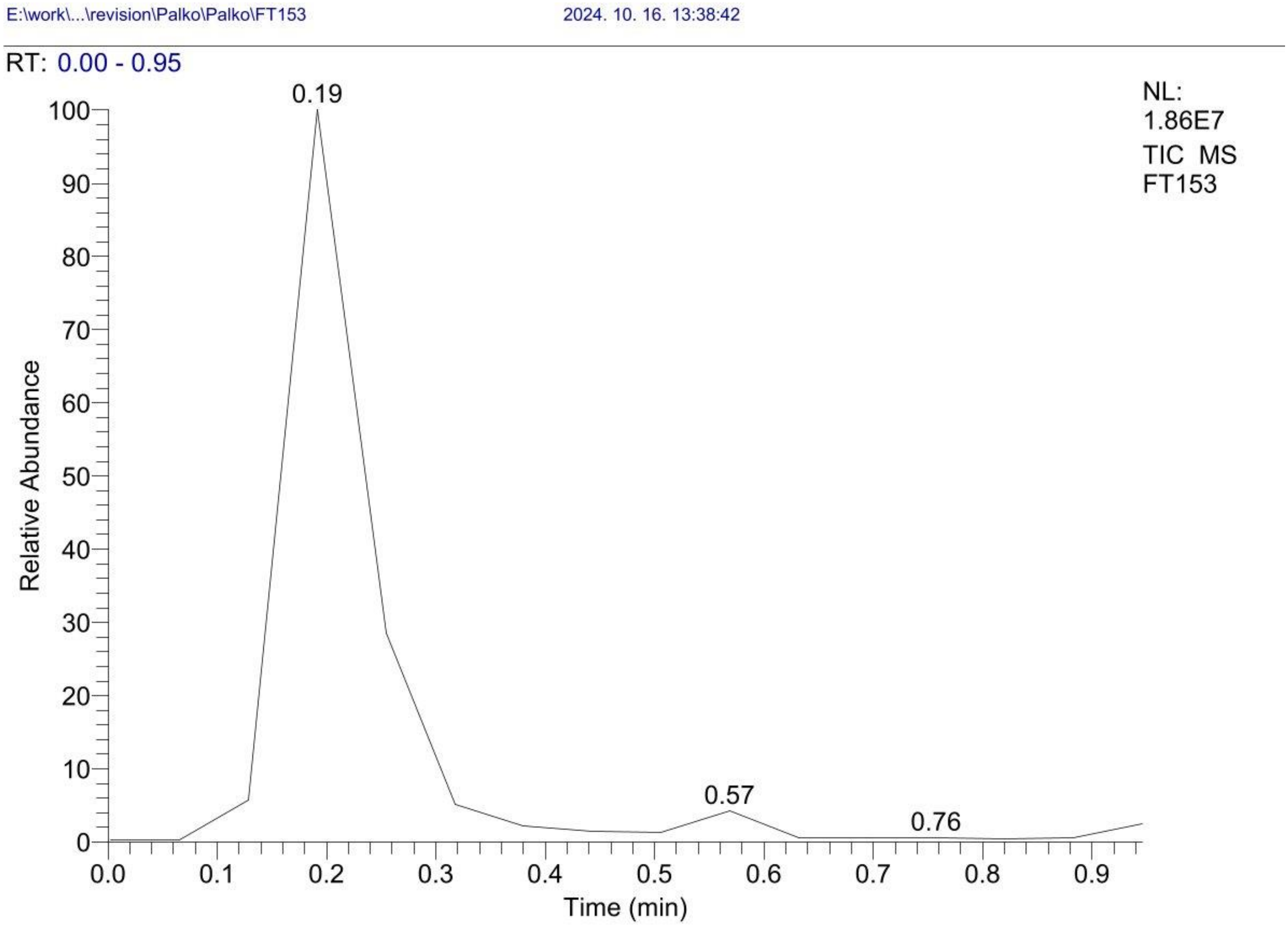

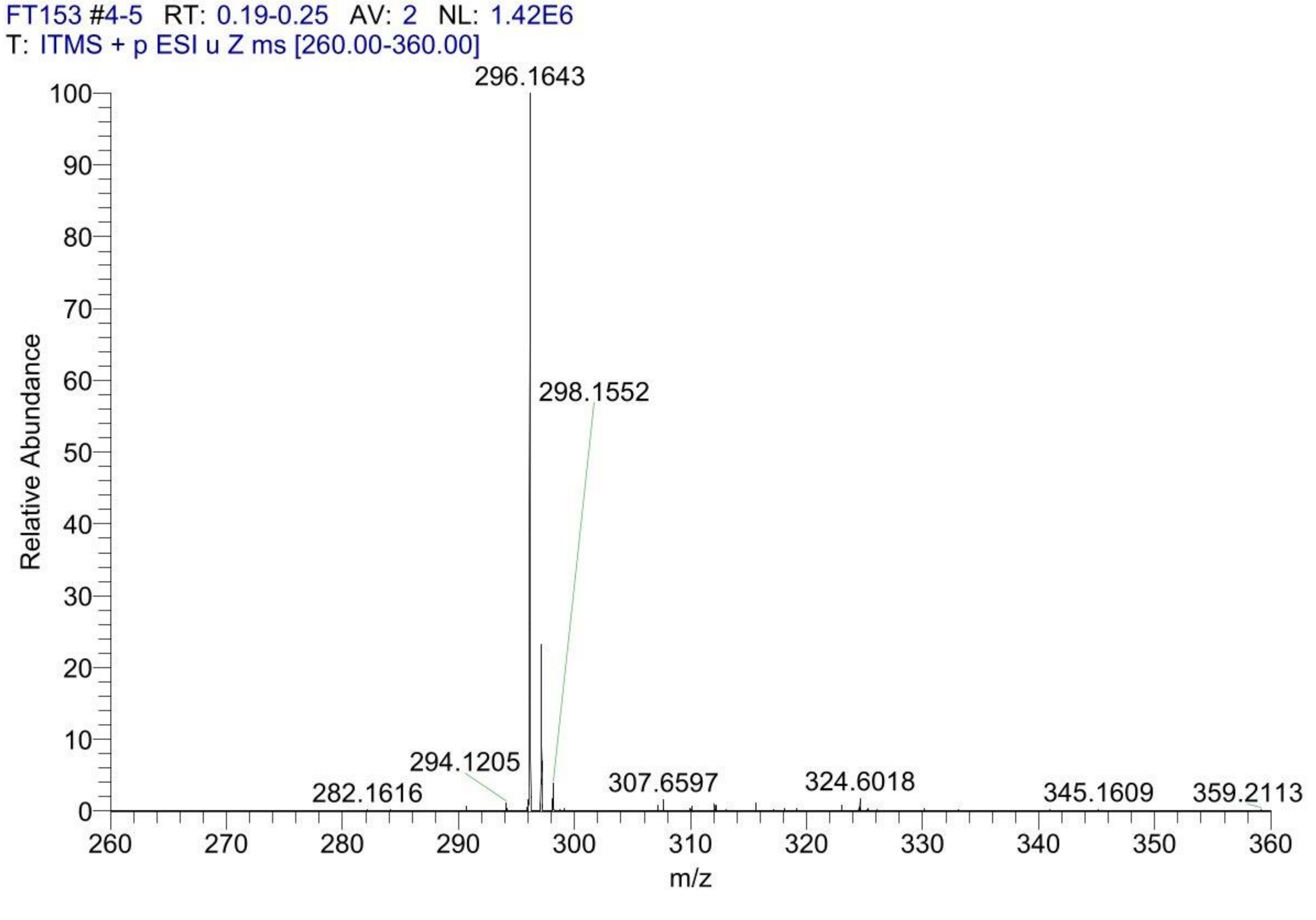

(2*S,4a*S**,5*R**,8*S**,8a*R**)-5'-iodo-4a,5,8,8a-tetrahydro-1*H*-spiro[5,8-methanoquinazoline-2,3'-indoline]-2',4(3*H*)-dione (3g)**

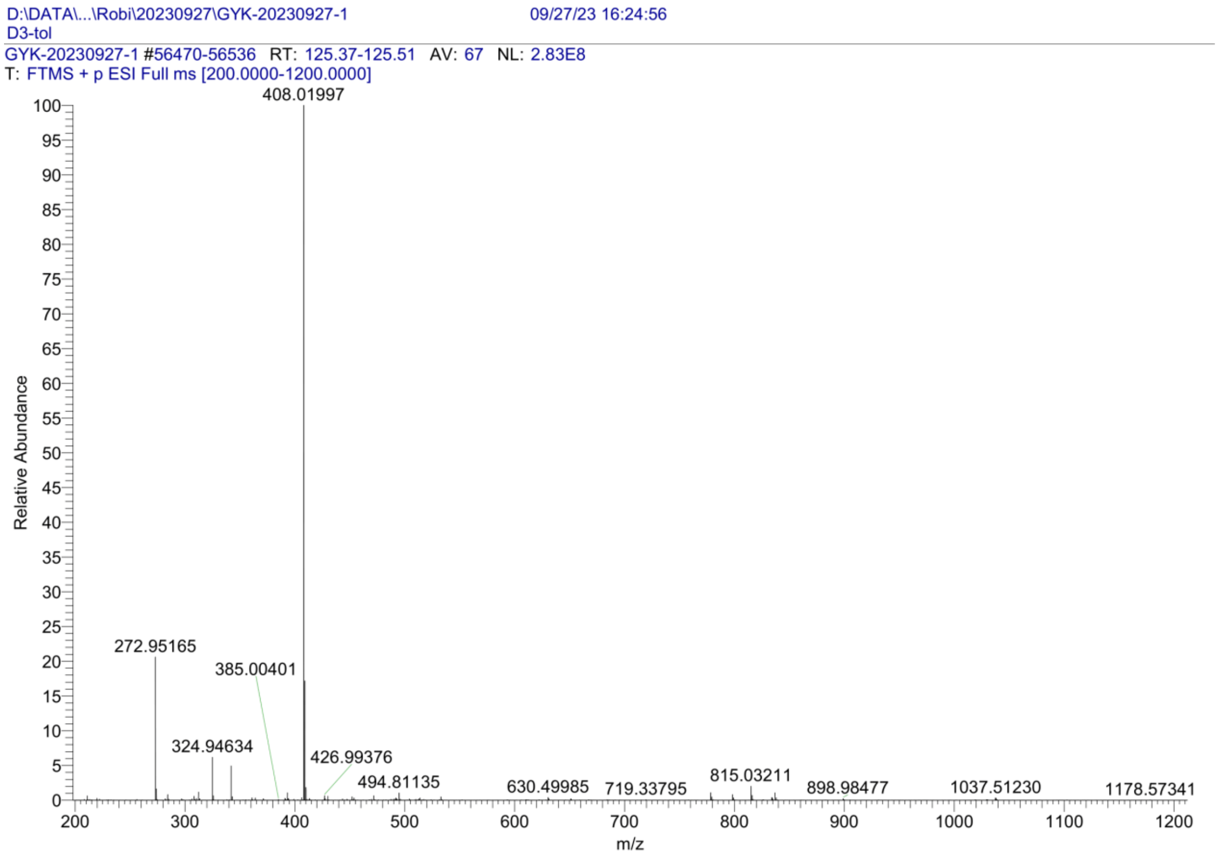

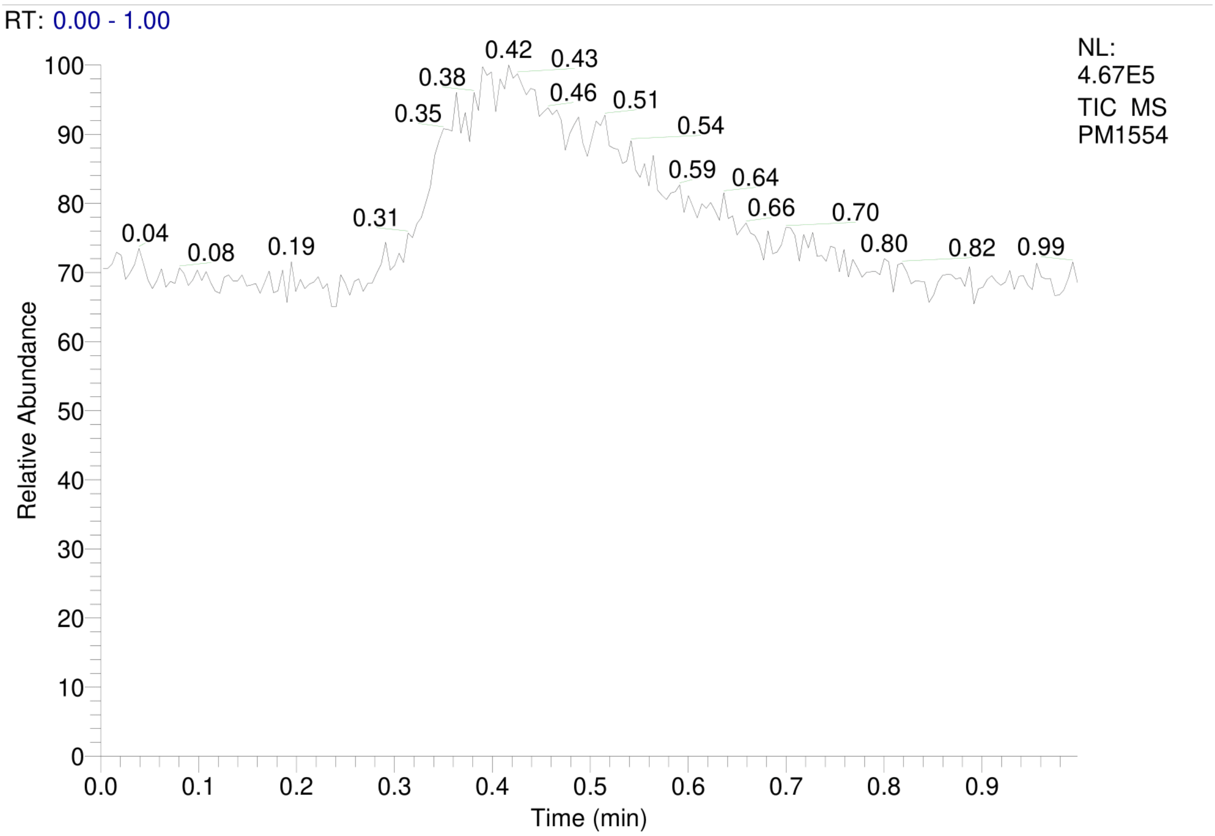
RT: 0.00 - 1.00
NL:
4.67E5
TIC MS
PM1554
Relative Abundance
Time (min)
0.42
0.43
0.38
0.35
0.46
0.51
0.54
0.59
0.64
0.66
0.70
0.31
0.04
0.08
0.19
0.80
0.82
0.99

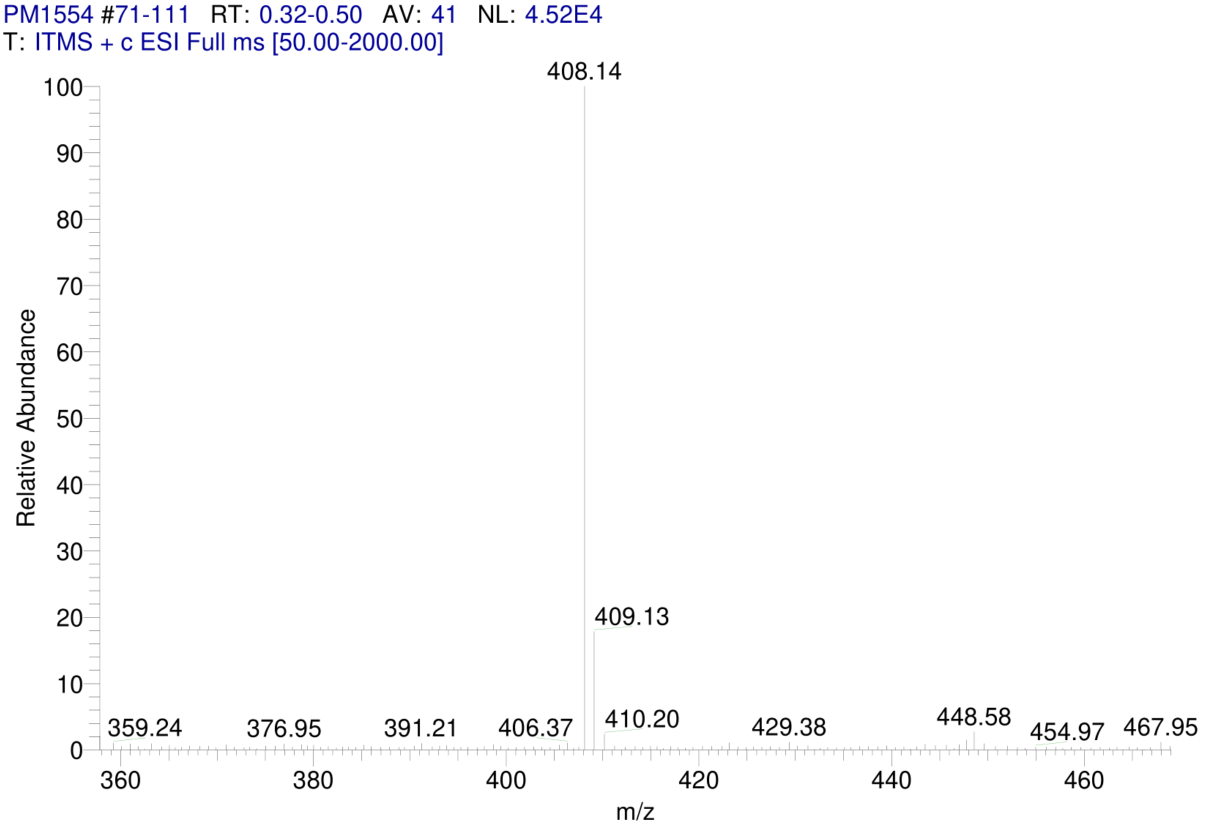
PM1554 #71-111 RT: 0.32-0.50 AV: 41 NL: 4.52E4
T: ITMS + c ESI Full ms [50.00-2000.00]
Relative Abundance
m/z
408.14
409.13
359.24
376.95
391.21
406.37
410.20
429.38
448.58
454.97
467.95

(4a*S,5*R**,8*S**,8a*R**)-7'-chloro-4a,5,8,8a-tetrahydro-1*H*-spiro[5,8-methanoquinazoline-2,3'-indoline]-2',4(3*H*)-dione (3h*)**

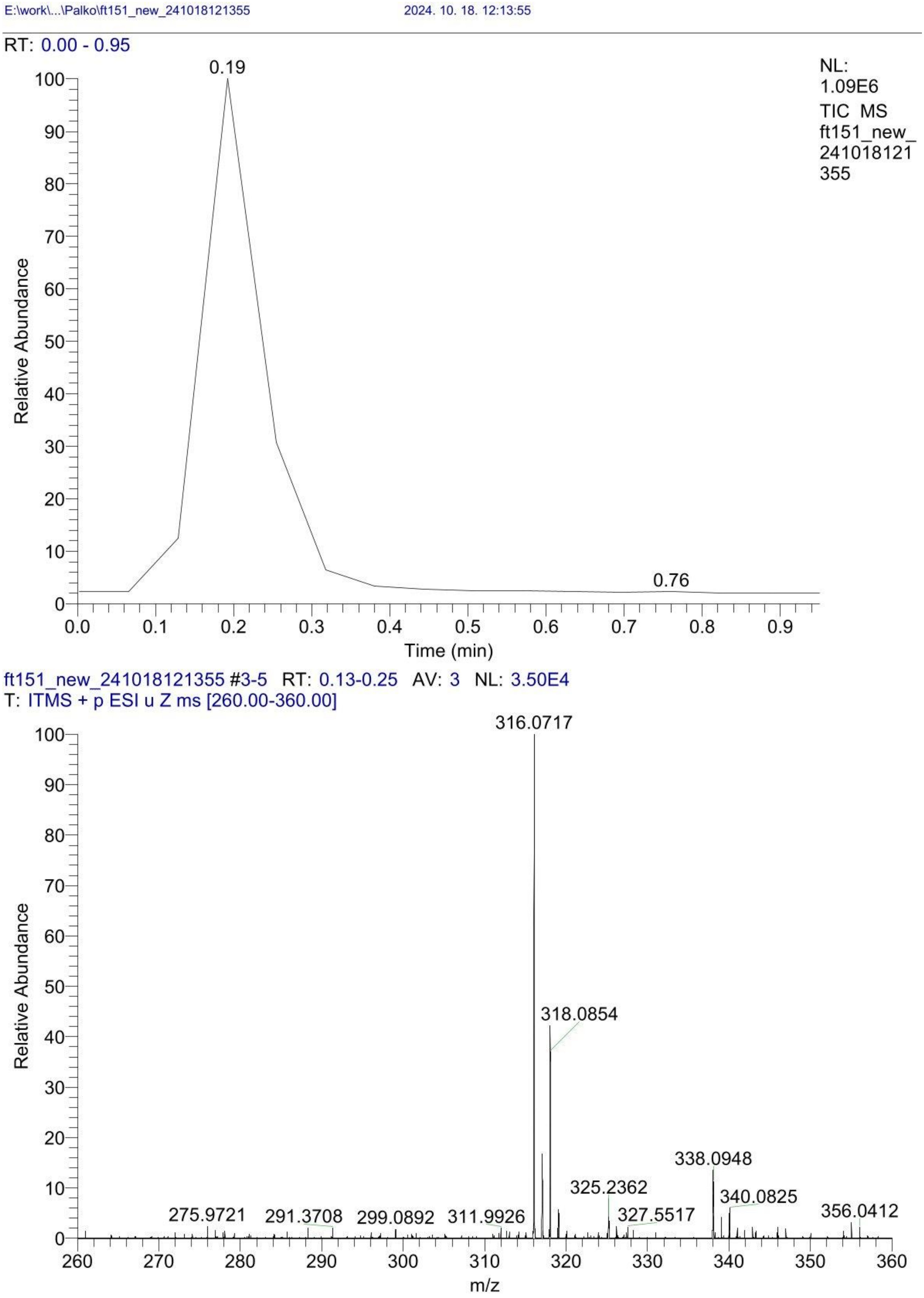

(2*R,4a*R**,5*R**,8*S**,8a*S**)-3-methyl-4a,5,8,8a-tetrahydro-1*H*-spiro[5,8-methanoquinazoline-2,3'-indoline]-2',4(3*H*)-dione (3i)**

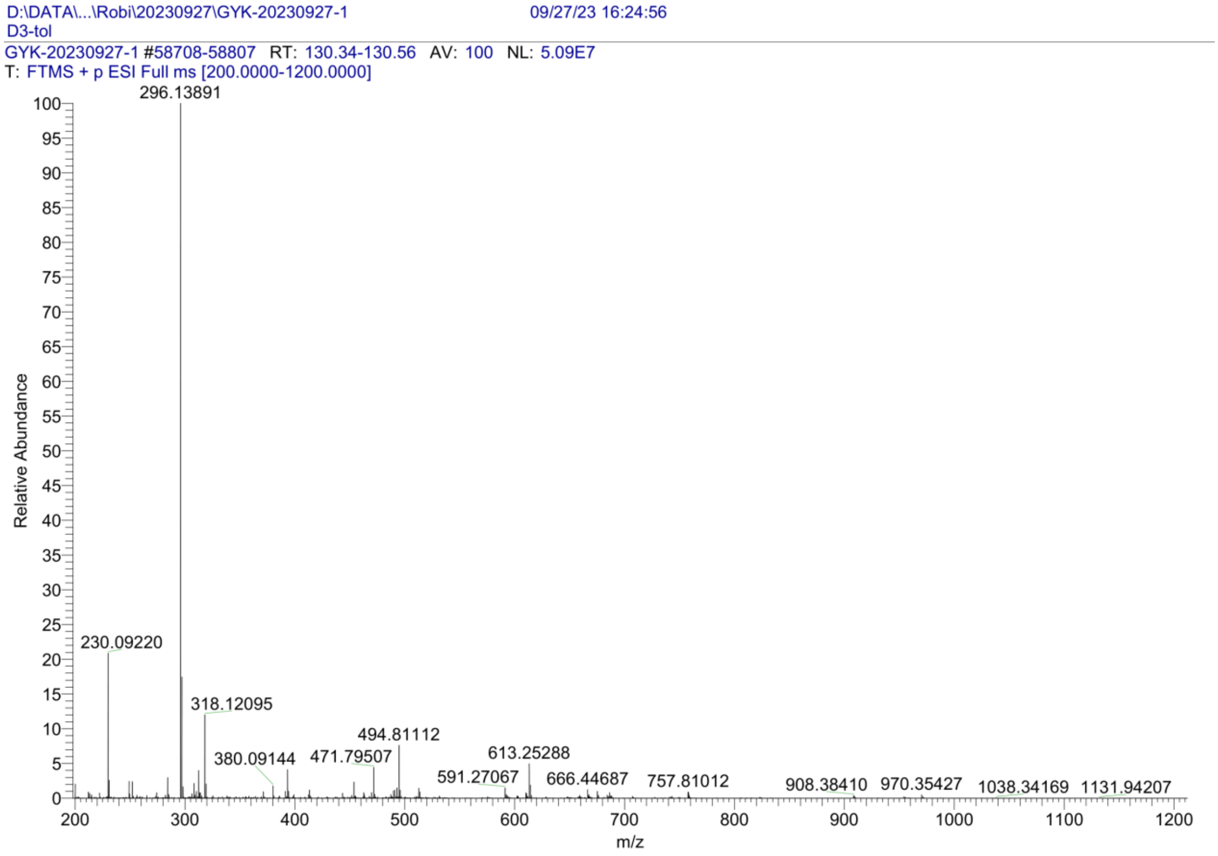

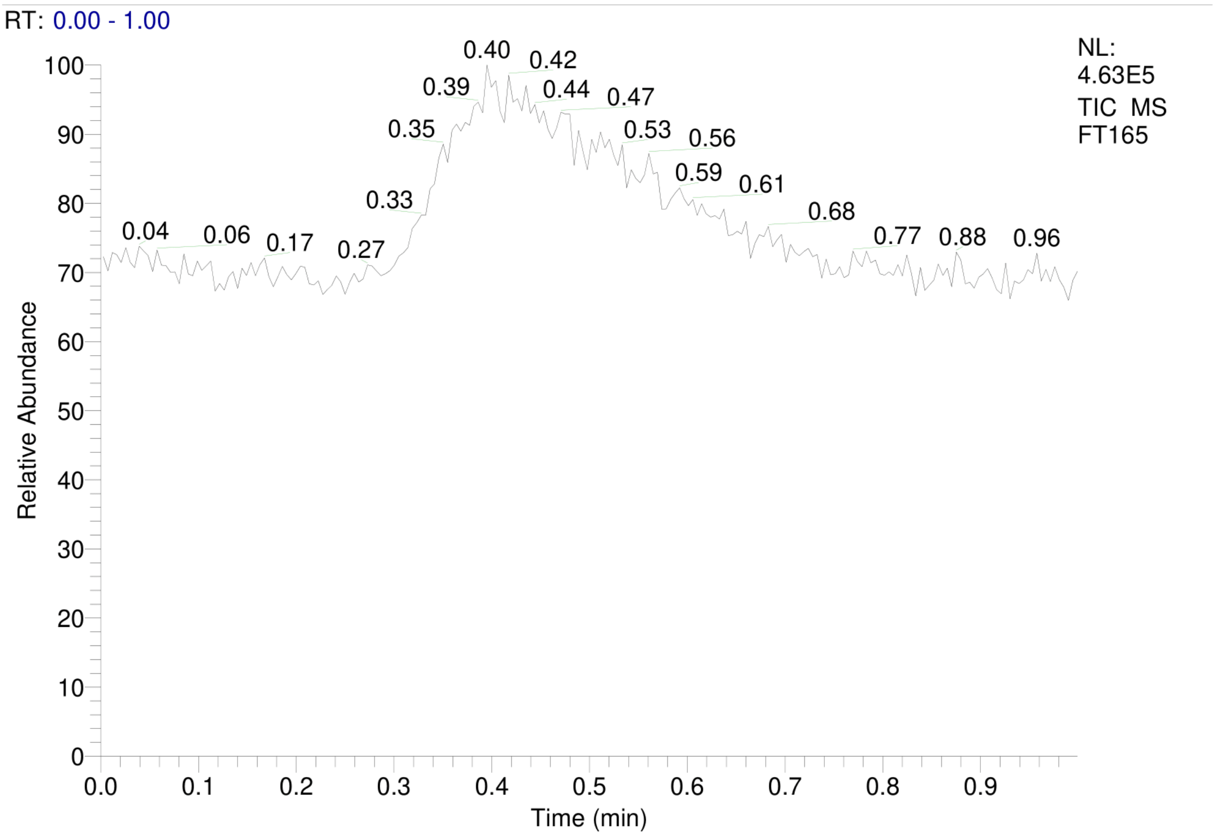
RT: 0.00 - 1.00
NL:
4.63E5
TIC MS
FT165
Relative Abundance
Time (min)
0.40
0.42
0.39
0.44
0.47
0.35
0.53
0.56
0.59
0.61
0.33
0.68
0.04
0.06
0.17
0.27
0.77
0.88
0.96

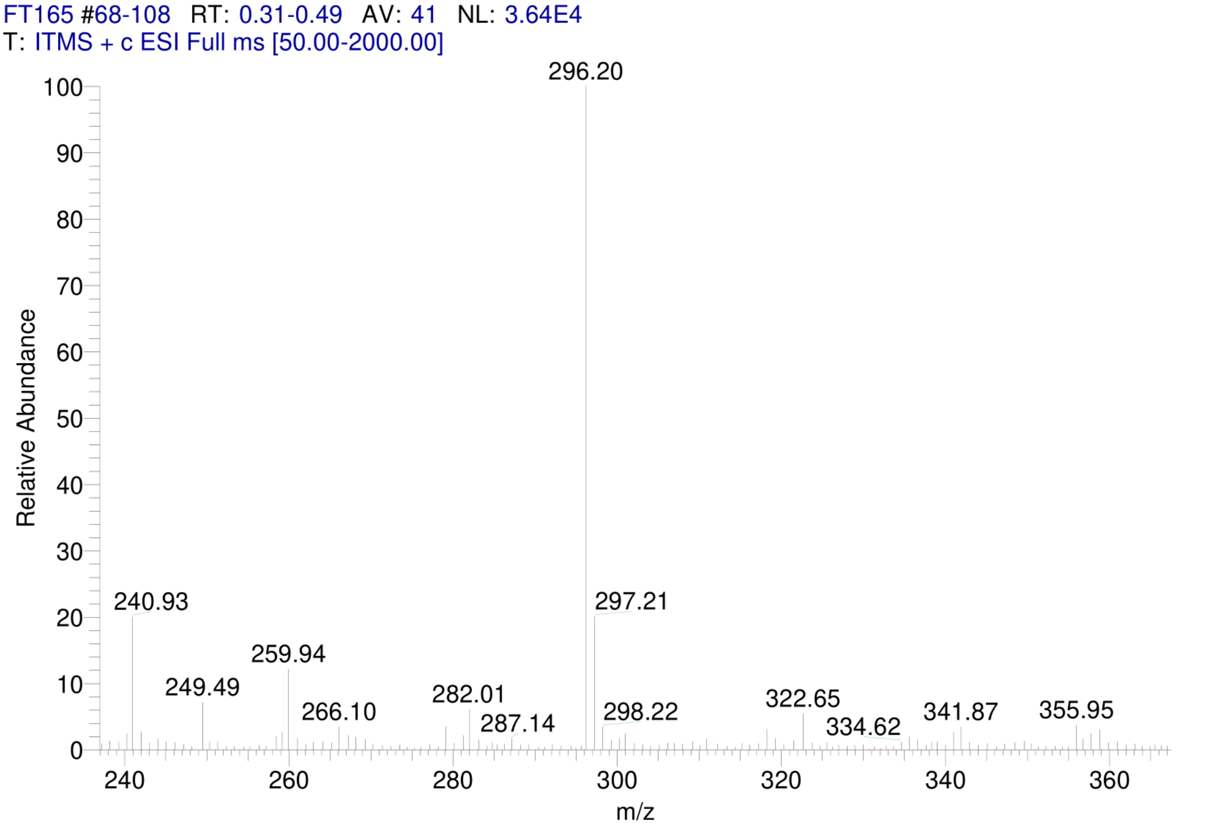
FT165 #68-108 RT: 0.31-0.49 AV: 41 NL: 3.64E4
T: ITMS + c ESI Full ms [50.00-2000.00]
Relative Abundance
m/z
296.20
240.93
297.21
259.94
249.49
266.10
282.01
287.14
298.22
322.65
334.62
341.87
355.95

(2*R,4a*R**,5*R**,8*S**,8a*S**)-3,5'-dimethyl-4a,5,8,8a-tetrahydro-1*H*-spiro[5,8-methanoquinazoline-2,3'-indoline]-2',4(3*H*)-dione (3j)**

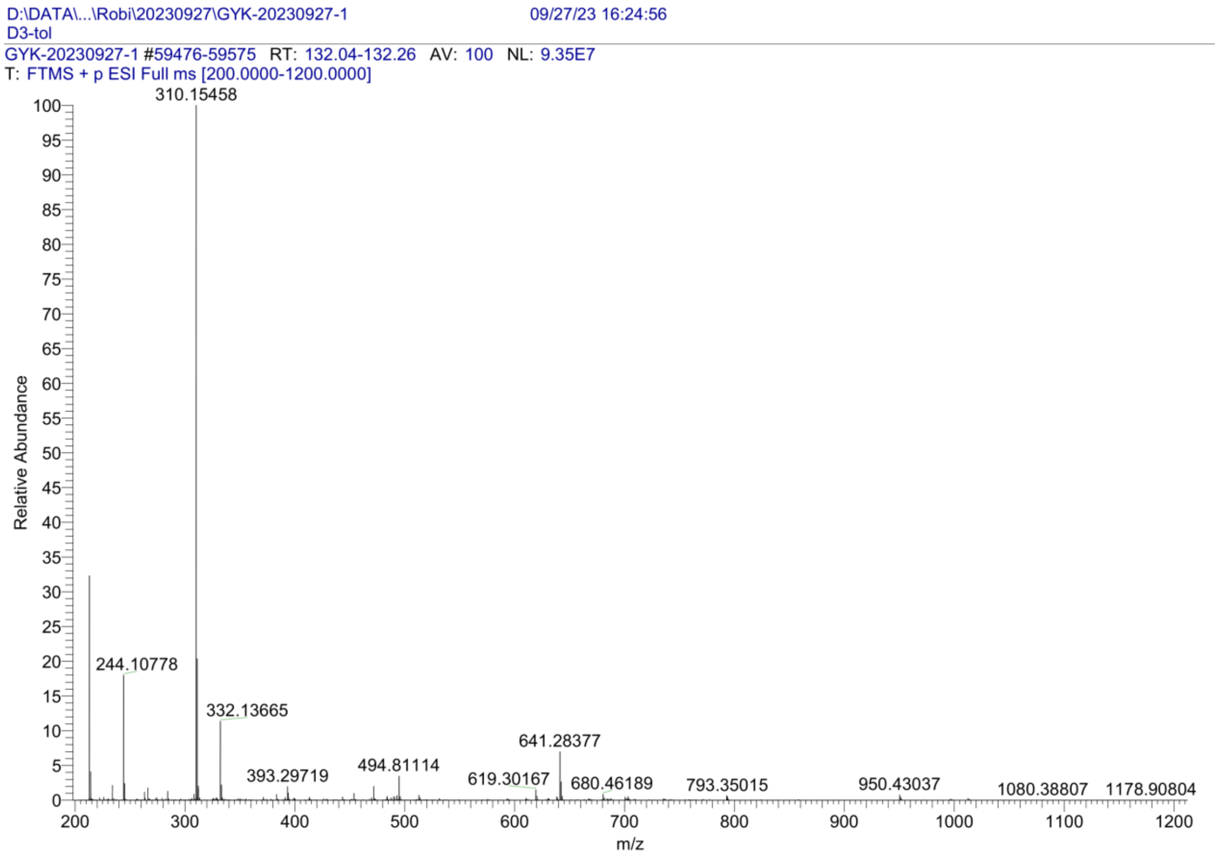

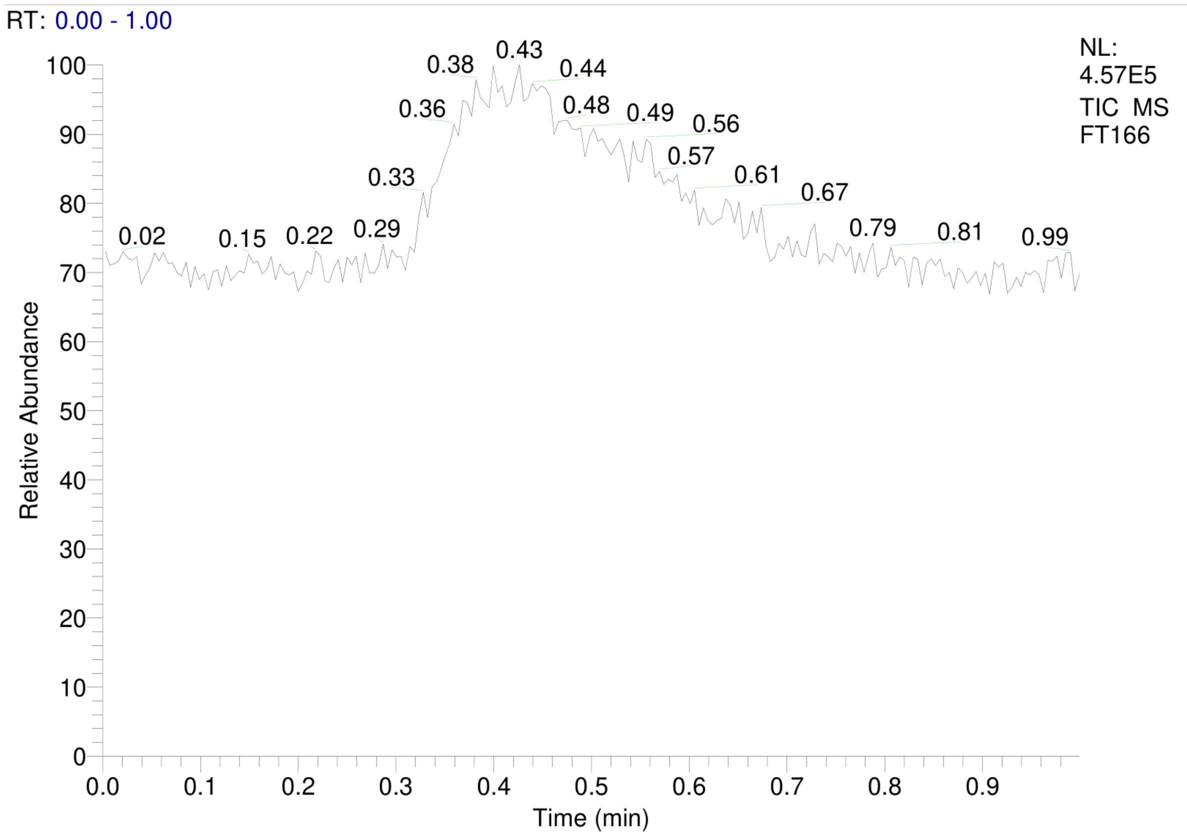
RT: 0.00 - 1.00
NL: 4.57E5
TIC MS FT166
0.02
0.15
0.22
0.29
0.33
0.36
0.38
0.43
0.44
0.48
0.49
0.56
0.57
0.61
0.67
0.79
0.81
0.99
Relative Abundance
Time (min)

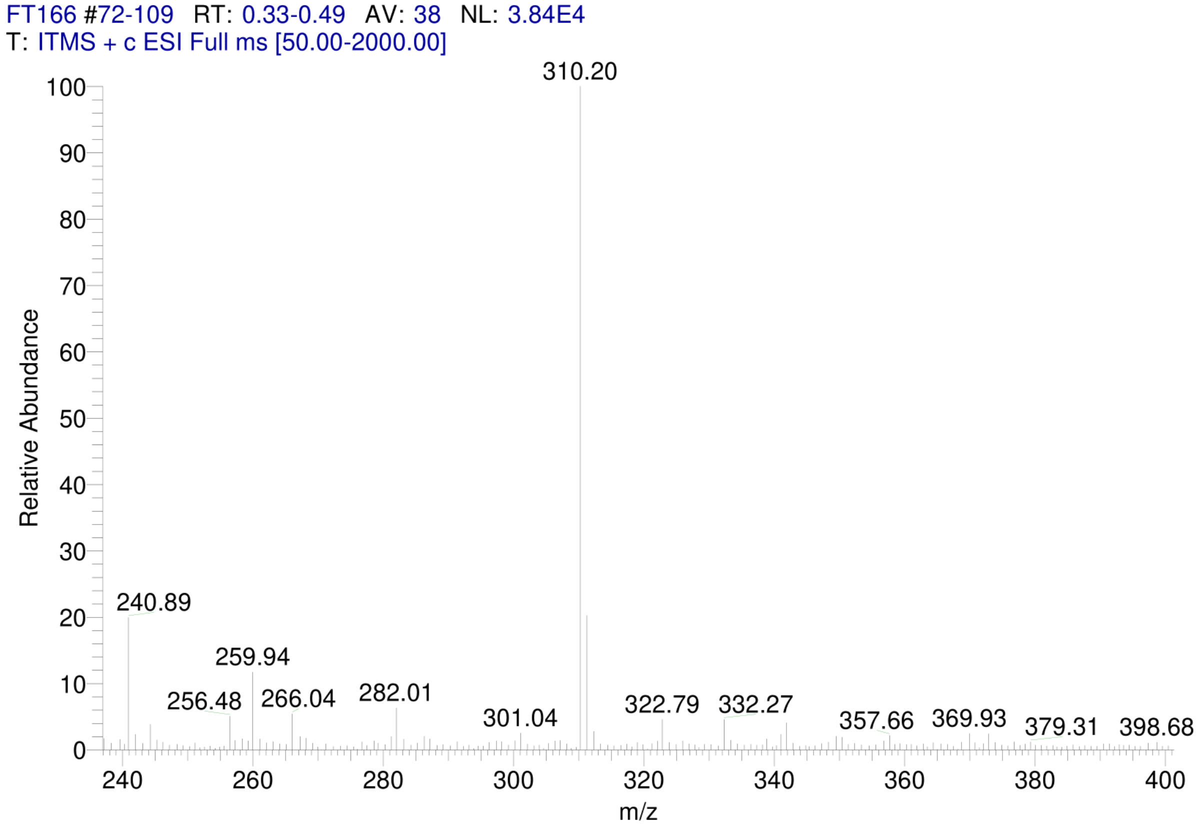
FT166 #72-109 RT: 0.33-0.49 AV: 38 NL: 3.84E4
T: ITMS + c ESI Full ms [50.00-2000.00]
310.20
240.89
259.94
256.48
266.04
282.01
301.04
322.79
332.27
357.66
369.93
379.31
398.68
Relative Abundance
m/z

(2*R,4a*R**,5*R**,8*S**,8a*S**)-5'-iodo-3-methyl-4a,5,8,8a-tetrahydro-1*H*-spiro[5,8-methanoquinazoline-2,3'-indoline]-2',4(3*H*)-dione (3k)**

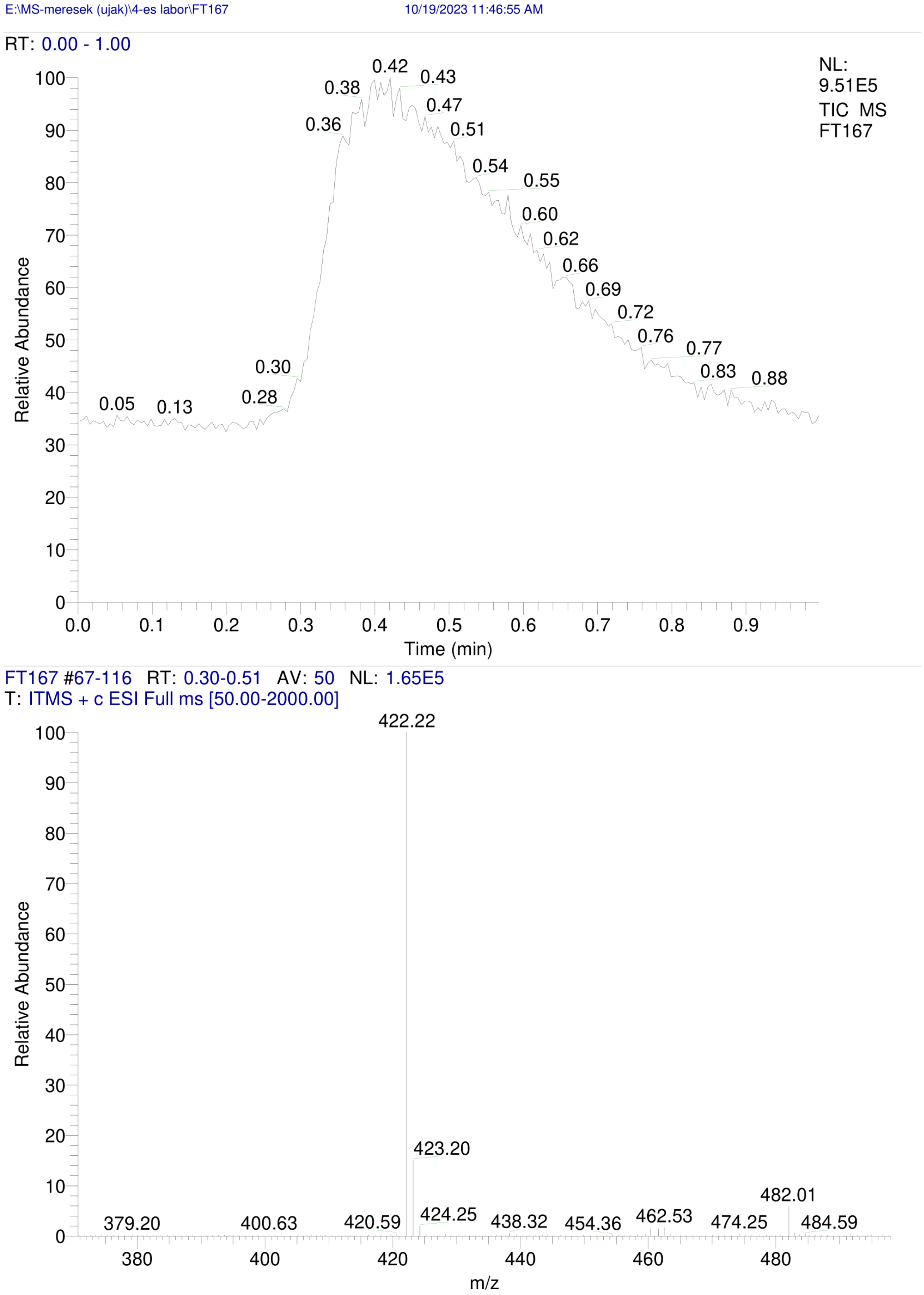

(2*R,4a*R**,5*R**,8*S**,8a*S**)-7'-chloro-3-methyl-4a,5,8,8a-tetrahydro-1*H*-spiro[5,8-methanoquinazoline-2,3'-indoline]-2',4(3*H*)-dione (3l*)**

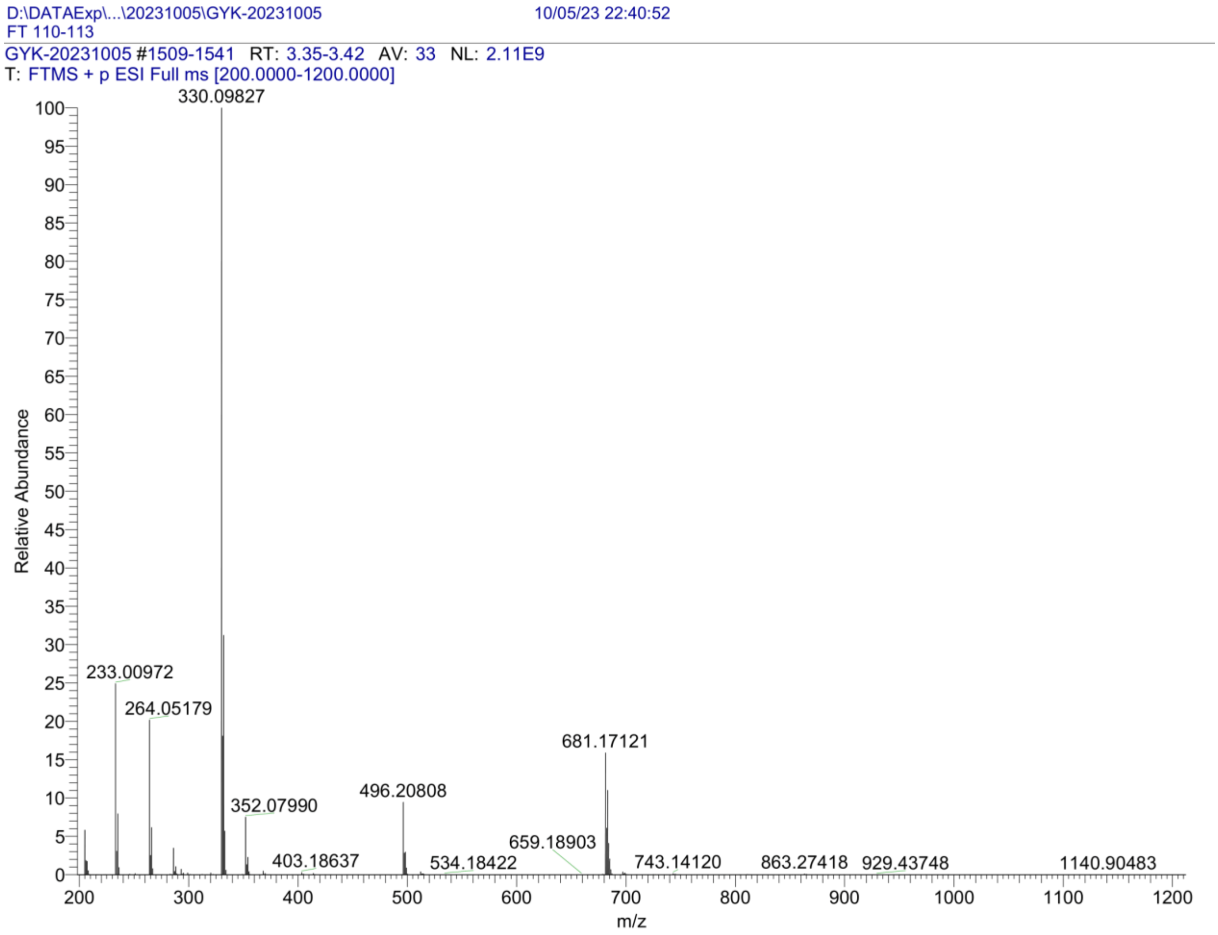

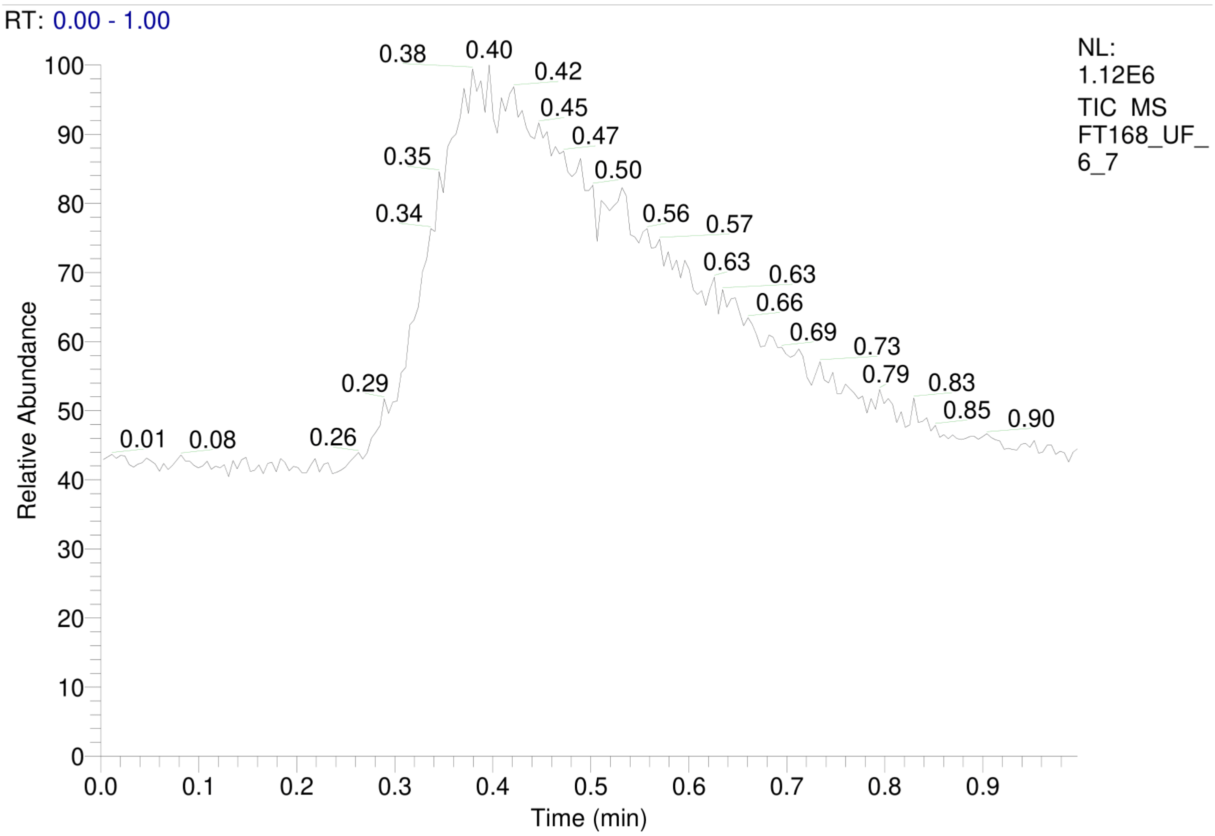
RT: 0.00 - 1.00
NL: 1.12E6
TIC MS FT168_UF_6_7
Relative Abundance
Time (min)
0.38
0.40
0.42
0.45
0.47
0.50
0.35
0.34
0.56
0.57
0.63
0.63
0.66
0.69
0.73
0.79
0.83
0.85
0.90
0.29
0.26
0.01
0.08

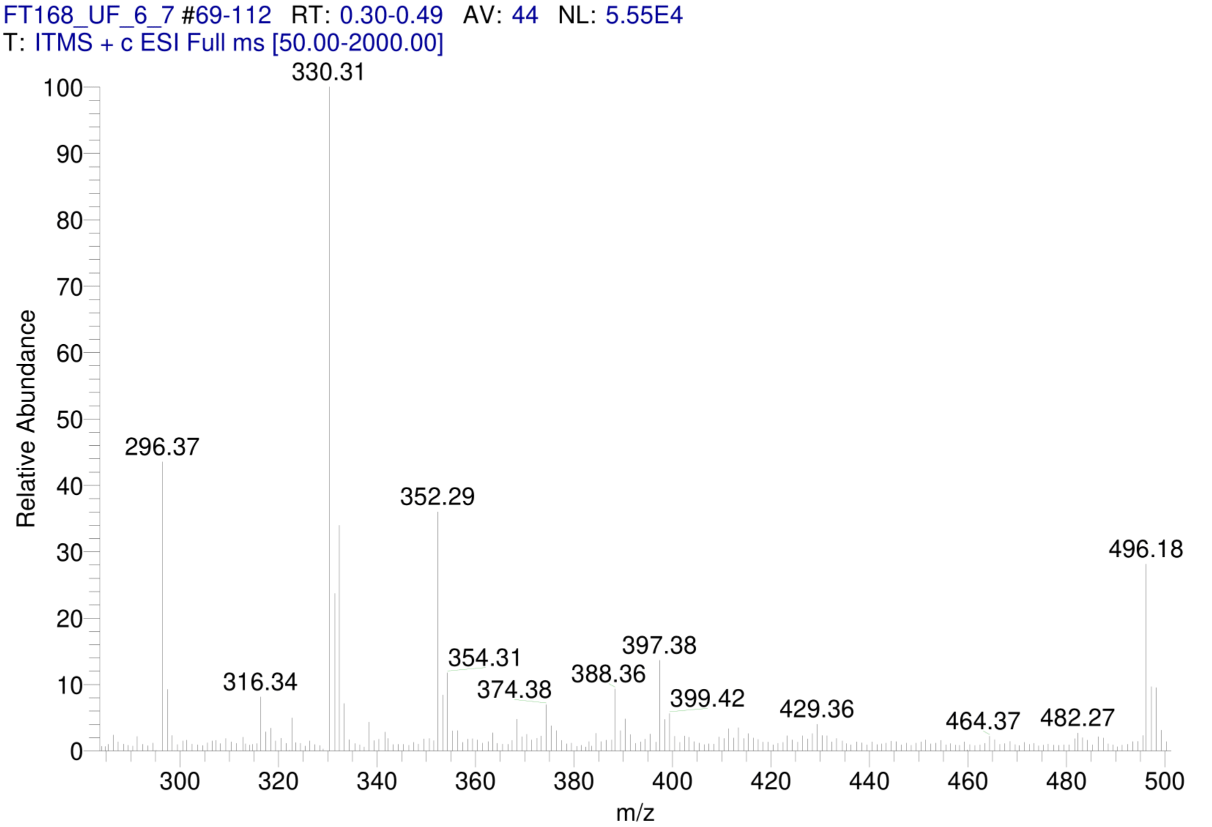
FT168_UF_6_7 #69-112 RT: 0.30-0.49 AV: 44 NL: 5.55E4
T: ITMS + c ESI Full ms [50.00-2000.00]
Relative Abundance
m/z
330.31
296.37
352.29
496.18
316.34
354.31
374.38
388.36
397.38
399.42
429.36
464.37
482.27

(2*S,4a*S**,5*R**,8*S**,8a*R**)-3-methyl-4a,5,8,8a-tetrahydro-1*H*-spiro[5,8-methanoquinazoline-2,3'-indoline]-2',4(3*H*)-dione (3m)**

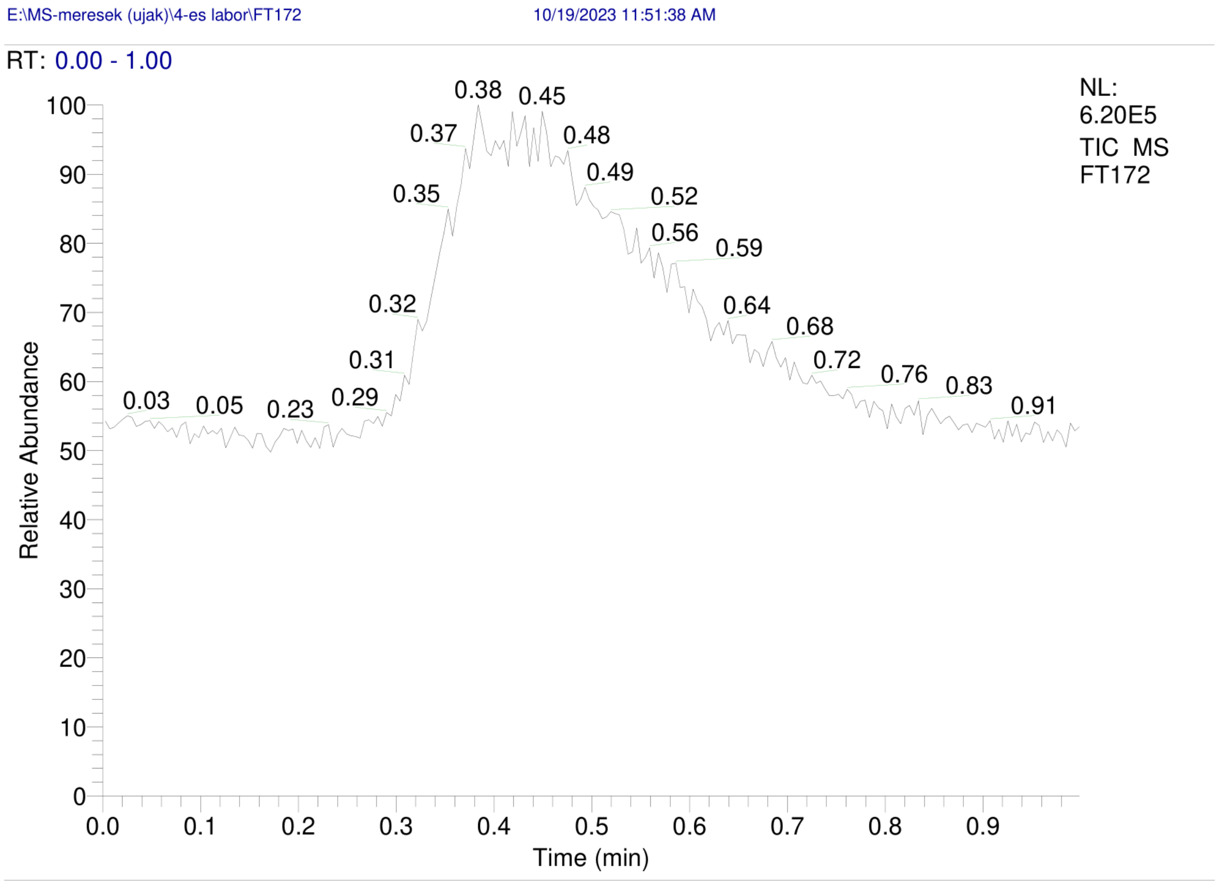

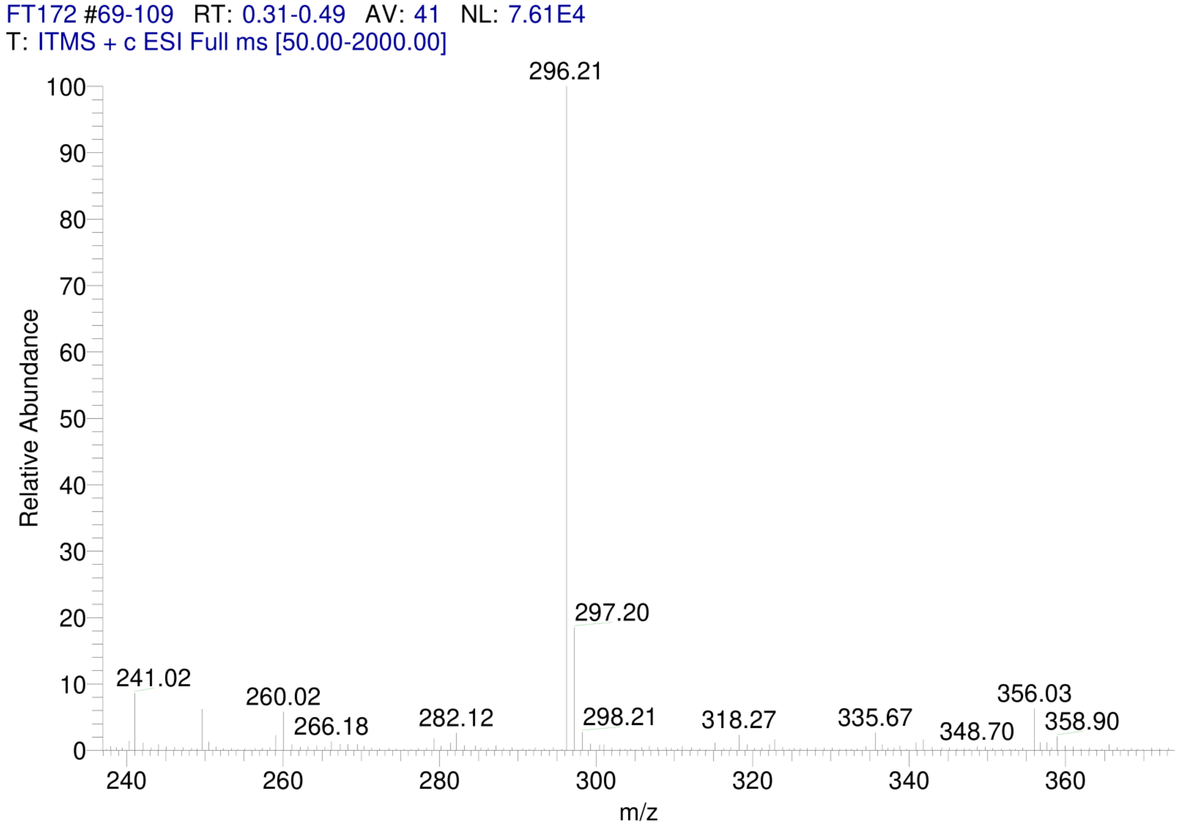

(4a*S,5*R**,8*S**,8a*R**)-3,5'-dimethyl-4a,5,8,8a-tetrahydro-1*H*-spiro[5,8-methanoquinazoline-2,3'-indoline]-2',4(*3H*)-dione (3n*)**

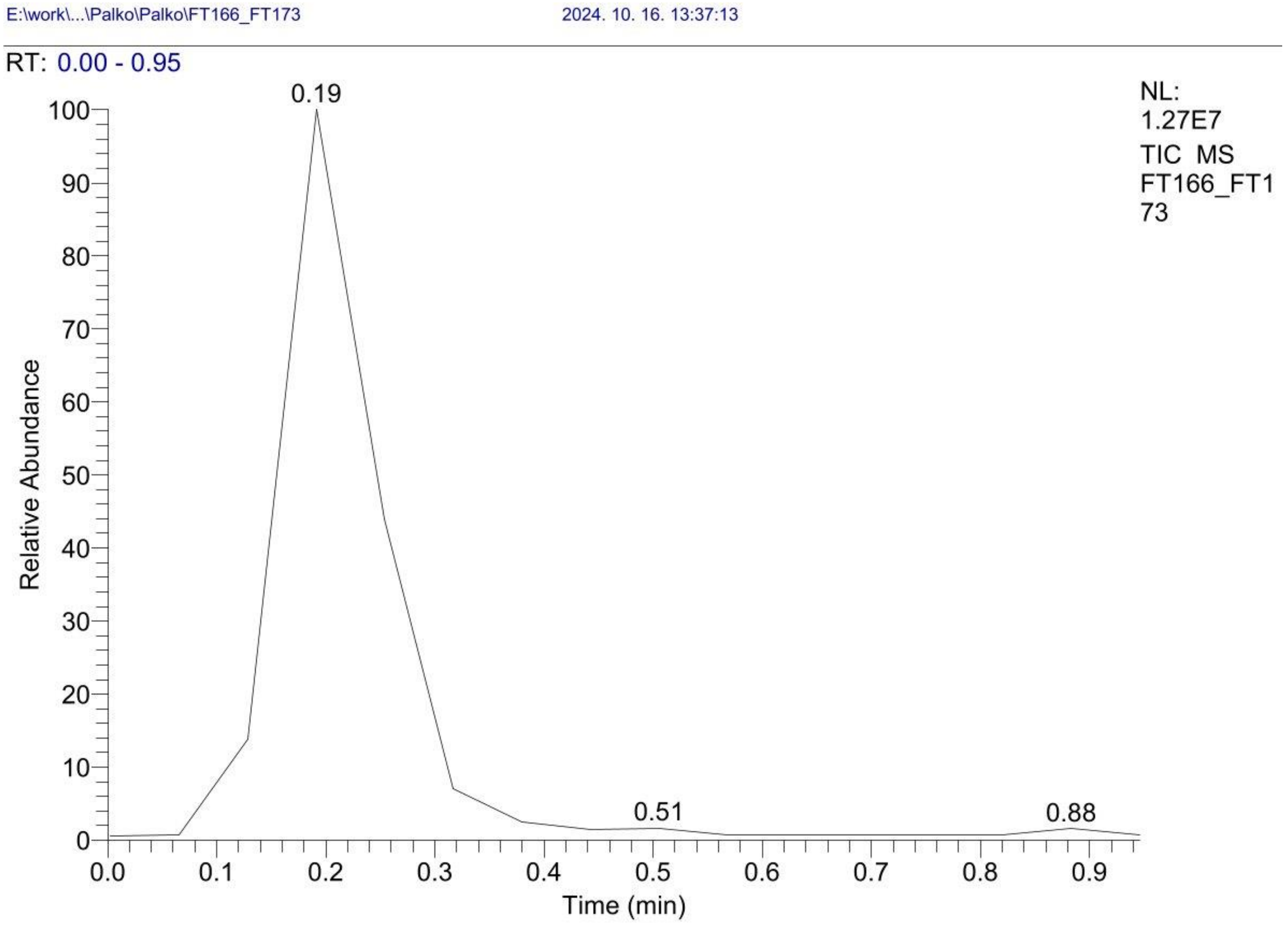

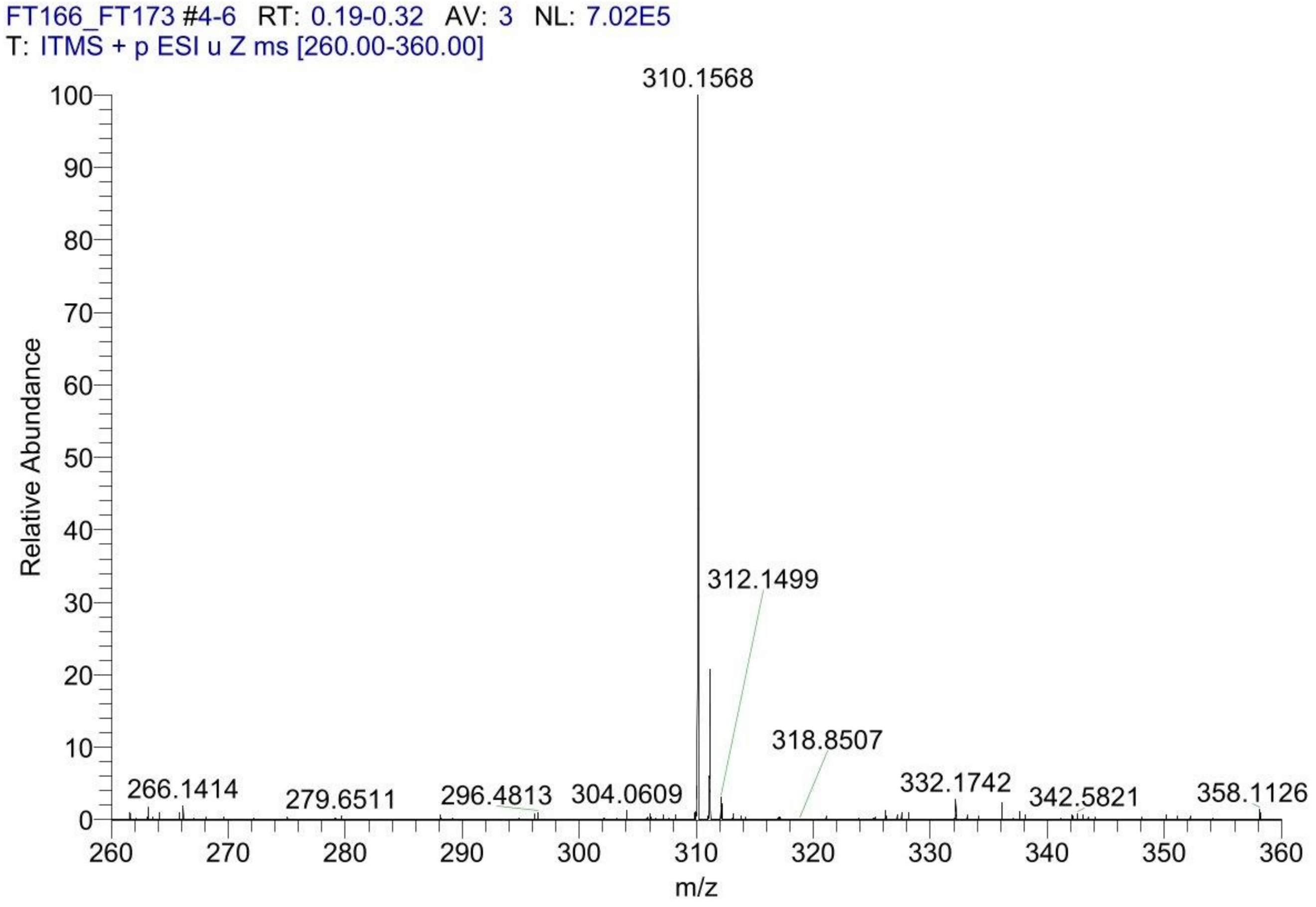

(4a*S,5*R**,8*S**,8a*R**)-5'-iodo-3-methyl-4a,5,8,8a-tetrahydro-1*H*-spiro[5,8-methanoquinazoline-2,3'-indoline]-2',4(3*H*)-dione (3o*)**

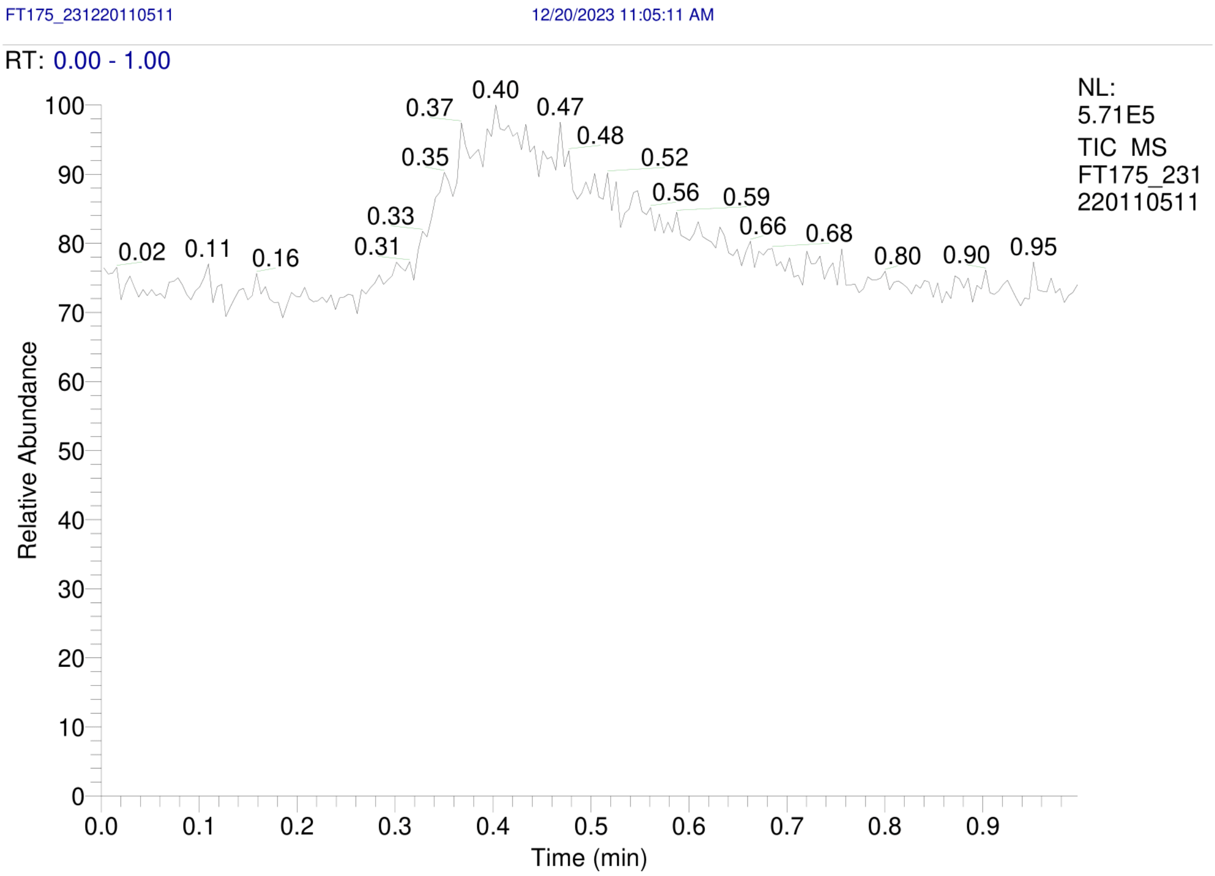

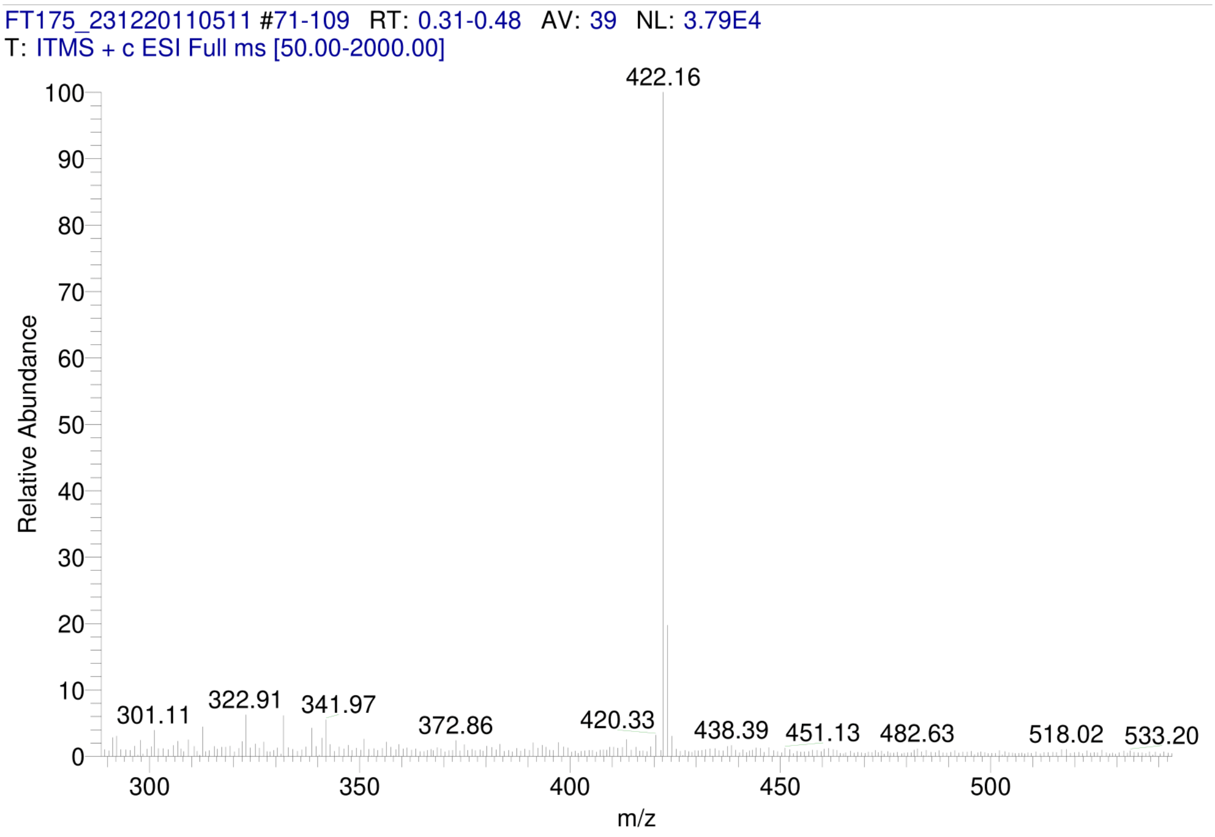

(4aS*,5R*,8S*,8aR*)-7'-chloro-3-methyl-4a,5,8,8a-tetrahydro-1*H*-spiro[5,8-methanoquinazoline-2,3'-indoline]-2',4(3*H*)-dione (3p*)

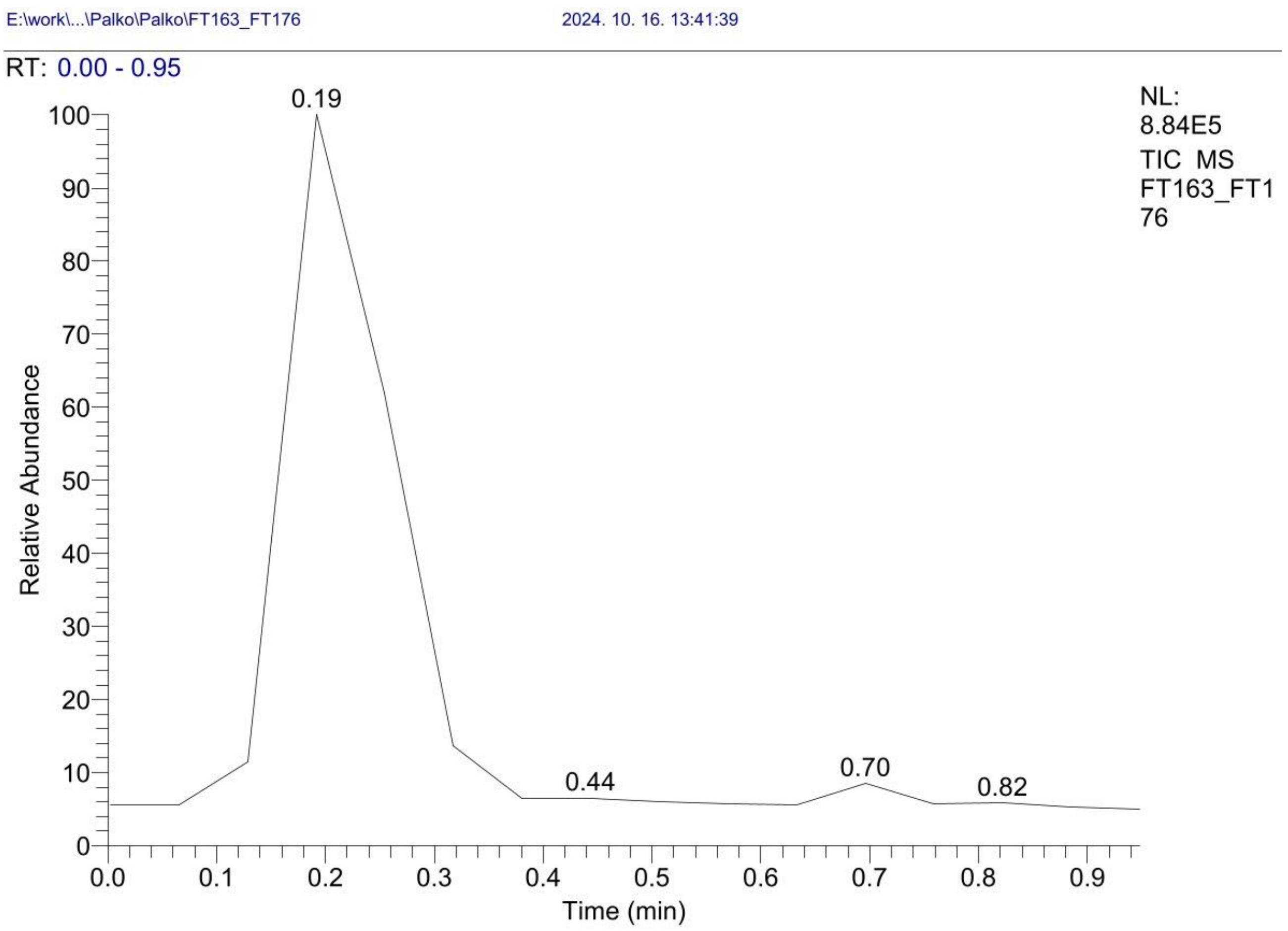

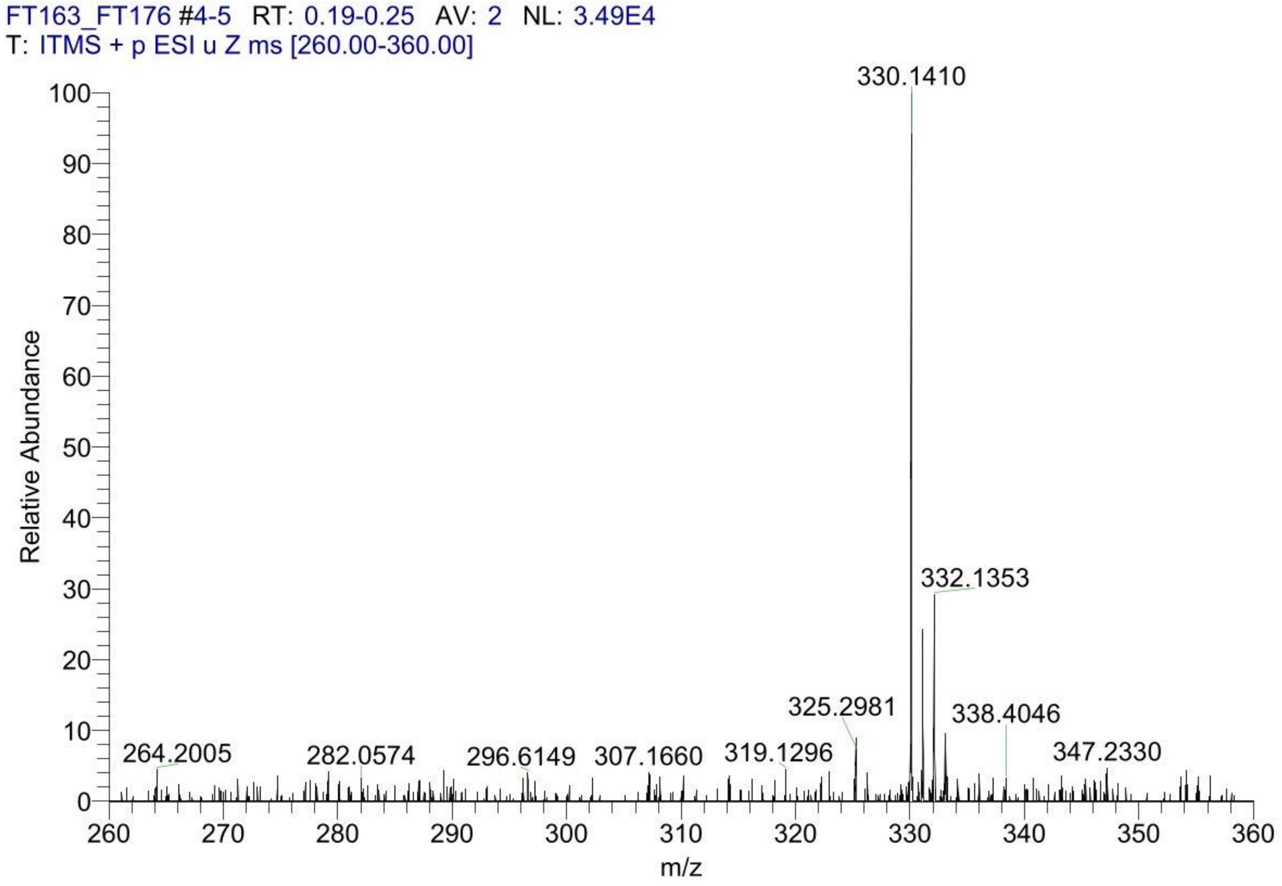

(1*R,2*R**,3*S**,4*S**)-3-((*Z*)-(5-methyl-2-oxoindolin-3-ylidene)amino)bicyclo[2.2.1]hept-5-ene-2-carboxamide (Bb)**

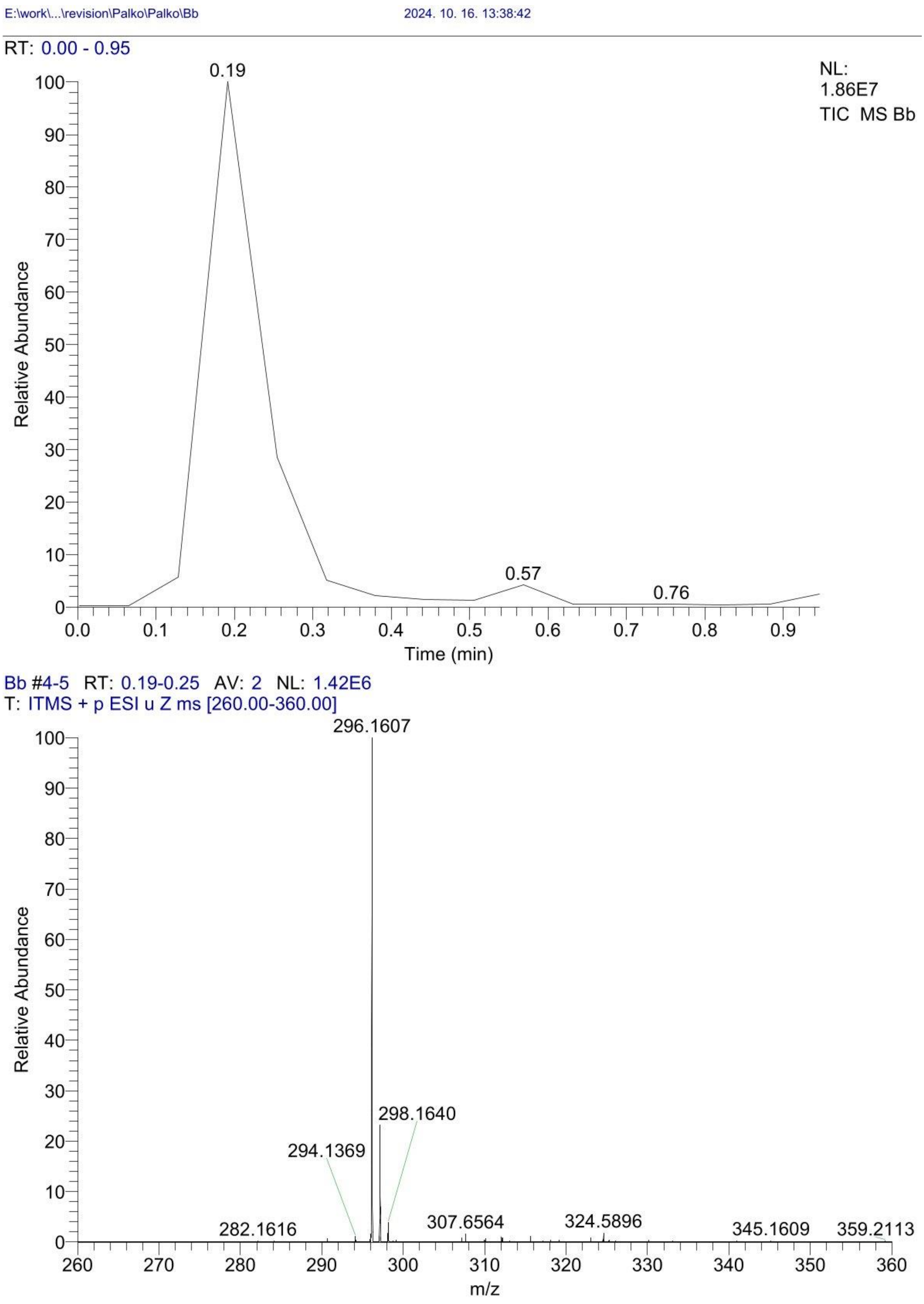

(1*R,2*R**,3*S**,4*S**)-3-((*Z*)-(7-chloro-2-oxoindolin-3-ylidene)amino)bicyclo[2.2.1]hept-5-ene-2-carboxamide (Bd)**

E:\work\...\revision\Palko\Palko\Bd 2024. 10. 16. 13:43:09

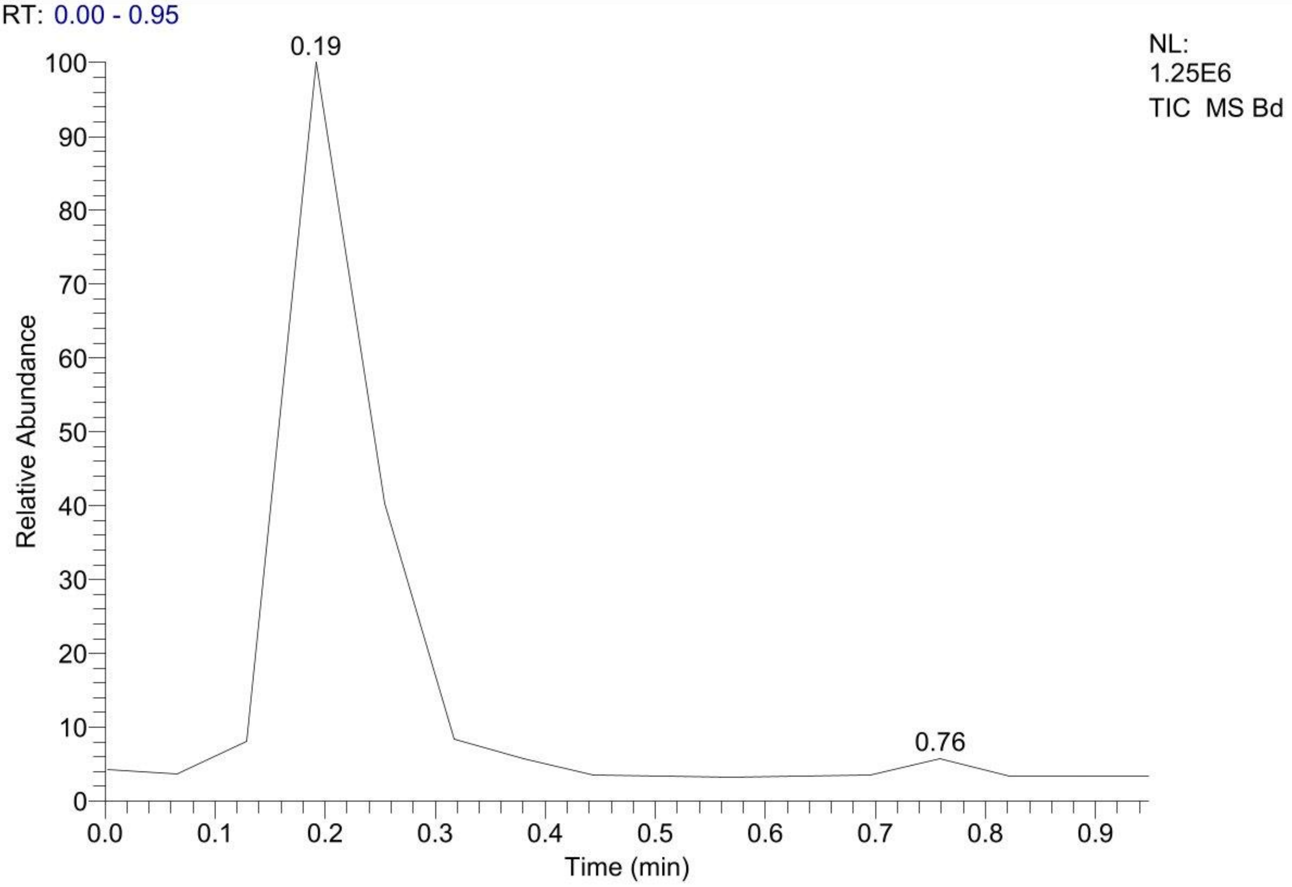

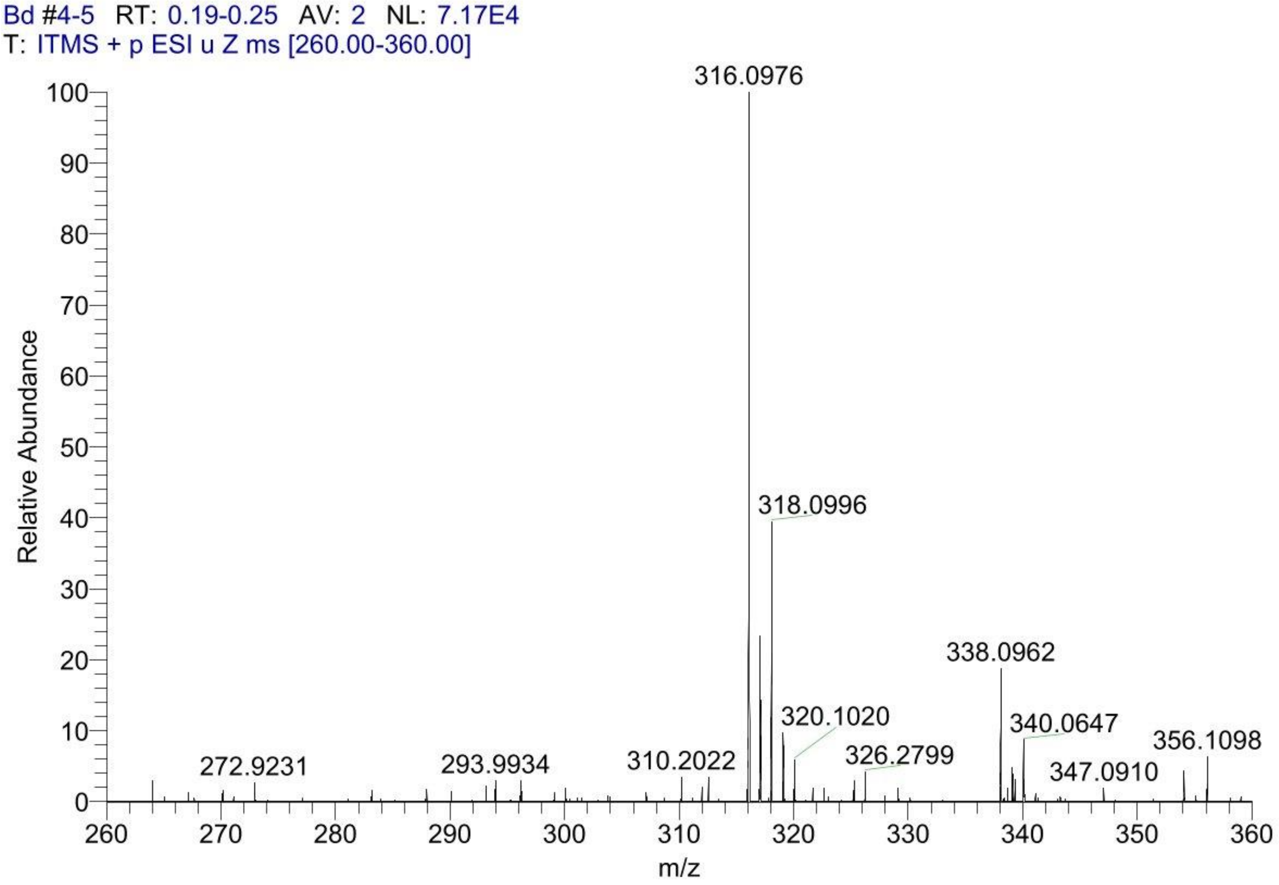

3. Docking studies

For docking studies, the macromolecules SARS-COV-2 main protease (PDB: 6LU6) and human mast cell tryptase (PDB: 2ZA5) were downloaded in pdb format from RSCB Protein Data Bank (https://www.rcsb.org). Preparing the macromolecules for docking was performed with AutoDock4[1] software. by following steps: cleaning the macromolecules from water residues, adding only the polar hydrogens, adding the Kollman charges, saving the macromolecules in pdbqt format. The ligand structures were optimized and minimized through Avogadro software. Docking protocols were accomplished by PyRx-Virtual[2] screening tool and iGEMDOCK[3] version 2.1 software. PyRx[2] is a virtual screening software for computational drug discovery that is perfect to screen libraries of compounds contrary to promising drug targets. The iGEMDOCK[3] is a graphical automatic drug design system for docking, screening and analysis. These programs are usually used to gauge the ligand conformation and orientation relative to the active site of the protein. *In silico* docking protocol studies were completed to assess the molecular interactions of target molecules with the SARS-COV-2 main protease (PDB: 6LU6) and human mast cell tryptase (PDB: 2ZA5) proteins with a co-crystallized ligand. The ligand interactions were visualized and analyzed through PyMOL 2.5[4] and Discovery Studio Visualizer (Biovia)[5]. Figures of binding ligands position of binding pocket of macromolecule H-bond interaction with distances in ångström were visualized in PyMOL 2.5[4] software. The 2D interactions diagrams of the ligand interaction with the residues of macromolecule were created utilising BIOVIA Discovery Studio[5] draw software.

The following steps were followed for the docking protocols through PyRx[2]:

- Preparation and optimization of the required protein and ligand files in the pdb format.
- Selection the macromolecule and ligands, making macromlolecule and ligands in Autodock tap.
- Define the grid box, in our case blind docking method was carried out, and ten best docking solutions will be obtained for analysis.
- RMSD value 0 docked pose was selected casewise.
- Validation of the molecular docking method was accomplished by redocking the cristallographic ligand of the target enzyme, against PDB ID: 6LU7 and PDB ID: 2ZA5, which checked the validation of docking calculations.

The following steps were followed for the docking simulations through iGEMDOCK[3]:

- Preparation and optimization of the required protein and ligand files in the .pdb format
- Blind docking method was carried out.
- Selection the docking accuracy settings for setting up the docking method where the stable docking mode was selected and ten best docking solutions will be obtained for analysis.

- Post docking interaction profile analysis of the best poses to determine the important residue interaction between the ligand and target protein active site residues.

Estimated total energy calculations were made with iGEMDOCK. The scoring function includes the following parameters of intermolecular and intramolecular energies and penalty if the ligand is out of range of the search box.

$E_{tot} = E_{inter} + E_{intra} + E_{penal.}$

References:

[1] Huey, R.; Morris, G.M.; Olson, A.J.; Goodsell, D.S., A J., *Computational Chemistry*, **2007**, *28*, 1145–1152.

[2] Dallakyan, S.; Olson, A.J., *Methods in molecular biology*, **2015**, *1263*, 243–250.

[3] Hsu, KC.; Chen, YF.; Lin, S.R.; Yang, J.M., *BMC Bioinformatics* , **2011**, *12*, (Suppl. 1. S33).

[4] The PyMOL Molecular Graphics System, Version 2.5 Schrödinger, LLC.

[5] BIOVIA, Dassault Systèmes, BIOVIA Discovery Studio, 4.5, San Diego: Dassault Systèmes, **2021**.

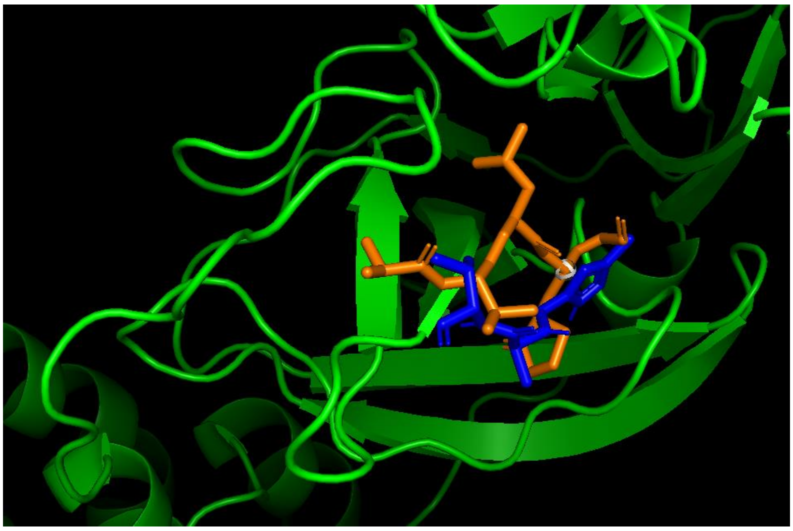

i

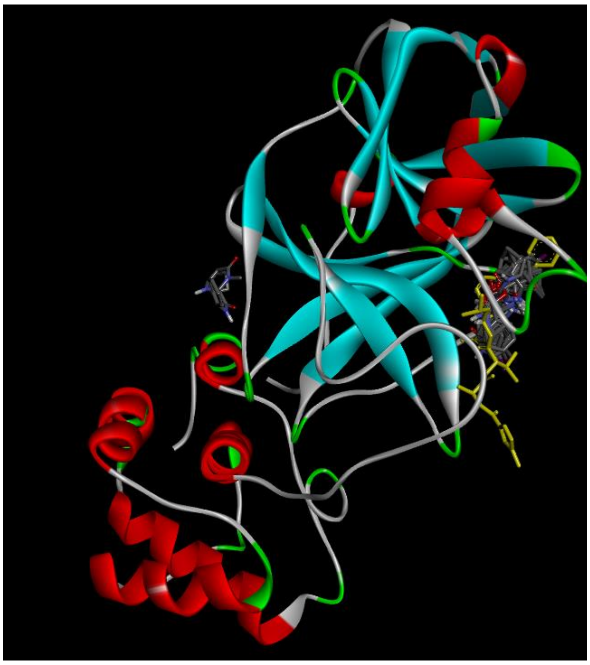

ii

Figure S1 *i*: Docking accuracy validation: binding poses of co-ligand of 6LU7 shown as orange sticks and the redocked one shown as blue.; *ii*: Binding pose of the original ligand (shown yellow) of SARS-COV-2 main protease (PDB: 6LU7) and ligand **3a–g, 3i–k, 3m** with 6LU7. There are two binding sites of 6LU7, that our molecules prefer. Compounds **3a–g, 3j–k, 3m** make interactions with the upper binding pocket, while **3i** rather interacts with the residues of the lower binding pocket of the 6LU7 macromolecule.

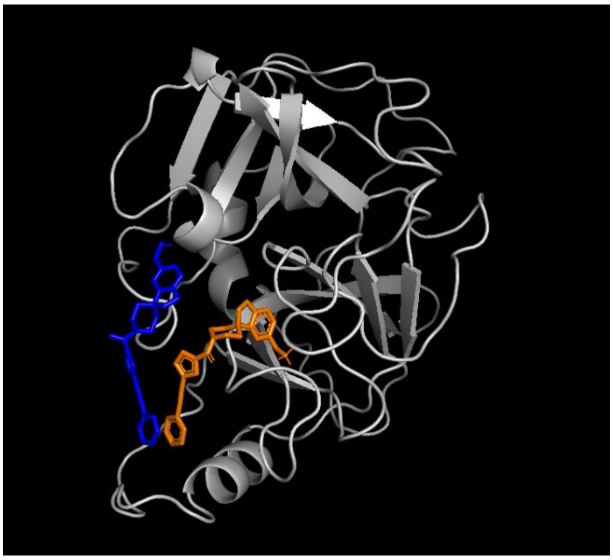

i

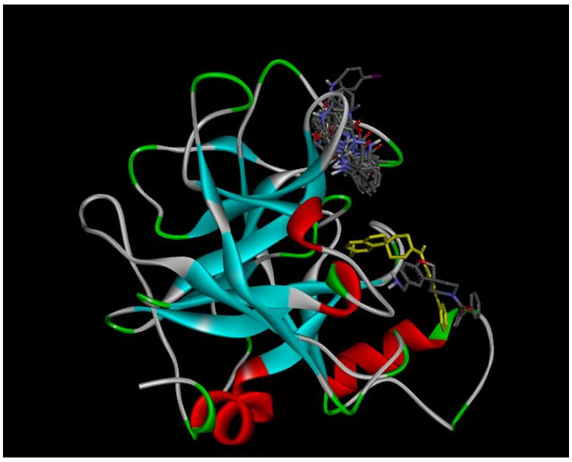

ii

Figure S2 *i*: Docking accuracy validation: binding poses of co-ligand of 2ZA5 shown as orange sticks and the redocked one shown as blue.; *ii*: Binding pose of the original ligand (shown yellow) of human tryptase with potent non-peptide inhibitor (PDB: 2ZA5) and ligand **3a–g, 3i–k, 3m** with 2ZA5. Our molecules **3a–g, 3j–k, 3m** prefer the same binding site of human mast cell tryptase (2ZA5).

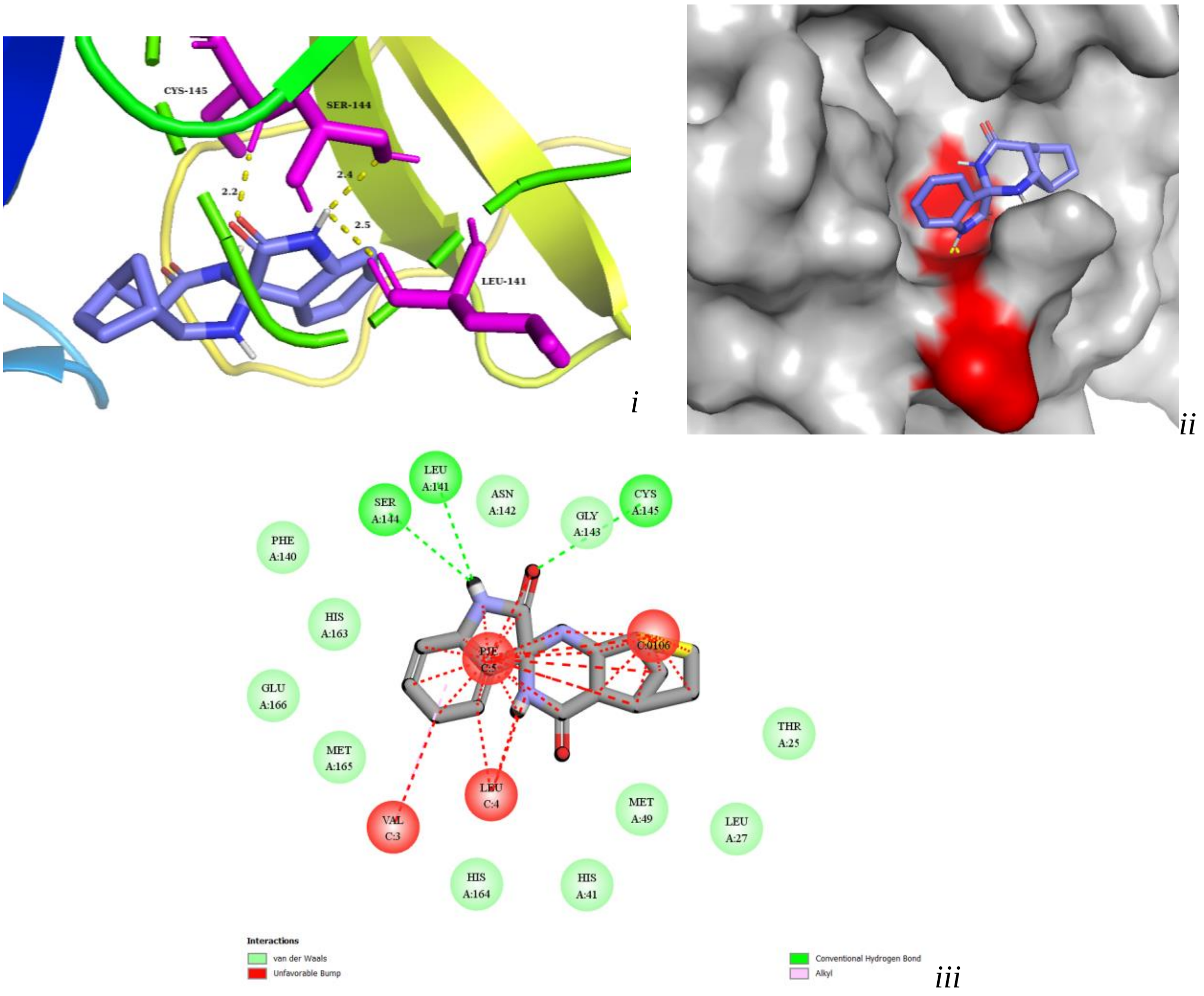

Figure S3 *i*: Molecular interactions and binding pose of compound **3a** at the interface of SARS-COV-2 main protease (PDB: 6LU7), H bonds between the macromolecule and compound **3a** shown as yellow dashes and distances in Å units; *ii*: Connelly surface of docking pose of 6LU7 with **3a** shown as stick model; *iii*: 2D interaction map between 6LU7 and **3a**.

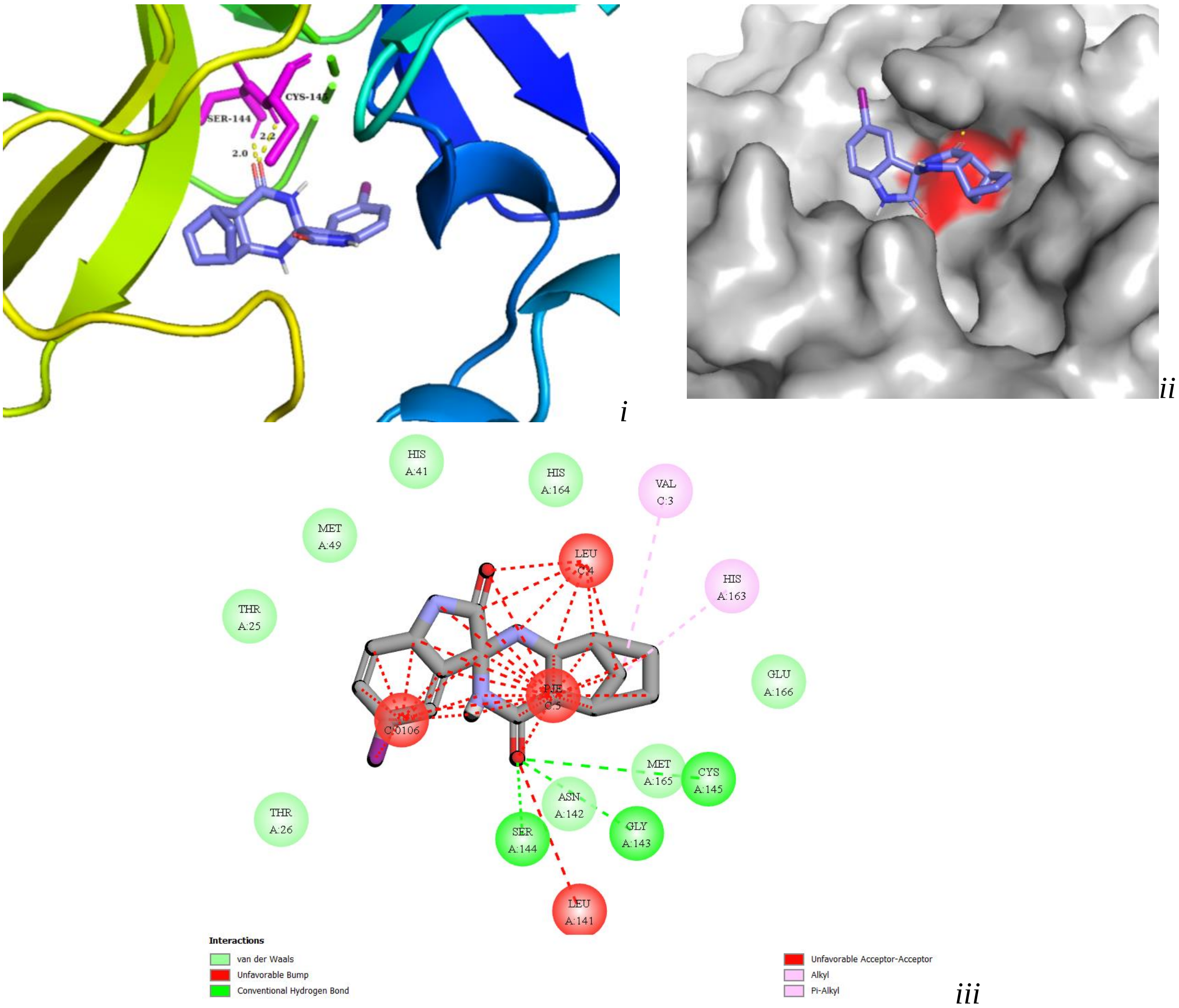

Figure S4 *i*: Molecular interactions and binding pose of compound **3c** at the interface of SARS-COV-2 main protease (PDB: 6LU7), H bonds between the macromolecule and compound **3c** shown as yellow dashes and distances in Å units; *ii*: Connelly surface of docking pose of 6LU7 with **3c** shown as stick model; *iii*: 2D interaction map between 6LU7 and **3c**.

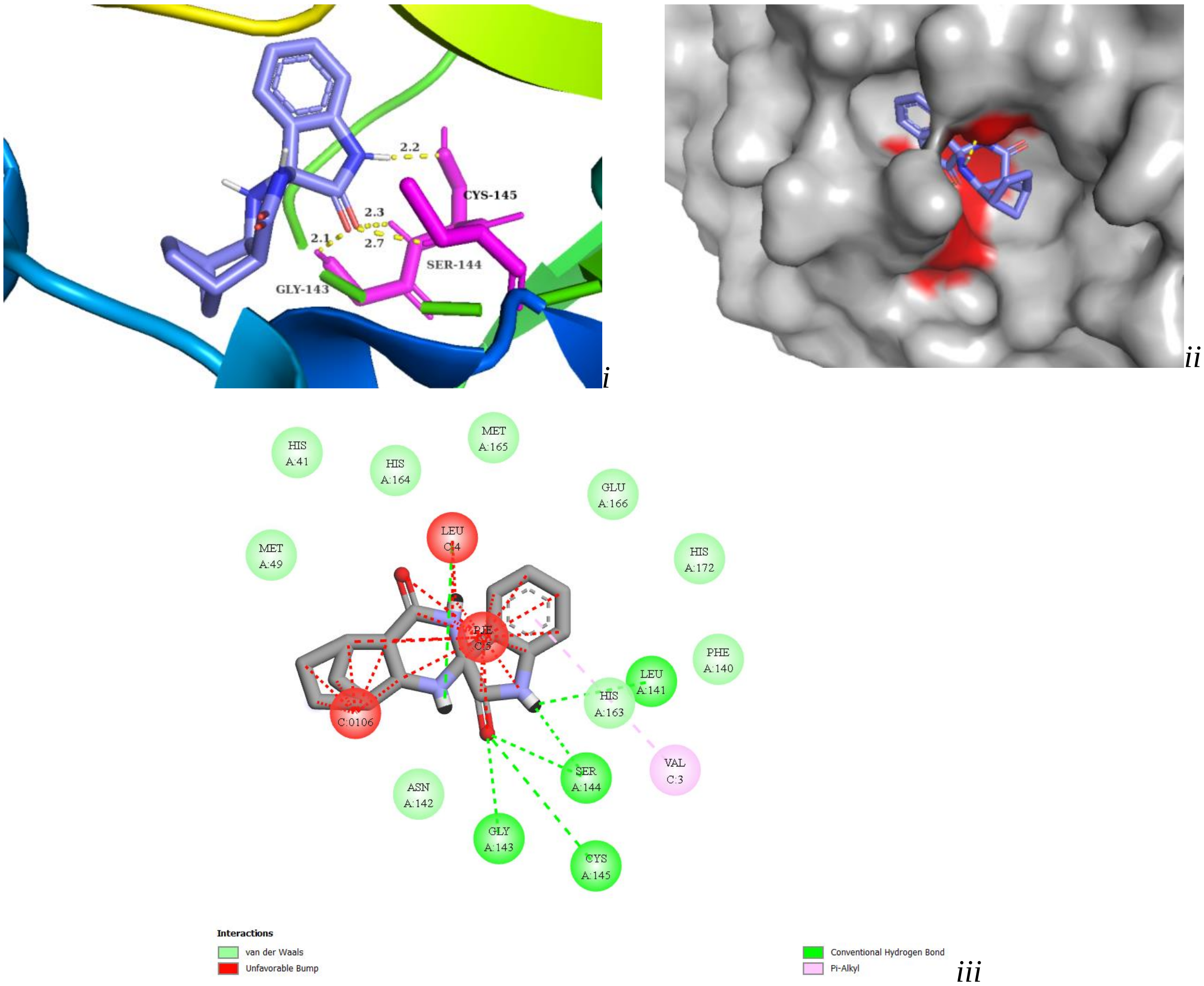

Figure S5 *i*: Molecular interactions and binding pose of compound **3e** at the interface of SARS-COV-2 main protease (PDB: 6LU7), H bonds between the macromolecule and compound **3e** shown as yellow dashes and distances in Å units; *ii*: Connelly surface of docking pose of 6LU7 with **3e** shown as stick model; *iii*: 2D interaction map between 6LU7 and **3e**.

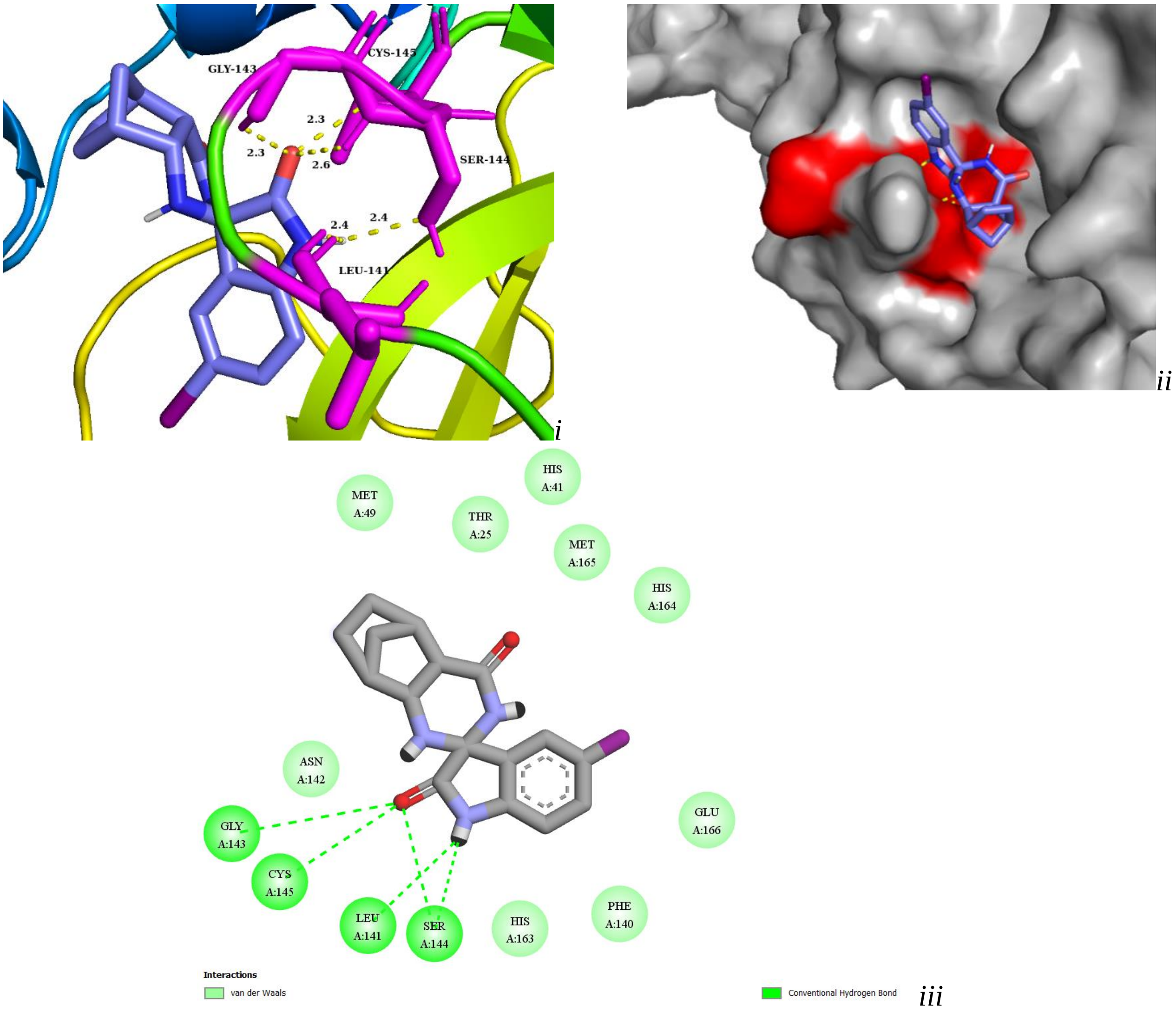

Figure S6 *i*: Molecular interactions and binding pose of compound **3g** at the interface of SARS-COV-2 main protease (PDB: 6LU7), H bonds between the macromolecule and compound **3g** shown as yellow dashes and distances in Å units; *ii*: Connelly surface of docking pose of 6LU7 with **3g** shown as stick model; *iii*: 2D interaction map between 6LU7 and **3g**.

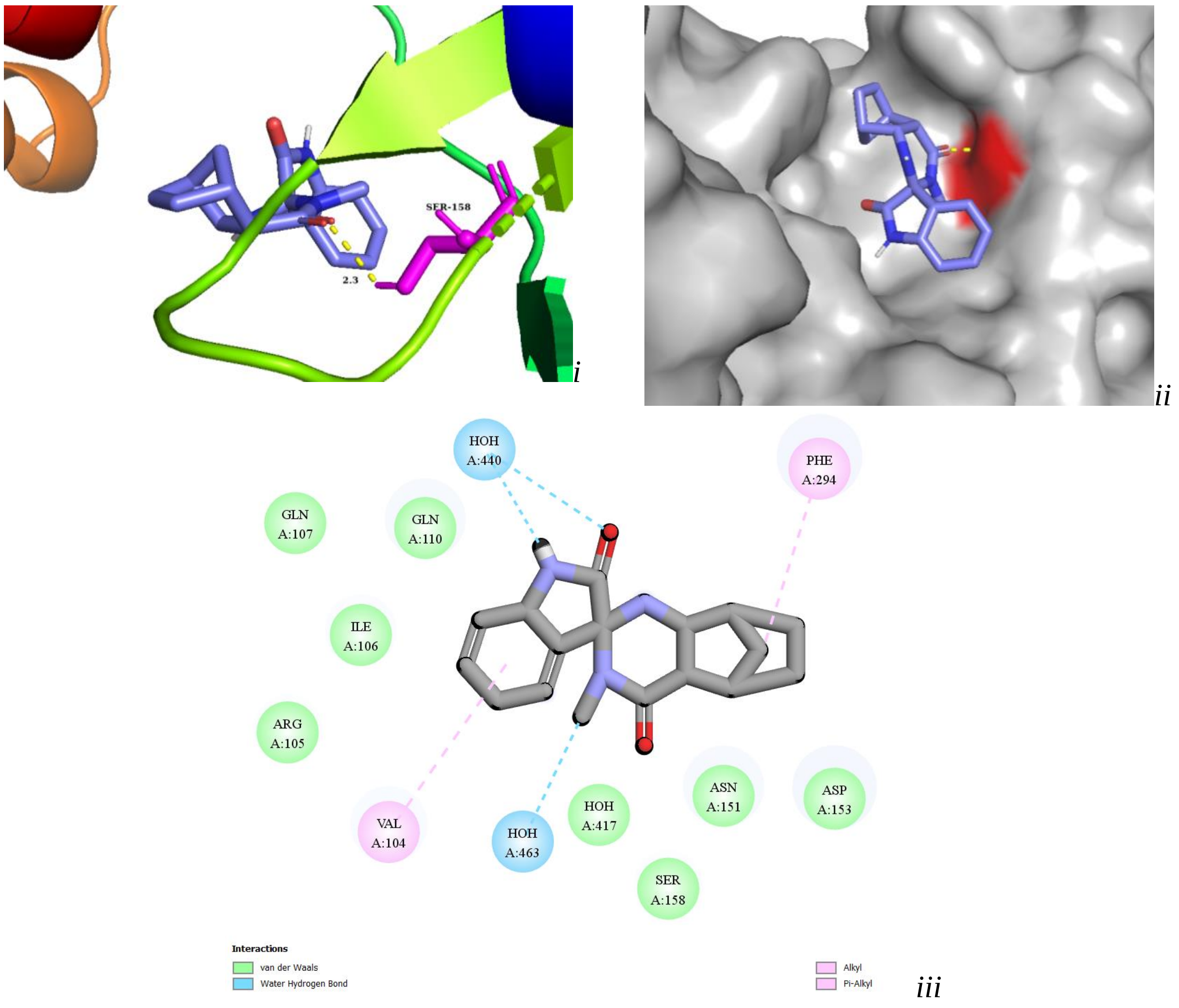

Figure S7 *i*: Molecular interactions and binding pose of compound **3i** at the interface of SARS-COV-2 main protease (PDB: 6LU7), H bonds between the macromolecule and compound **3i** shown as yellow dashes and distances in Å units; *ii*: Connelly surface of docking pose of 6LU7 with **3i** shown as stick model; *iii*: 2D interaction map between 6LU7 and **3i**.

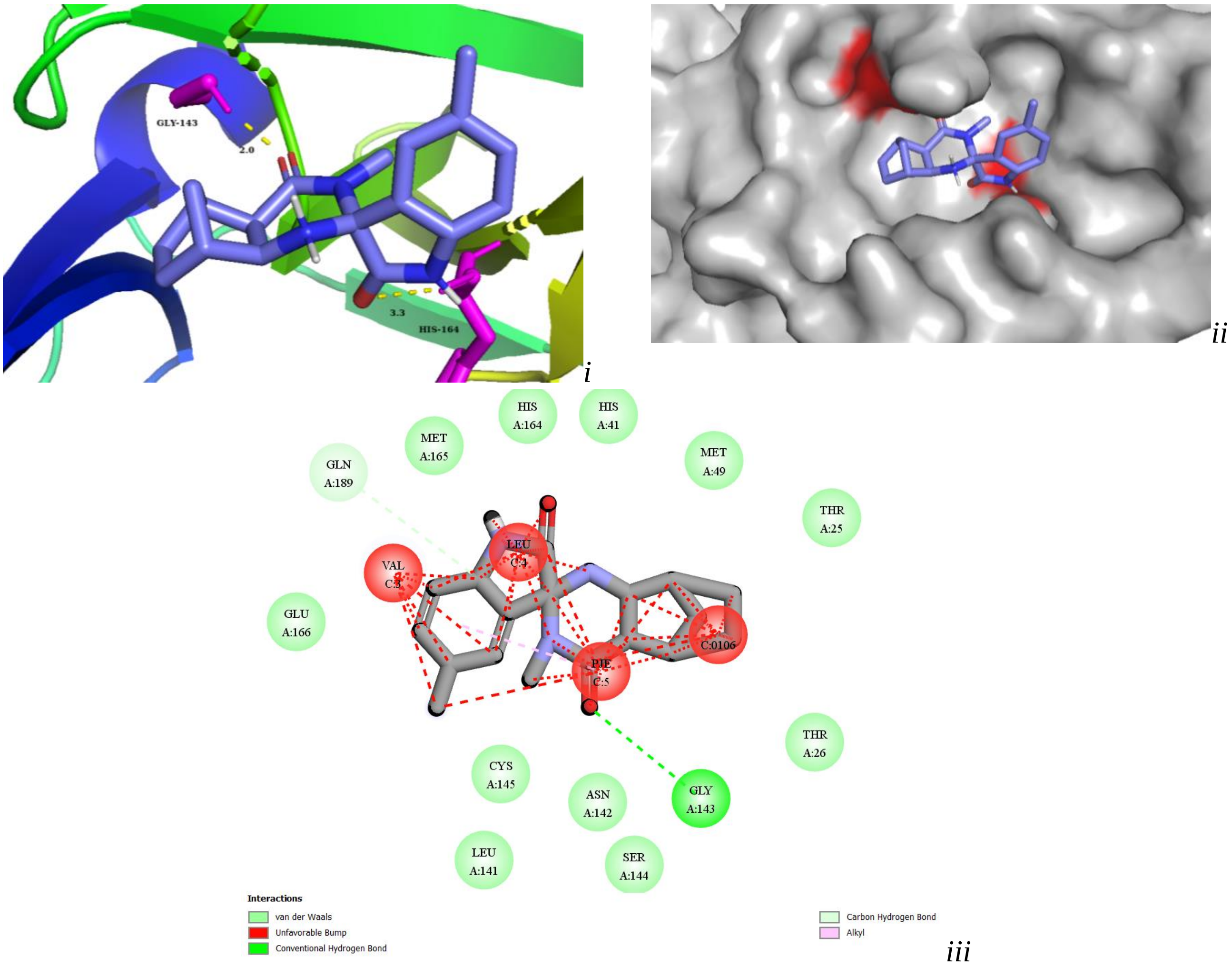

Figure S8 *i*: Molecular interactions and binding pose of compound **3j** at the interface of SARS-COV-2 main protease (PDB: 6LU7), H bonds between the macromolecule and compound **3j** shown as yellow dashes and distances in Å units; *ii*: Connelly surface of docking pose of 6LU7 with **3j** shown as stick model; *iii*: 2D interaction map between 6LU7 and **3j**.

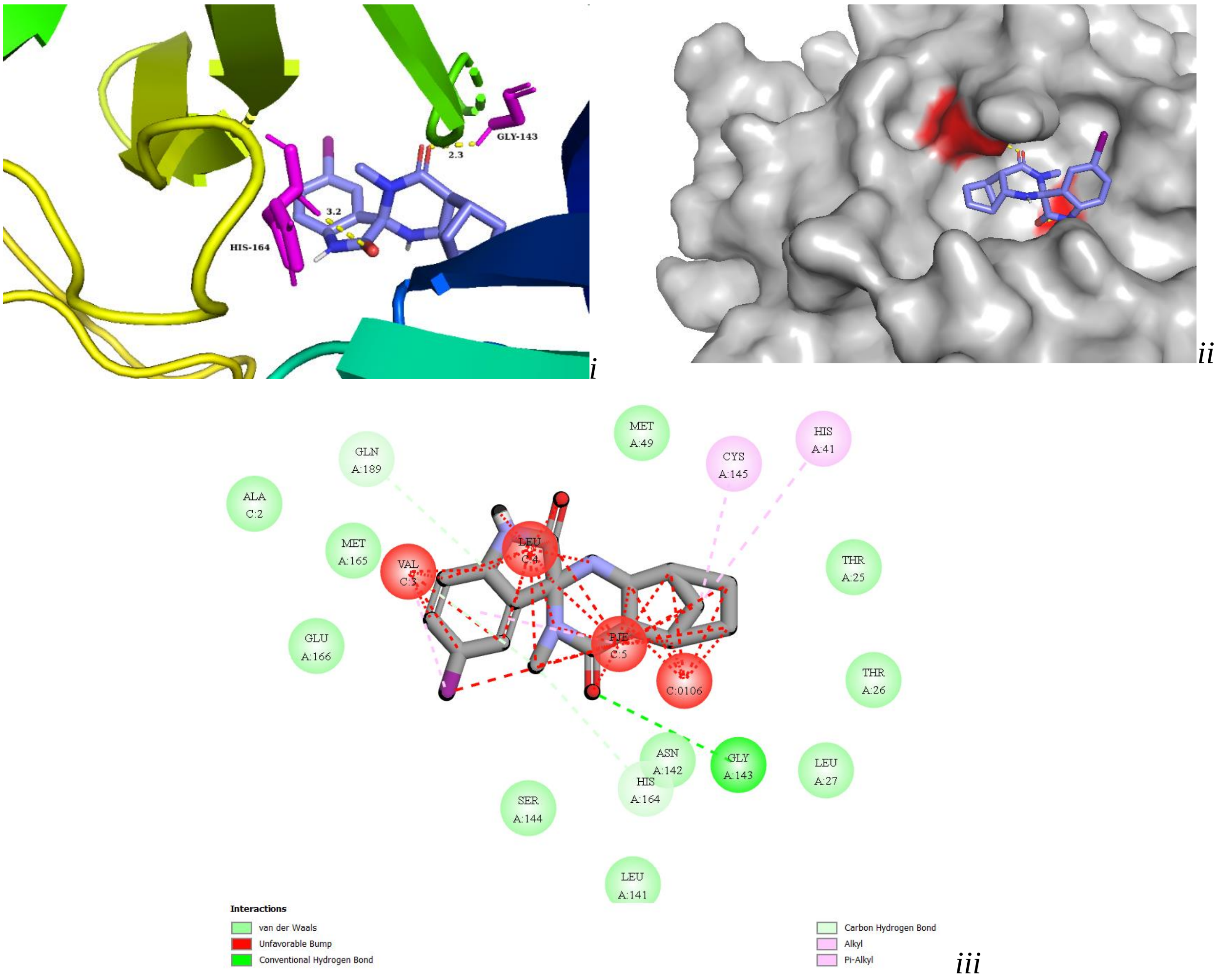

Figure S9 *i*: Molecular interactions and binding pose of compound **3k** at the interface of SARS-COV-2 main protease (PDB: 6LU7), H bonds between the macromolecule and compound **3k** shown as yellow dashes and distances in Å units; *ii*: Connelly surface of docking pose of 6LU7 with **3k** shown as stick model; *iii*: 2D interaction map between 6LU7 and **3k**.

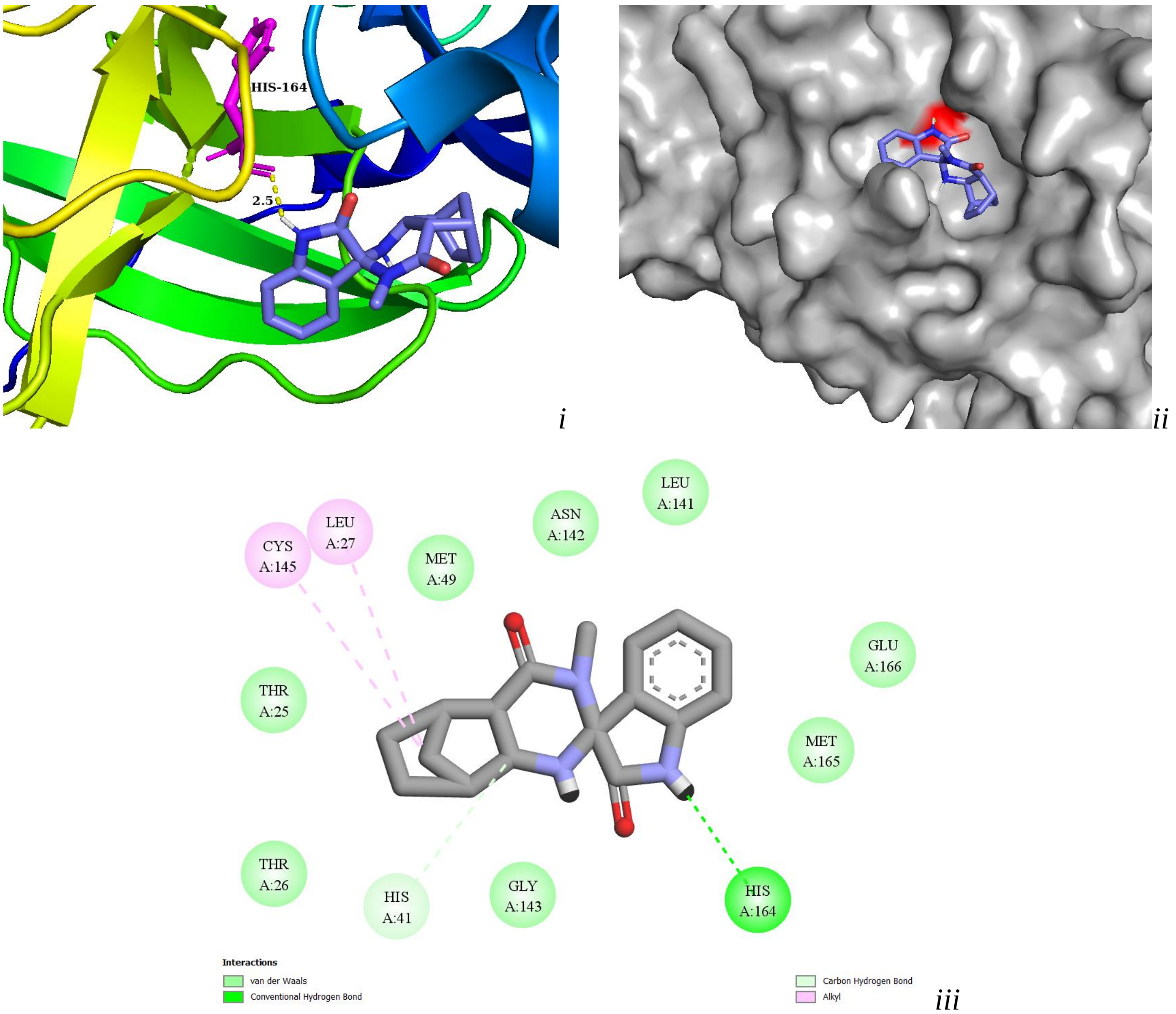

Figure S10 *i*: Molecular interactions and binding pose of compound **3m** at the interface of SARS-COV-2 main protease (PDB: 6LU7), H bonds between the macromolecule and compound **3m** shown as yellow dashes and distances in Å units; *ii*: Connelly surface of docking pose of 6LU7 with **3m** shown as stick model; *iii*: 2D interaction map between 6LU7 and **3m**.

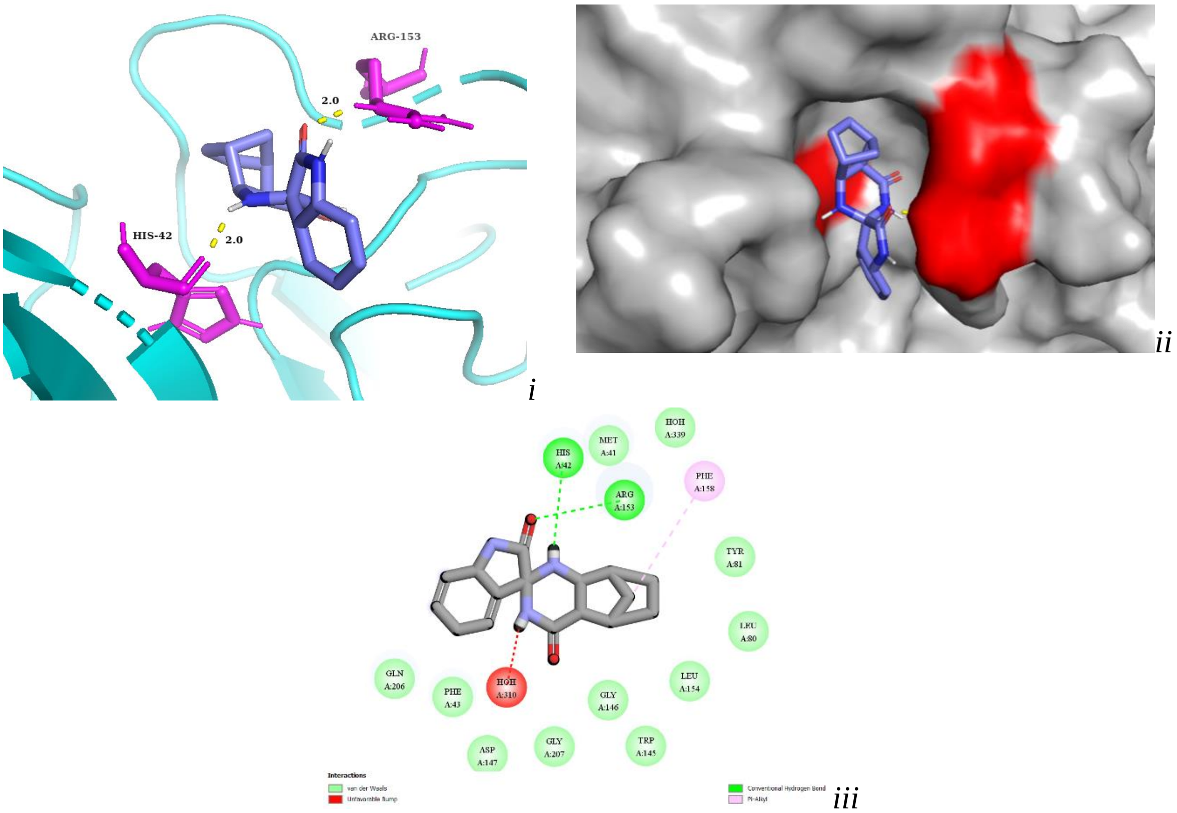

Figure S11 *i*: Molecular interactions and binding pose of compound **3a** at the interface of human tryptase with potent non-peptide inhibitor (PDB: 2ZA5); H bonds between the macromolecule and compound **3a** shown as yellow dashes and distances in Å units; *ii*: Connelly surface of docking pose of 2ZA5 with **3a** shown as stick model; *iii*: 2D interaction map between 2ZA5 and **3a**.

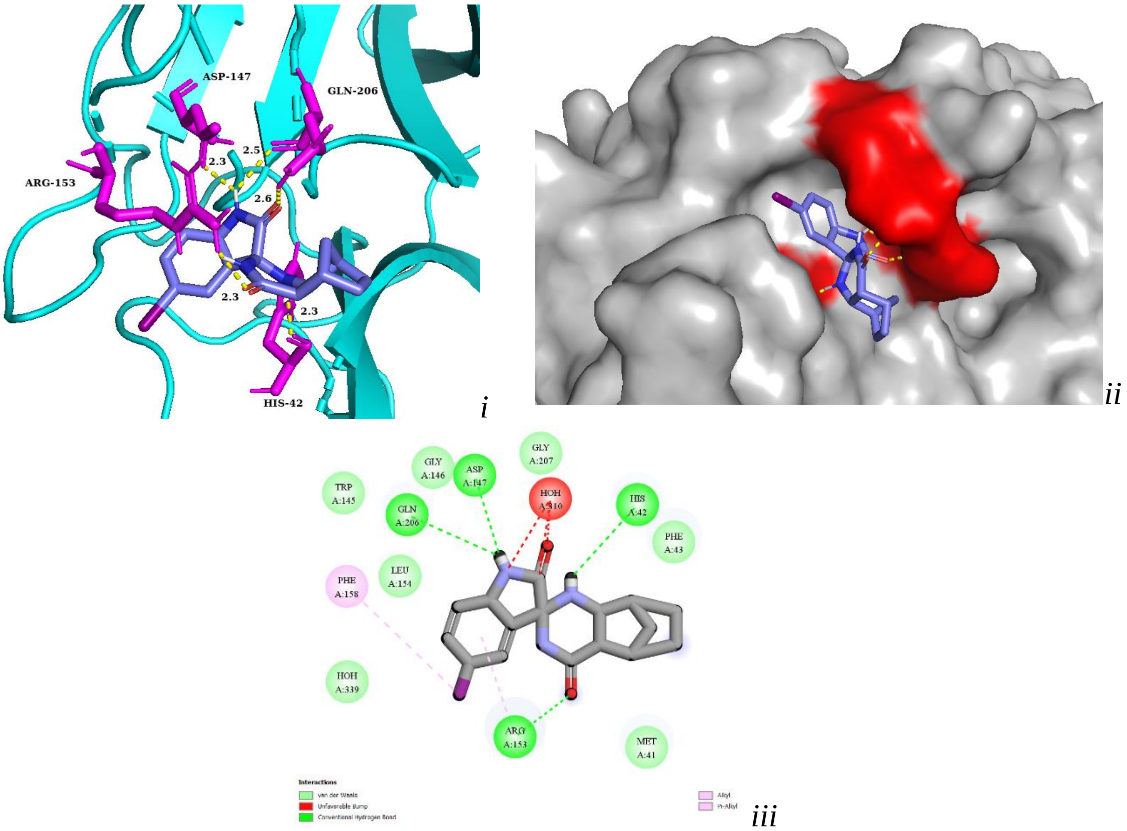

Figure S12 *i*: Molecular interactions and binding pose of compound **3c** at the interface of human tryptase with potent non-peptide inhibitor (PDB: 2ZA5); H bonds between the macromolecule and compound **3c** shown as yellow dashes and distances in Å units; *ii*: Connelly surface of docking pose of 2ZA5 with **3c** shown as stick model; *iii*: 2D interaction map between 2ZA5 and **3c**.

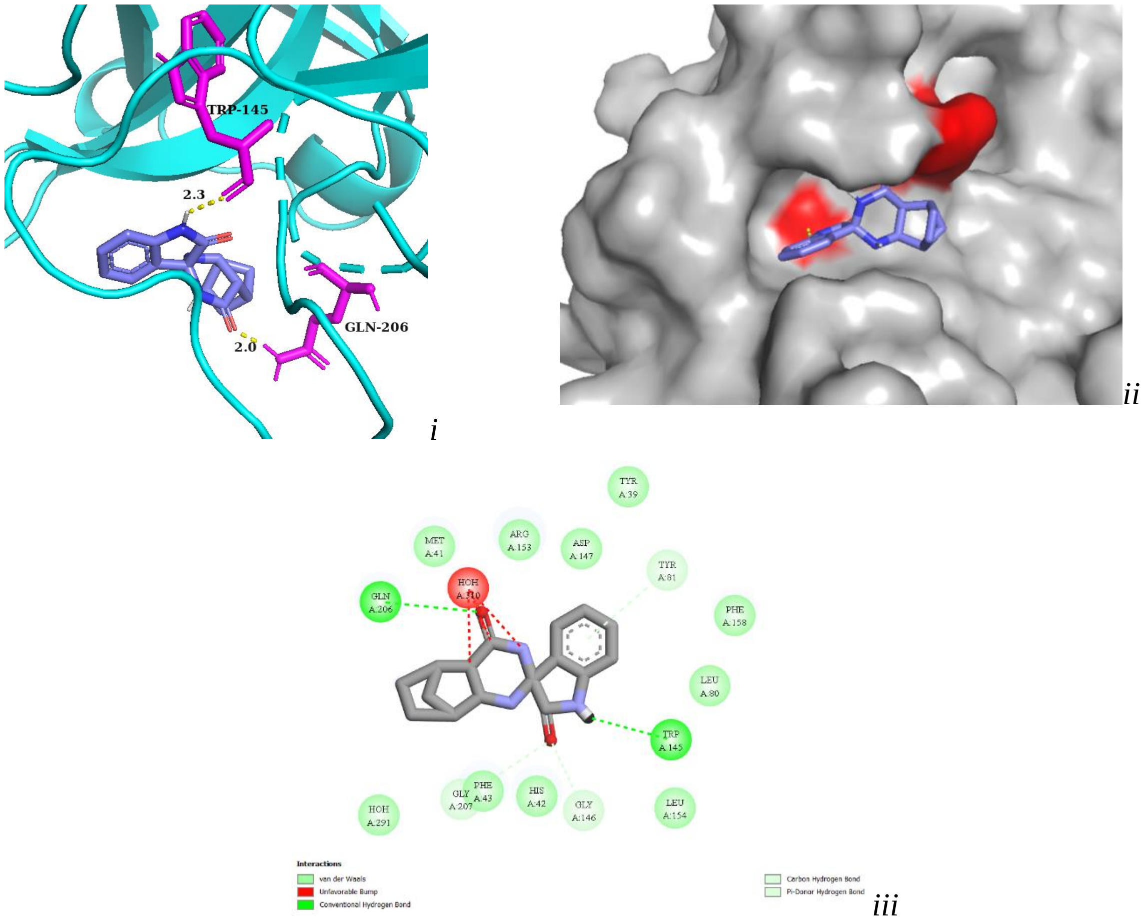

Figure S13 *i*: Molecular interactions and binding pose of compound **3e** at the interface of human tryptase with potent non-peptide inhibitor (PDB: 2ZA5); H bonds between the macromolecule and compound **3e** shown as yellow dashes and distances in Å units; *ii*: Connelly surface of docking pose of 2ZA5 with **3e** shown as stick model; *iii*: 2D interaction map between 2ZA5 and **3e**.

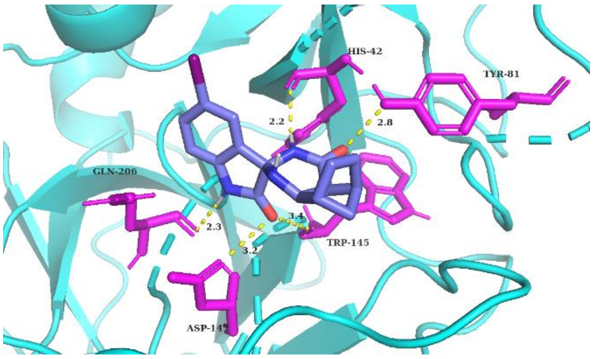

i

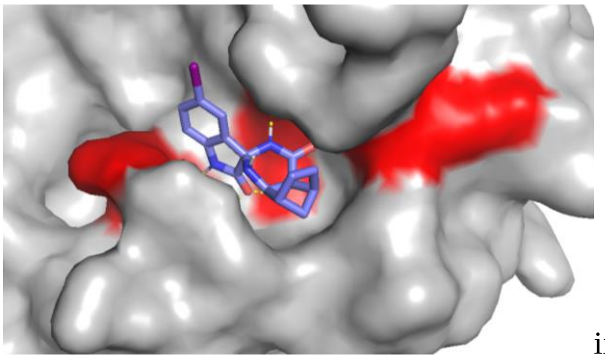

ii

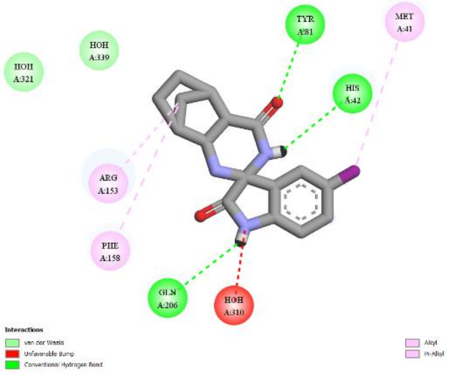

iii

Figure S14 *i*: Molecular interactions and binding pose of compound **3g** at the interface of human tryptase with potent non-peptide inhibitor (PDB: 2ZA5); H bonds between the macromolecule and compound **3g** shown as yellow dashes and distances in Å units; *ii*: Connelly surface of docking pose of 2ZA5 with **3g** shown as stick model; *iii*: 2D interaction map between 2ZA5 and **3g**.

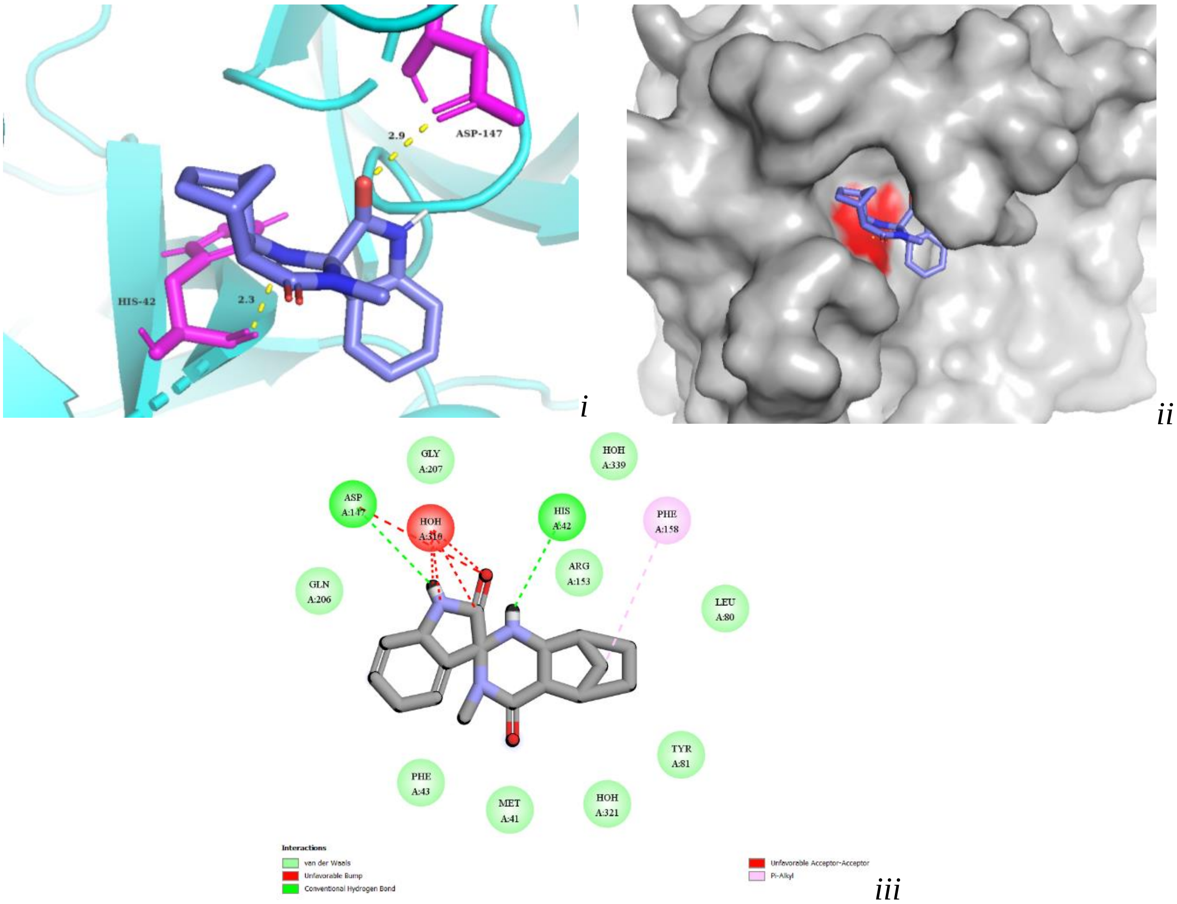

Figure S15 *i*: Molecular interactions and binding pose of compound **3i** at the interface of human tryptase with potent non-peptide inhibitor (PDB: 2ZA5); H bonds between the macromolecule and compound **3i** shown as yellow dashes and distances in Å units; *ii*: Connelly surface of docking pose of 2ZA5 with **3i** shown as stick model; *iii*: 2D interaction map between 2ZA5 and **3i**.

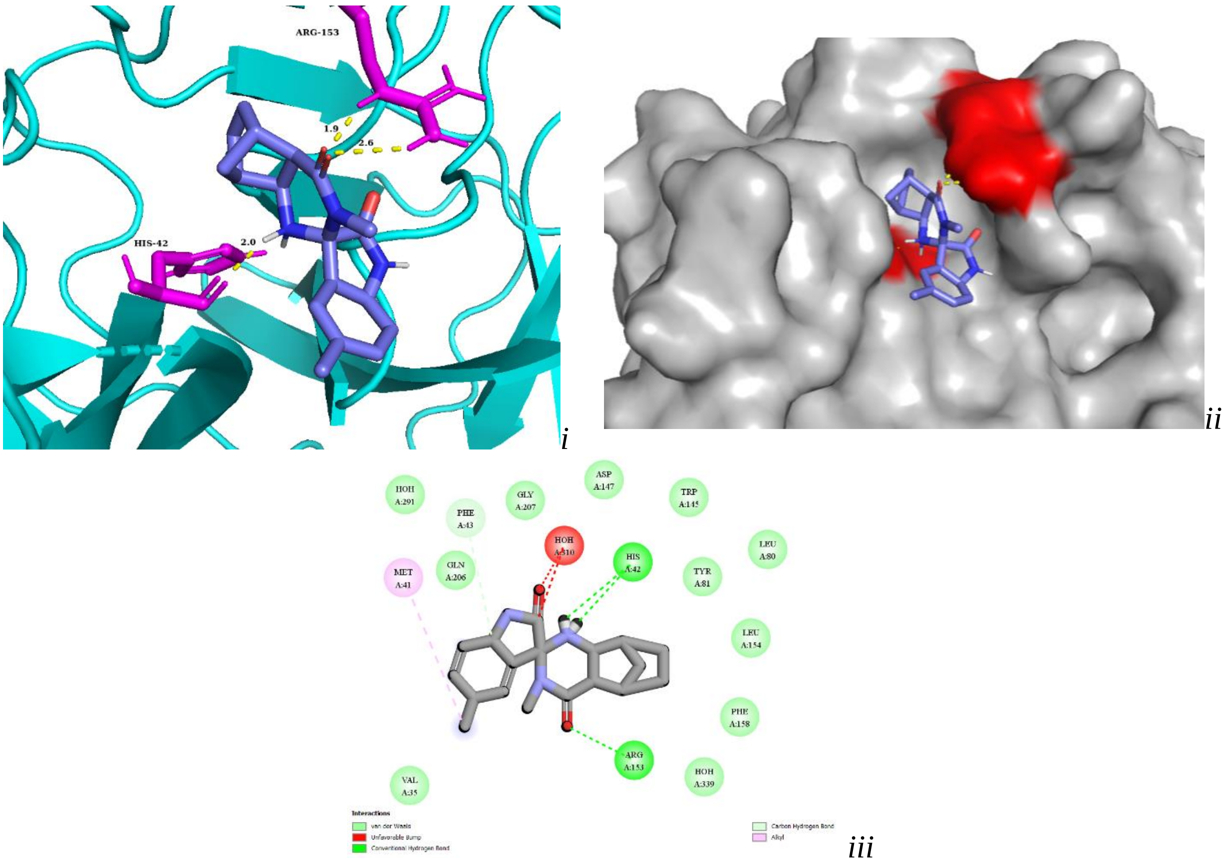

Figure S16 *i*: Molecular interactions and binding pose of compound **3j** at the interface of human tryptase with potent non-peptide inhibitor (PDB: 2ZA5); H bonds between the macromolecule and compound **3j** shown as yellow dashes and distances in Å units; *ii*: Connelly surface of docking pose of 2ZA5 with **3j** shown as stick model; *iii*: 2D interaction map between 2ZA5 and **3j**.

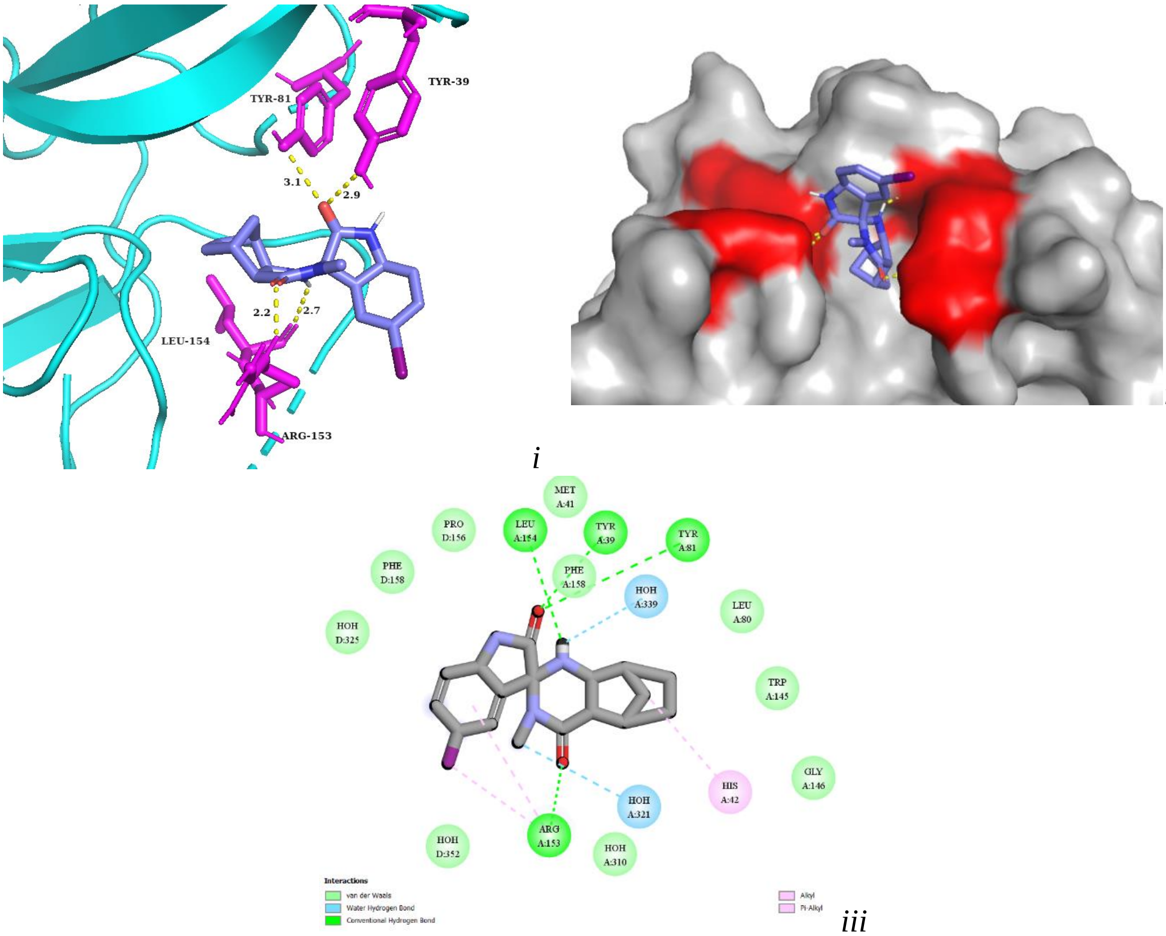

Figure S17 *i*: Molecular interactions and binding pose of compound **3k** at the interface of human tryptase with potent non-peptide inhibitor (PDB: 2ZA5); H bonds between the macromolecule and compound **3k** shown as yellow dashes and distances in Å units; *ii*: Connelly surface of docking pose of 2ZA5 with **3k** shown as stick model; *iii*: 2D interaction map between 2ZA5 and **3k**.

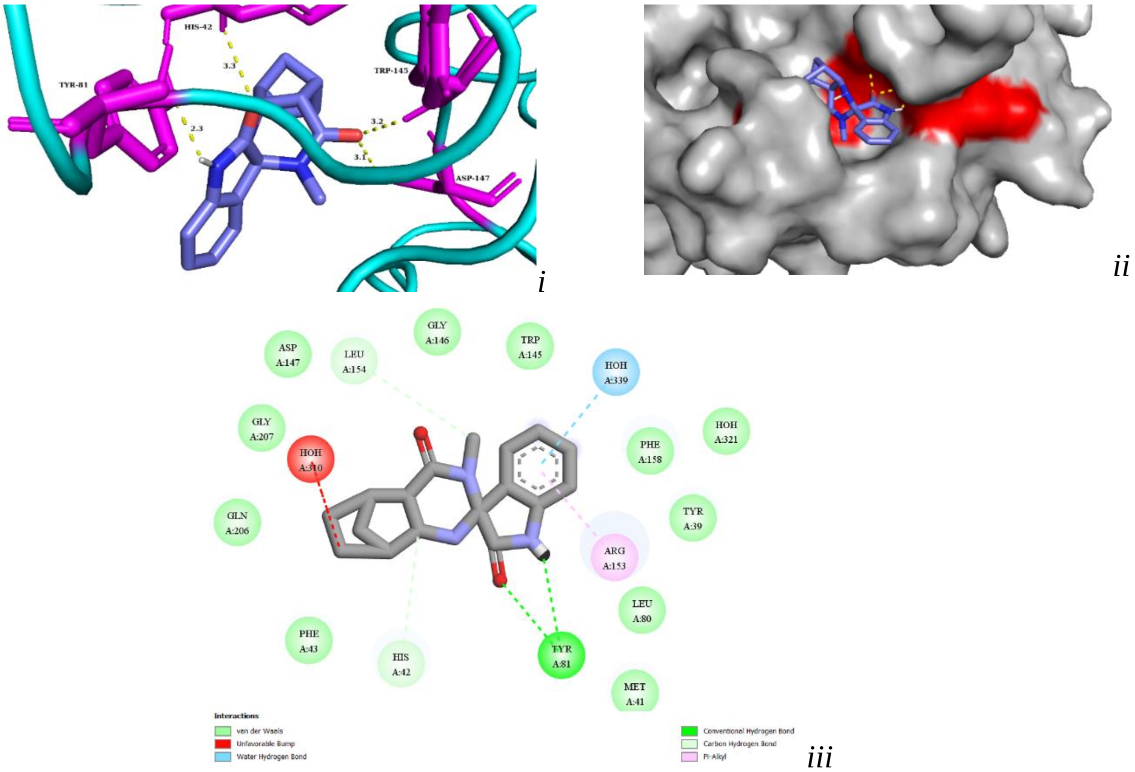

Figure S18 *i*: Molecular interactions and binding pose of compound **3m** at the interface of human tryptase with potent non-peptide inhibitor (PDB: 2ZA5); H bonds between the macromolecule and compound **3m** shown as yellow dashes and distances in Å units; *ii*: Connelly surface of docking pose of 2ZA5 with **3m** shown as stick model; *iii*: 2D interaction map between 2ZA5 and **3m**.

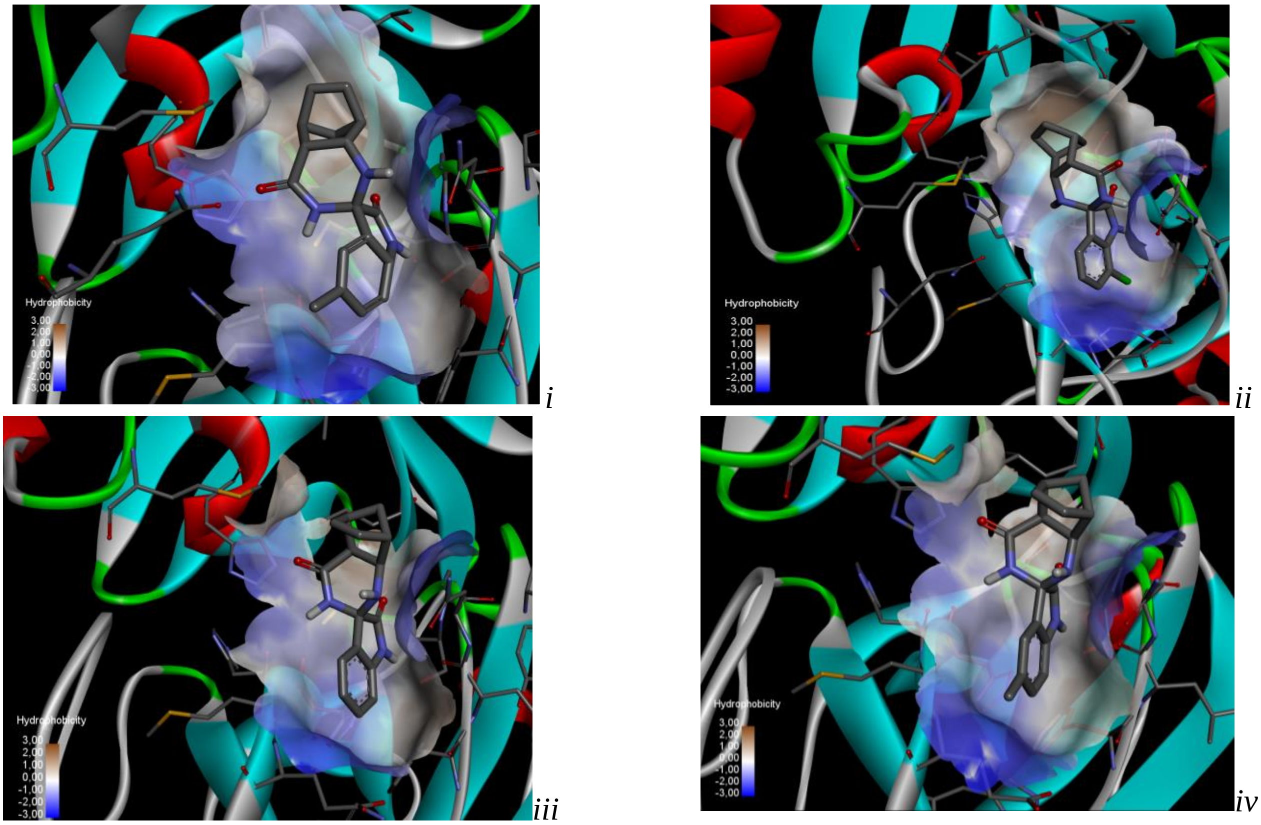

Figure S19 *i*: Receptor surface: Hydrophobicity of the surrounding amino acids around the binding pose of compound **3b** at the upper binding pocket of SARS-COV-2 main protease (PDB: 6LU7); *ii*: Receptor surface: Hydrophobicity of the surrounding amino acids around the binding pose of compound **3d** at the upper binding pocket of 6LU7; *iii*: Receptor surface: Hydrophobicity of the surrounding amino acids around binding pose of compound **3e** at the upper binding pocket of 6LU7; *iv*: Receptor surface: Hydrophobicity of the surrounding amino acids around the binding pose of compound **3f** at the upper binding pocket of 6LU7.

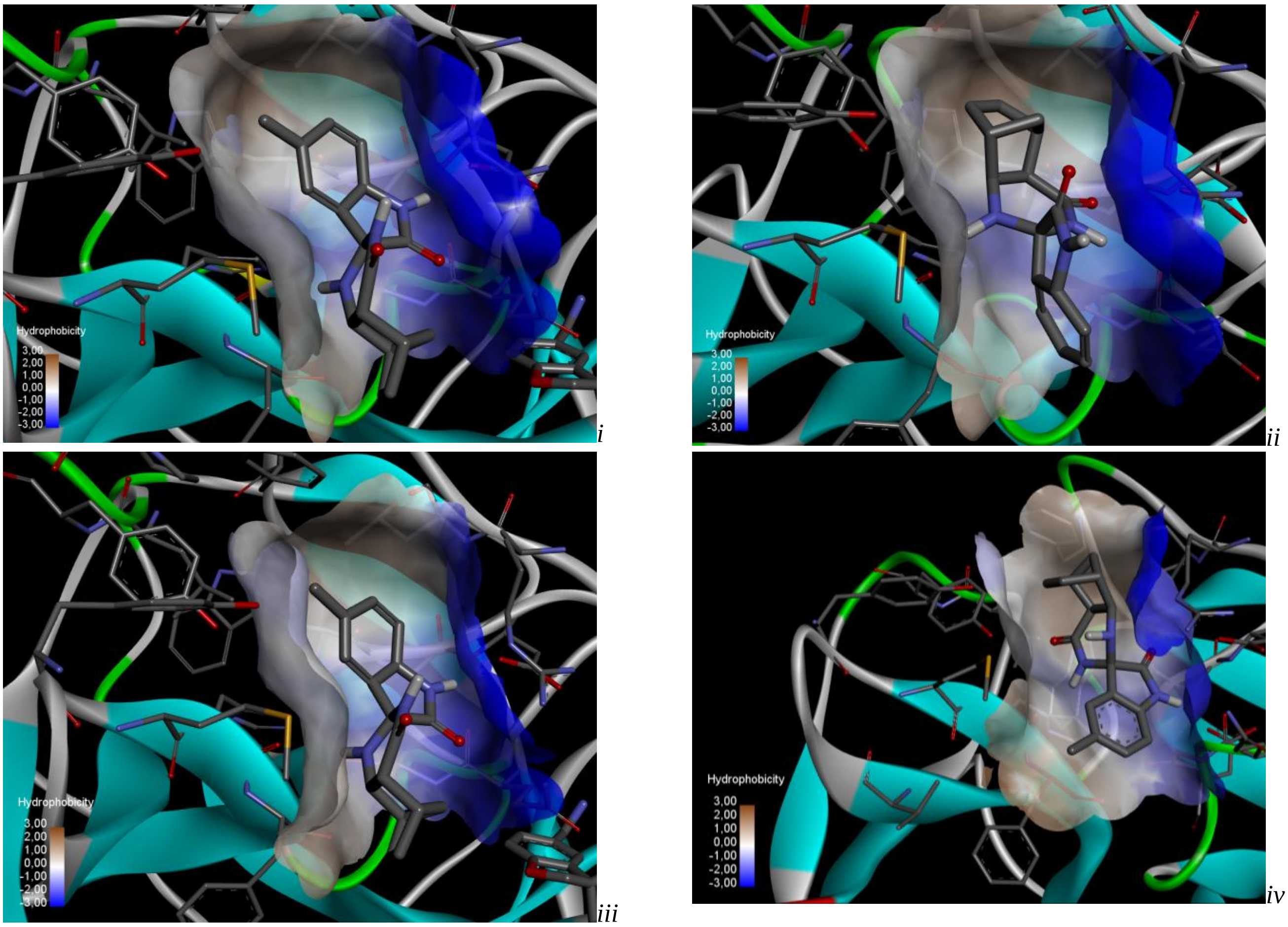

Figure S20 *i*: Receptor surface: Hydrophobicity of the surrounding amino acids around the binding pose of compound **3a** at the binding pocket of human mast cell tryptase (PDB: 2ZA5); *ii*: Receptor surface: Hydrophobicity of the surrounding amino acids around the binding pose of compound **3b** at the binding pocket of human mast cell tryptase (PDB: 2ZA5); *iii*: Receptor surface: Hydrophobicity of the surrounding amino acids around the binding pose of compound **3d** at the binding pocket of 2ZA5; *iv*: Receptor surface: Hydrophobicity of the surrounding amino acids around binding pose of compound **3f** at the binding pocket of 2ZA5.

4. SwissADME pharmacokinetic properties prediction

Table S1 Prediction of *in-silico* lipophilicity, water solubility and pharmacokinetic parameters of novel spiro[5,8-methanoquinazoline-2,3'-indoline]-2',4-dione derivatives **3a−g, 3i−k, 3m** by SwissADME web tool.

				**Pharmacokinetic parameters**			
**Molecules**	**Structure**	**Lipophilicity (Consensus logP$_{ow}$)**	**Water solubility logS$_w$**	**GI**	**BBB**	**CYP2D6**	**Skin permeation logK$_p$ (cm/s)**
**3a**	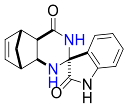	1.80	−3.69 soluble	High	No	Yes	−7.74
**3b**	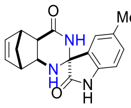	2.12	−4.07 moderately soluble	High	No	Yes	−7.57
**3c**	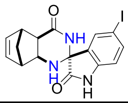	1.93	−4.56 moderately soluble	High	No	Yes	−8.05
**3d**	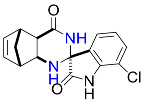	2.06	−4.29 moderately soluble	High	No	Yes	−7.51
**3e**	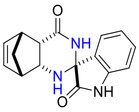	0.81	−3.69 soluble	High	No	Yes	−7.74
**3f**	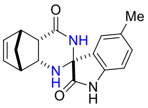	1.14	−4.07 moderately soluble	High	No	Yes	−7.57
**3g**	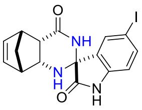	1.46	−4.56 moderately soluble	High	No	Yes	−8.05
**3i**	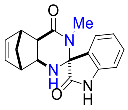	2.17	−3.35 soluble	High	No	Yes	−7.70
**3j**	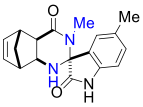	2.45	−3.73 soluble	High	No	Yes	−7.53
**3k**	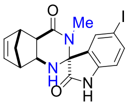	2.45	−4.21 moderately soluble	High	No	Yes	−8.00
**3m**	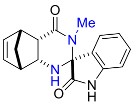	2.30	−3.35 soluble	High	No	Yes	−7.70

Table S2 Prediction of *in-silico* druglikeness properties of novel spiro[5,8-methanoquinazoline-2,3'-indoline]-2',4-dione derivatives **3a–g, 3i–k, 3m** by SwissADME web tool.

		Druglikeness properties				
**Molecules**	**Structure**	**Lipinsky**	**Ghore**	**Veber**	**Egan**	**Muegge**
**3a**	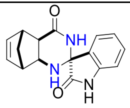	Yes<br>0 violation	No<br>1 violation	Yes	Yes	Yes
**3b**	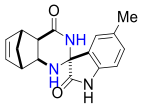	Yes<br>0 violation	No<br>1 violation	Yes	Yes	Yes
**3c**	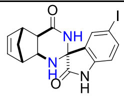	Yes<br>0 violation	Yes	Yes	Yes	Yes
**3d**	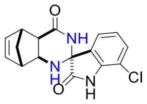	Yes<br>0 violation	Yes	Yes	Yes	Yes
**3e**	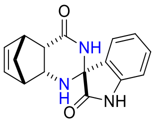	Yes<br>0 violation	No<br>1 violation	Yes	Yes	Yes
**3f**	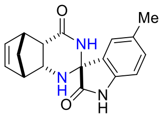	Yes<br>0 violation	No<br>1 violation	Yes	Yes	Yes
**3g**	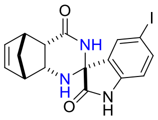	Yes<br>0 violation	Yes	Yes	Yes	Yes
**3i**	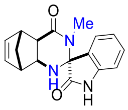	Yes<br>0 violation	Yes	Yes	Yes	Yes
**3j**	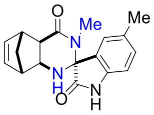	Yes<br>0 violation	Yes	Yes	Yes	Yes
**3k**	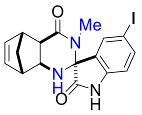	Yes<br>0 violation	Yes	Yes	Yes	Yes
**3m**	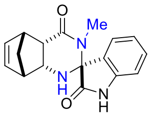	Yes<br>0 violation	Yes	Yes	Yes	Yes
